# Synthesis of acremines A, B and F and studies on the bisacremines

**DOI:** 10.3762/bjoc.15.219

**Published:** 2019-09-23

**Authors:** Nils Winter, Dirk Trauner

**Affiliations:** 1 Department of Chemistry, University of Munich, Butenandtstraße 5–13, 81377 Munich, Germany; 2 Department of Chemistry, New York University, 100 Washington Square East, Room 712, New York, NY 10003, USA

**Keywords:** meroterpenoid, natural product, selective oxidation, total synthesis

## Abstract

The acremines are a family of meroterpenoids isolated from fungi of the genus *Acremonium*. Here, we present the asymmetric total synthesis of acremine F which hinges on a modestly enantioselective dihydroxylation and a subsequent kinetic resolution via a highly selective asymmetric reduction. Chemoselective oxidation of acremine F gave access to acremines A and B. The dimerization of acremine F to bisacremine E was investigated but could not be achieved, shedding light on the formation of the acremine dimers in nature.

## Introduction

**Figure 1:**
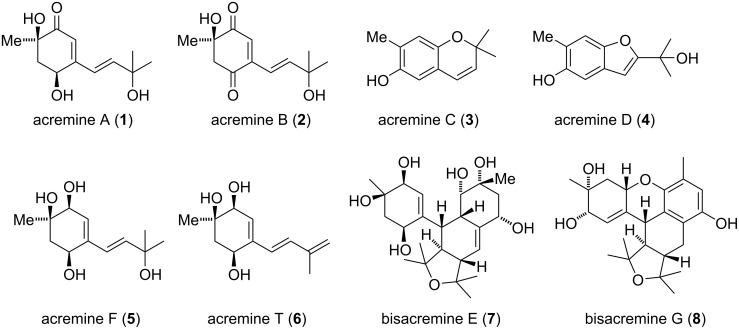
Selected members of the acremine family [3–5].

Endophytic fungi grow in a symbiotic relationship with their plant hosts [1], which is mediated by secondary metabolites [2]. In 2005, Torta and co-workers reported the isolation of six meroterpenoid natural products, acremines A–F from A20, a strain of *Acreonium byssoides*, isolated from grapevine leaves that were artificially inoculated with *Plasmopora viticola* (Figure 1) [3]. This class of natural products is characterized by a highly substituted cyclohexene core, featuring up to three stereogenic carbons, which is linked to a prenyl unit. Nature achieves further diversification by several modes of oxidation and cyclization. While acremine F (**5**) exhibited no significant bioactivity, acremines A–D showed inhibition of *P. viticola sporangia* germination.

In 2015, Wei and co-workers discovered bisacremines E–G, the most complex members of the acremine family, from the soil-derived strain *A. persicinum SC0105* [4]. These natural products are presumed to be derived from two acremine F (**5**) units by a formal [4 + 2] cycloaddition followed by condensation and oxidation.

Given the diversity and structural beauty of this class of natural products, it is not surprising that the acremine family has attracted the attention of the synthetic community [6–8]. Nevertheless, to the best of our knowledge, no asymmetric entry to this class of natural products has been described.

**Scheme 1:**
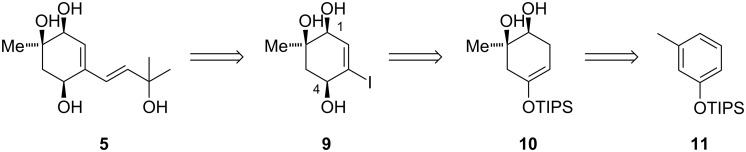
Retrosynthetic analysis of acremine F (**5**).

Our retrosynthetic analysis of **5** is depicted in Scheme 1. The prenyl side chain would be introduced by transition metal-catalyzed cross coupling of vinyl iodide **9**. Compound **9** in turn could be traced back to silyl enol ether **10**. *Ent*-**10** was first reported by Herzon and co-workers [9] and is derived from phenol silyl ether **11** via Birch reduction and dihydroxylation.

## Results and Discussion

**Scheme 2:**
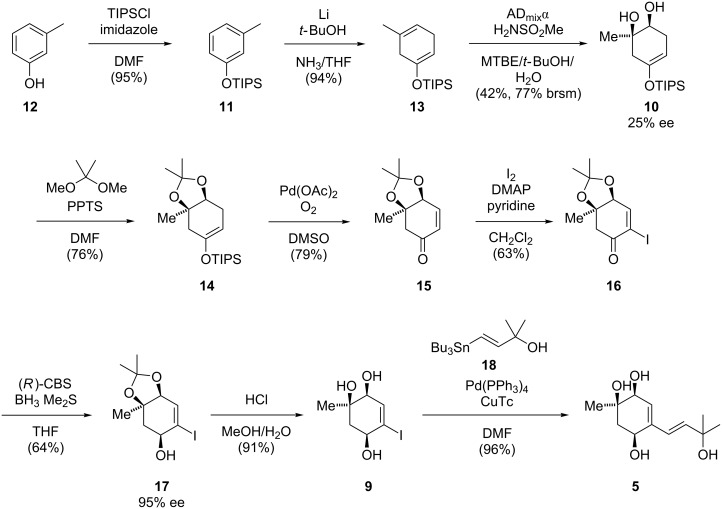
Total synthesis of acremine F (**5**).

Our synthesis started with *meta*-cresol (**12**) which was protected as a TIPS ether and then subjected to Birch reduction conditions to afford cyclohexa-1,4-diene **13** [9]. Enantioselective Sharpless dihydroxylation proceeded in good chemoselectivity but with modest yield and optical purity (25% ee). Unfortunately, all attempts to improve the enantioselectivity of this reaction failed. We discovered, however, that at a later stage the optical purity could be improved (see below). Diol protection gave dioxolane **14**, which underwent Saegusa oxidation to afford enone **15**. Subsequent α-iodination gave access to α-iodoenone **16**, which could be stereoselectively reduced under Corey–Itsuno conditions to yield allylic alcohol **17**. The use of a chiral oxazaborolidine catalyst led to kinetic resolution and increased the optical purity of **17** to 95% ee. Deprotection of the diol moiety followed by Stille cross coupling with vinyl stannane **18** finally gave **5** in excellent yield (Scheme 2).

**Scheme 3:**
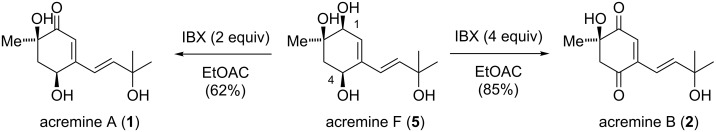
Synthesis of acremines A and B through selective oxidation of acremine F.

Since acremine F (**5**) can be expected to be the biogenetic precursor of acremines A (**1**) and B (**2**), we wanted to access these antifungal derivatives through selective oxidations. Indeed, treatment of **5** with IBX preferentially oxidized the C1-allylic alcohol, giving **1** in respectable yield. Prolonged treatment (9 h) of **5** with a large excess of IBX oxidized both secondary allylic alcohols and afforded **2** in good overall yield (Scheme 3).

**Scheme 4:**
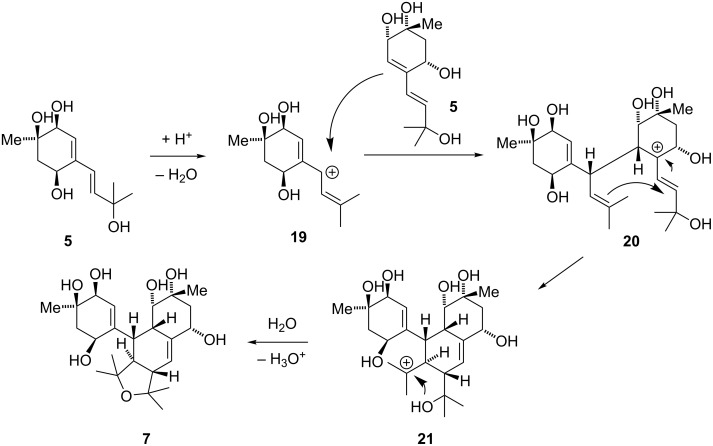
Proposed biomimetic dimerization of **5**.

Bisacremine E (**7**) was proposed to be formed in nature via [4 + 2] cycloaddition involving two acremine F (**5**) units [4]. Although dimerization of **5** through a Diels–Alder cycloaddition is not electronically favorable, we speculated that this reaction might proceed through ionic intermediates (Scheme 4).

To probe this hypothesis, we tried to generate allylic cation **19** by treatment with different acids or under thermal conditions (Table 1). Unfortunately, all conditions led to either decomposition of the starting material or elimination of the tertiary allylic alcohol to the unstable triene **6**. As we were able to observe elimination as well as acetate incorporation into the molecule the desired cation was clearly formed under a variety of conditions. Nevertheless, none of these could affect the desired cyclization. Attempts to enhance the stability of the molecule, and therefore prevent decomposition, by protection of the non-participating alcohols and attempts to generate the allylic cation from a cyclic ketal [10–12], aiming for a Gassman-type reaction mechanism, were also unfruitful.

**Table 1:** Representative screening conditions for the biomimetic cascade.

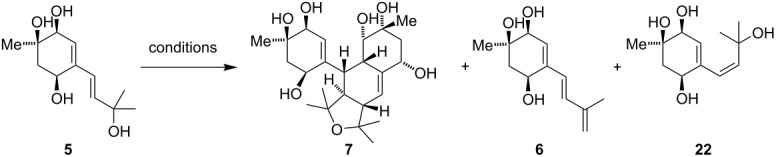				

entry	solvent	additive	temperature	observation

1	H_2_O	none	85 °C	**6**
2	H_2_O	none	100 °C	decomposition
3	PhMe	none	150 °C	**6**
4	*o*DCB	none	160 °C	decomposition
5	Et_2_O	4 M LiClO_4_	rt	**6**
6	neat	none	45 °C	**6**
7	neat	none	110 °C	decomposition
8	MeCN	12 kbar	rt	starting material
9	MeCN	AcOH, 12 kbar	60 °C	decomposition
10	MeCN	CSA	−40 °C to rt	**6**
11	neat	CSA	rt	decomposition
12	MeCN	AcOH	85 °C	**6** + acetate trapping
13	MeCN	HCOOH	90 °C	**6**
14	H_2_O	H_2_SO_4_	85 °C	decomposition
15	DMF	Mes-Acr-Ph, light^a^	rt	decomposition
16	PhMe	Ni(cod)_2_, PPh_3_	80 °C	starting material
17	MeCN	DCB, light^b^	rt	**22**
18	acetone	light^b^	rt	**22**

^a^Blue LED, ^b^medium pressure Hg lamp.

**Scheme 5:**
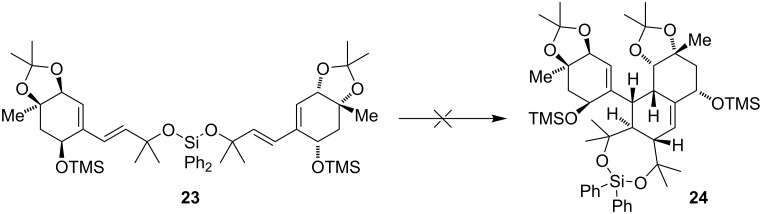
Attempted intramolecular cyclization of **23**.

While radical cations are known to undergo Diels–Alder reactions with electron-rich dienophiles [13–17], treatment of **5** with Fukuzumi’s catalyst [18] under illumination with blue light only led to decomposition of the starting material (Table 1, entry 15). Notably, a photoredox catalyst with a lower oxidation potential could not affect any reaction. Next, we tried to enhance the possibility for productive reactivity by tethering two units of **5** and therefore executed the reaction in an intramolecular way (Scheme 5). Unfortunately, treatment of **23** with various redox catalysts led either to decomposition or recovery of the starting material.

**Scheme 6:**
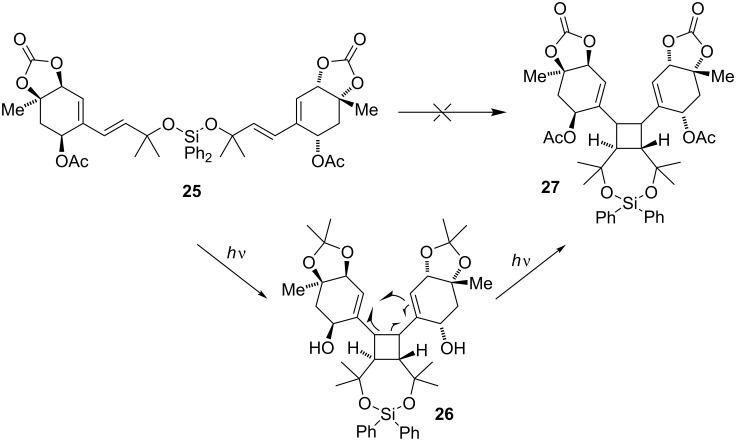
Attempted photochemical cyclization of **25**.

Next, we tried to trigger the cyclization through a [2 + 2] cycloaddition followed by vinyl cyclobutane rearrangement [19–20]. We reasoned that the initially formed divinyl cyclobutane [21] should undergo an allylic rearrangement to furnish the decalin system [22–23]. Condensation should then close the tetrahydrofuran ring of the natural product. Upon irradiation of **5** using a Hg lamp, however, the only productive pathway which could be observed was isomerization of the disubstituted double bond (Table 1, entries 17 and 18). Again, we attempted to promote the reaction by tethering two acremine units through a dioxysilane (Scheme 6) [24–26]. Unfortunately, irradiation of **25** with and without the presence of different photosensitizers only led to decomposition.

## Conclusion

In conclusion, we report the first asymmetric synthesis of acremine F (**5**) relying on the combination of a modestly enantioselective oxidation and a highly enantioselective reduction with kinetic resolution to access the acremine framework. The route proved to be scalable and delivered 300 mg of the natural product. Acremine F could further be converted into acremines A (**1**) and B (**2**) by a selective oxidation providing a versatile entry into this class of natural products. Furthermore, we investigated the proposed biomimetic dimerization of **5** to bisacremine E. Since these extensive studies were unsuccessful and no dimers could be observed under a variety of biomimetic conditions, it appears that this transformation requires enzymatic catalysis in nature.

## Supporting Information

Experimental procedures, spectroscopic data and copies of NMR spectra (PDF) as well as crystallographic data of compounds **16** and **17**. CIF files for **16** (CCDC 1854563) and **17** (CCDC 1854564) are available free from charge on https://www.ccdc.cam.ac.uk/structures/).

File 1 Experimental part.
