# Supplementary material for: Synthesis of acremines A, B and F and studies on the bisacremines
Source: Beilstein J Org Chem. 2019 Sep 23;15:2271–6. doi: 10.3762/bjoc.15.219 (PMC6774081; doi:10.3762/bjoc.15.219)
Supplement: File 1 — Experimental part. [file Beilstein_J_Org_Chem-15-2271-s001.pdf]

BEILSTEIN JOURNAL OF ORGANIC CHEMISTRY

Supporting Information

for

Synthesis of acremines A, B and F and studies on the bisacremines

Nils Winter and Dirk Trauner

Beilstein J. Org. Chem. **2019,** *15,* 2271–2276. doi:10.3762/bjoc.15.219

Experimental part

License and Terms: This is a supporting information file under the terms of the Creative Commons Attribution License (http://creativecommons.org/licenses/by/4.0). Please note that the reuse, redistribution and reproduction in particular requires that the authors and source are credited.
The license is subject to the *Beilstein Journal of Organic Chemistry* terms and conditions: (https://www.beilstein-journals.org/bjoc)

Table of contents

General experimental details **S3**

Experimental section **S5**

NMR spectra **S24**

Crystallographic data **S45**

References **S47**

General experimental details

All reactions were carried out with magnetic stirring, and, if moisture- or air-sensitive, under nitrogen or argon atmosphere using standard Schlenk techniques in oven-dried glassware (140 °C oven temperature). External bath temperatures were used to record all reaction temperatures. Low temperature reactions were carried out in a Dewar vessel filled with acetone/dry ice (–78 °C) or distilled water/ice (0 °C). High temperature reactions were conducted using a heated silicon oil bath in reaction vessels equipped with a reflux condenser or in a sealed flask. Tetrahydrofuran (THF) and diethyl ether (Et_2O) were distilled over sodium and benzophenone prior to use. Dichloromethane (CH_2Cl_2), triethylamine (Et_3N), diisopropylethylamine (DIPEA) and diisopropylamine (DIPA) were distilled over calcium hydride under a nitrogen atmosphere. All other solvents were purchased from Acros Organics as 'extra dry' reagents. All other reagents with a purity >95% were obtained from commercial sources (Sigma Aldrich, Acros, Alfa Aesar and others) and used without further purification.

Photochemical reactions were performed with a medium pressure mercury lamp (150 W) using a Heraeus power supply at room temperature.

Flash column chromatography was carried out with Merck silica gel 60 (0.040–0.063 mm). **Analytical thin-layer chromatography** (TLC) was carried out using Merck silica gel 60 F254 glass-backed plates and visualized under UV light at 254 nm. Staining was performed with ceric ammonium molybdate (CAM) or by oxidative staining with an aqueous basic potassium permanganate ($KMnO_4$) solution and subsequent heating.

NMR spectra (^1H NMR and ^{13}C NMR) were recorded in deuterated chloroform ($CDCl_3$) on a Bruker Avance III HD 400 MHz spectrometer equipped with a CryoProbe™, a Varian VXR400 S spectrometer, a Bruker AMX600 spectrometer or a Bruker Avance III HD 800 MHz spectrometer equipped with a CryoProbe™ and are reported as follows: chemical shift δ in ppm (multiplicity, coupling constant J in Hz, number of protons) for ^1H NMR spectra and chemical shift δ in ppm for ^{13}C NMR spectra. Multiplicities are abbreviated as follows: s = singlet, d = doublet, t = triplet, q = quartet, quint = quintet, br = broad, m = multiplet, or combinations thereof. Residual solvent peaks of $CDCl_3$ (δH = 7.26 ppm, δC = 77.16 ppm), d_4-MeOH (δH = 3.31 ppm, δC = 49.00 ppm) and d_6-acetone (δH = 2.05 ppm, δC = 29.84 ppm)

were used as an internal reference. NMR spectra were assigned using information ascertained from COSY, HMBC, HSQC and NOESY experiments.

High-resolution mass spectra (HRMS) were recorded on a Varian MAT CH7A or a Varian MAT 711 MS instrument by electron impact (EI) or electrospray ionization (ESI) techniques at the Department of Chemistry, Ludwig-Maximilians-University Munich.

Infrared spectra (IR) were recorded from 4000 cm^{-1} to 600 cm^{-1} on a PERKIN ELMER Spectrum BX II, FT-IR instrument. For detection, a SMITHS DETECTION DuraSampl*IR* II Diamond ATR sensor was used. Samples were prepared as a neat film or a thin powder layer. IR data in frequency of absorption (cm^{-1}) is reported as follows: w = weak, m = medium, s = strong, br = broad or combinations thereof.

All yields are isolated unless otherwise specified.

Experimental section

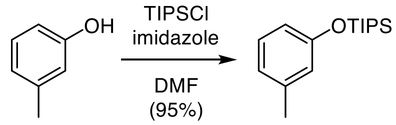

11

Silyl ether 11: TIPSCl (11 mL), 51.87 mmol) was added to a stirred solution of imidazole (7.10 g, 104 mmol) and *m*-cresol (6.25 mL, 59.70 mmol) in DMF (25 mL) and the reaction was stirred at room temperature for 20 h before it was poured into a mixture of Et_2O (50 mL), hexanes (50 mL) and 1 M aqueous H_2SO_4 (75 mL). The organic layer was separated and washed three times with 1 M aqueous NaOH and brine. Drying of the solvent over Na_2SO_4 and removal of the solvent under reduced pressure gave silyl ether **11** (13.1 g, 49.5 mmol, 95%) as a colorless oil.

Data for 11: ^{1}H-NMR (400 MHz, $CDCl_3$): δ (ppm) = 7.09 (t, J = 7.7 Hz, 1H), 6.77 – 6.64 (m, 3H), 2.30 (s, 3H), 1.32 – 1.19 (m, 3H), 1.10 (d, J = 7.3 Hz, 18H). **^{13}C-NMR (100 MHz, $CDCl_3$):** δ (ppm) = 156.1, 139.4, 129.1, 121.9, 120.8, 116.9, 21.6, 18.1, 12.8. **IR (ATR):** ν_{max} (cm^{-1}) = 2944 (m), 2893 (w), 2867 (m), 1304 (m), 1586 (m), 1487 (s), 1463 (m), 1384 (w), 1368 (w), 1280 (vs), 1165 (s), 1158 (s), 1071 (w), 1006 (m), 996 (w), 956 (s), 920 (w), 882 (s), 859 (w), 813 (s), 776 (s), 734 (w), 688 (vs), 680 (s), 660 (m). **HRMS (EI):** calc. for $C_{16}H_{28}OSi$ $[M]^+$: 264.1909, found: 264.1906.

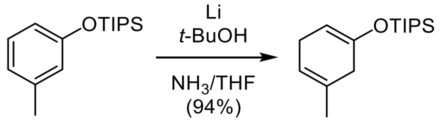

11 **13**

Silyl enol ether 13[1]: Li (4.34 g, 6.21 mmol) was added to a solution of THF (285 mL), *t*-BuOH (64 mL) and NH_3 (500 mL) at –78 °C and the mixture was stirred until all metal was dissolved. Silyl ether **11** (27.4 g, 104 mmol) was added slowly added and the reaction was stirred at –30 °C for 1 h. The solution was recooled to –78 °C and *t*-BuOH (30 mL) was added. After stirring for 30 min at –30 °C the solution was again cooled to –78 °C and NH_4Cl (32 g) was added portionwise until the blue colour disappeared. The mixture was slowly warmed to room temperature and the ammonia was evaporated under a slight stream of nitrogen. The reaction was diluted with H_2O and extracted with pentane. The organic phase

was dried over Na_2SO_4 and the solvent was removed under reduced pressure to give silyl enol ether **13** (26 g, 97.6 mmol, 94%) as a colorless oil.

Data for 13: ^{1}H-NMR (400 MHz, $CDCl_3$): δ (ppm) = 5.36 (tp, J = 3.2, 1.6 Hz, 1H), 4.85 (tq, J = 3.6, 1.3 Hz, 1H), 2.72 (tdq, J = 6.3, 3.1, 1.7 Hz, 2H), 2.64 – 2.55 (m, 2H), 1.69 (s, 3H), 1.19 – 1.12 (m, 3H), 1.10 – 1.07 (m, 19H). **^{13}C-NMR (100 MHz, $CDCl_3$):** δ (ppm) = 148.2, 131.1, 118.6, 100.1, 35.5, 27.4, 23.1, 18.2, 12.8. **IR (ATR):** ν_{max} (cm^{-1}) = 3040 (w), 2944 (w), 2891 (w), 2866 (m), 2822 (w), 1699 (w), 1667 (w), 1463 (w), 1384 (w), 1346 (w), 1240 (w), 1217 (s), 1137 (s), 1071 (w), 1014 (w), 996 (w), 960 (w), 936 (w), 927 (w), 882 (s), 822 (w), 776 (m), 682 (m). **HRMS (EI):** calc. for $C_{16}H_{30}OSi$ $[M]^+$: 266.2066, found: 266.2061.

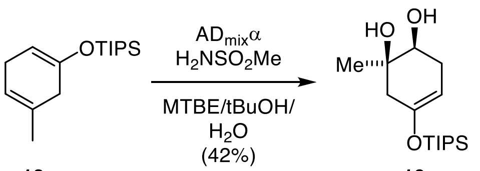

13 10

Diol 10[1]: Methansulfonamide (2.36 g, 24.84 mmol) was added to a solution of $AD_{mix}\alpha$ (50.0 g) in *t*-BuOH (93 mL), MTBE (47 mL) and H_2O (142 mL) and the resulting solution was stirred for 15 min at room temperature before it was cooled to –8 °C. Silyl enol ether **13** (9.37 g, 35.17 mmol) was added and the reaction was stirred at–8 °C for 7 d, diluted with H_2O and extracted with EtOAc. The combined organic phases were sequentially washed with 10% aqueous $Na_2S_2O_3$, saturated $NaHCO_3$ and brine and dried over $MgSO_4$. Removal of the solvent under reduced pressure and purification of the resultant residue by flash column chromatography (EtOAc:hexanes 1:1, R_f = 0.4) gave diol **10** (4.42 g, 14.71 mmol, 42%, 77% brsm, 25% ee) as a colourless oil.

Data for 10: ^{1}H-NMR (400 MHz, $CDCl_3$): δ (ppm) = 4.72 – 4.62 (m, 1H), 3.56 (t, J = 5.0 Hz, 1H), 2.39 – 2.12 (m, 6H), 1.22 (s, 3H), 1.16 – 1.09 (m, 3H), 1.08 – 1.01 (m, 18H). **^{13}C-NMR (100 MHz, $CDCl_3$):** δ (ppm) = 148.2, 98.2, 72.4, 72.0, 41.7, 29.7, 25.0, 18.1, 12.7. **IR (ATR):** ν_{max} (cm^{-1}) = 3409 (m, br), 2946 (m), 2867 (m), 2361 (w), 1715 (m), 1659 (s), 1464 (w), 1377 (w), 1325 (w), 1257 (m), 1201 (m), 1165 (w), 1060 (m), 968 (w), 950 (w), 884 (m), 685 (w). **HRMS (EI):** calc. for $C_{16}H_{32}O_3Si$ $[M]^+$: 300.2121, found: 300.2119. $[\alpha]_D^{20}$ = +1.6 (c = 0.06, $CHCl_3$)

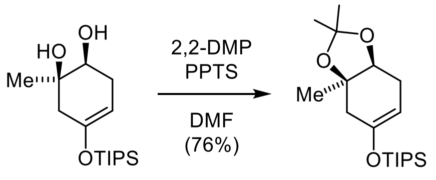

10 **14**

Acetonide 14[1]**:** PPTS (307 mg, 1.21 mmol) was added to a stirred solution of diol **10** (7.30 g, 24.3 mmol) and 2,2-DMP (29.2 mL) in DMF (245 mL) and the solution was stirred for 2 h at room temperature before it was quenched with NEt_3 (18.2 mL). The reaction was diluted with aqueous $NaHCO_3$ and extracted with Et_2O. The combined organic phases were washed with brine and dried over Na_2SO_4. Removal of the solvent under reduced pressure and purification by flash column chromatography (EtOAc:hexanes 5:95, R_f = 0.4) gave acetonide **14** (6.28 g, 18.4 mol, 76%) as a colourless oil.

Data for 14: ^{1}H-NMR (400 MHz, $CDCl_3$): δ (ppm) = 4.67 (dd, J = 2.2, 1.2 Hz, 1H), 3.93 (dt, J = 3.4, 1.6 Hz, 1H), 2.46 – 2.33 (m, 1H), 2.14 (dddd, J = 14.4, 5.4, 3.6, 1.6 Hz, 1H), 1.84 (ddd, J = 16.6, 6.0, 1.5 Hz, 1H), 1.79 – 1.67 (m, 1H), 1.37 (d, J = 3.5 Hz, 6H), 1.32 (s, 3H), 1.21 – 1.11 (m, 3H), 1.10 – 1.05 (m, 18H). **^{13}C-NMR (100 MHz, $CDCl_3$):** δ (ppm) = 151.2, 107.6, 107.2, 80.1, 77.3, 27.9, 27.7, 26.6, 24.4, 24.3, 18.2, 18.1, 12.7. **IR (ATR):** ν_{max} (cm^{-1}) = 2943 (m), 2893 (w), 2867 (m), 2361 (w), 1663 (m), 1464 (w), 1374 (w), 1326 (w), 1297 (w), 1239 (w), 1208 (s), 1191 (w), 1167 (w), 1132 (w), 1086 (s), 1066 (w), 1006 (m), 991 (m), 956 (w), 908 (w), 883 (s), 860 (w), 826 (w), 815 (w), 784 (w), 757 (w), 682 (m), 665 (w). **HRMS (EI):** calc. for $C_{16}H_{32}O_3Si$ [M]$^+$: 340.2434, found: 340.2424. $[\alpha]_D^{20}$ = +24 ° (c = 0.01, $CHCl_3$).

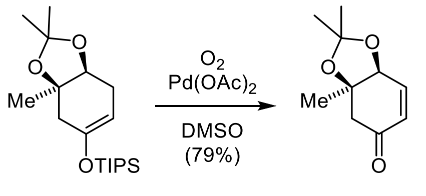

14 **15**

Ketone 15[1]**:** $Pd(OAc)_2$ (4.14 g, 18.44 mmol) was added to a solution of acetonide **14** (6.28 g, 18.44 mmol) in DMSO (13.7 mL). The reaction was set under vacuum and backfilled with O_2 for three cycles and stirred at room temperature overnight before it was submitted to flash column chromatography (EtOAc:hexanes 15:85, R_f = 0.2) to give ketone **15** (2.67 g, 14.65 mmol, 79%) as a colorless oil.

Data for 15: ^{1}H-NMR (400 MHz, CDCl$_3$): δ (ppm) = 6.75 (dd, J = 10.2, 3.7 Hz, 1H), 6.08 (dd, J = 10.2, 1.1 Hz, 1H), 4.41 (dd, J = 3.8, 1.1 Hz, 1H), 2.84 (d, J = 16.2 Hz, 1H), 2.49 (d, J = 16.3 Hz, 1H), 1.42 (s, 3H), 1.38 (s, 3H), 1.35 (s, 3H). **^{13}C-NMR (100 MHz, CDCl$_3$):** δ (ppm) = 197.1, 143.9, 130.7, 110.3, 80.7, 76.1, 47.5, 28.5, 28.4, 26.1. **IR (ATR):** ν_{max} (cm^{-1}) = 3362 (w, br), 2985 (w), 2936 (w), 1681 (s), 1459 (w), 1380 (m), 1312 (w), 1270 (w), 1233 (s), 1212 (m), 1180 (w), 1160 (w), 1144 (w), 1109 (w), 1082 (m), 1046 (s), 1020 (m), 988 (w), 956 (w), 944 (w), 894 (w), 866 (w), 830 (m), 807 (w), 772 (w), 705 (w), 668 (w). **HRMS (EI):** calc. for $C_{10}H_{14}O_3$ [M]$^+$: 182.0943, found: 182.0935. $[\alpha]_D^{20}$ = +12 ° (c = 0.01, CHCl$_3$).

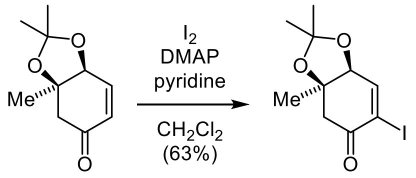

15 16

Vinyl iodide 16: A solution of iodine (11.18 g, 44.01 mmol) in CH_2Cl_2 (45 mL) and pyridine (11.83 mL) was added slowly to a solution of ketone **15** (2.67 g, 14.65 mmol) and DMAP (1.80 g, 14.65 mmol) in CH_2Cl_2 (100 mL) and the resulting solution was stirred for 36 h at room temperature in the dark before it was diluted with CH_2Cl_2 and washed with a 20% aqueous solution of $Na_2S_2O_3$, 1 M aqueous HCl and brine. The organic phase was dried over Na_2SO_4 and the solvent was removed under reduced pressure. Purification of the resulting residue by flash column chromatography (EtOAc:hexanes 1:5, R_f = 0.3) gave vinyl iodide **16** (2.83 g, 9.18 mmol, 63%, 73% brsm) as a colorless oil).

Data for 16: ^{1}H-NMR (400 MHz, CDCl$_3$): δ (ppm) = 7.51 (d, J = 3.9 Hz, 1H), 4.41 (d, J = 3.8 Hz, 1H), 3.12 (d, J = 16.1 Hz, 1H), 2.70 (d, J = 16.1 Hz, 1H), 1.46 (d, J = 0.8 Hz, 3H), 1.43 (s, 3H), 1.38 – 1.35 (m, 3H). **^{13}C-NMR (100 MHz, CDCl$_3$):** δ (ppm) = 190.1, 152.9, 110.9, 106.5, 81.2, 78.6, 45.9, 28.7, 28.5, 26.1. **IR (ATR):** ν_{max} (cm^{-1}) = 2987 (w), 2935 (w), 1693 (s), 1599 (w), 1459 (w), 1377 (m), 1327 (w), 1296 (w), 1269 (w), 1235 (s), 1215 (s), 1181 (m), 1145 (w), 1113 (w), 1092 (m), 1053 (m), 1035 (m), 986 (w), 949 (w), 924 (w), 892 (w), 842 (w), 781 (w), 729 (w). **HRMS (EI):** calc. for $C_{10}H_{13}IO_3$ [M]$^+$: 307.9909, found: 307.9908. $[\alpha]_D^{20}$ = –17 ° (c = 0.05, CHCl$_3$).

The enantiomeric excess was determined using chiral HPLC (chiralpak Od-h) *i*-PrOH:*n*-heptane 0.5:99.5-2:98 (over 30 min, 1 mL/min).

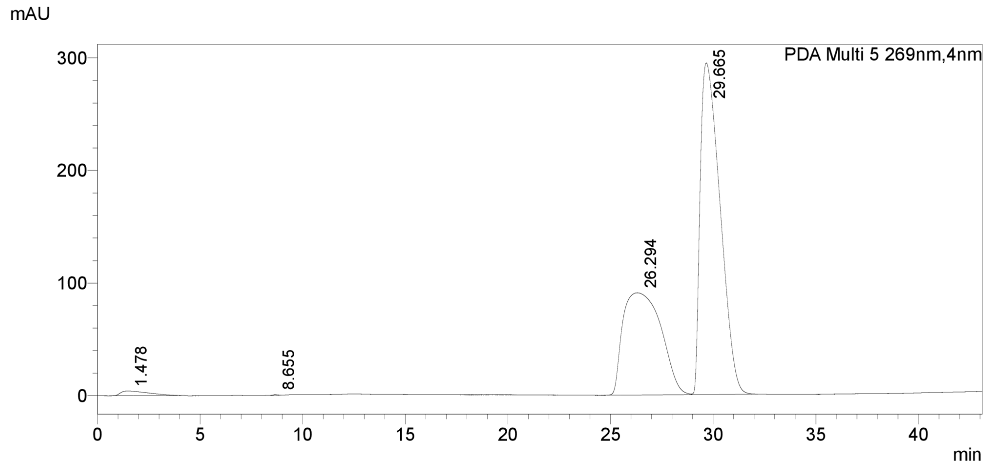

<Peak Table>

Detector A Channel 1

Peak#	Ret. Time	Area	Height	Conc.	Unit	Mark	Name
Total							

PDA Ch5 269nm

Peak#	Ret. Time	Area	Height	Conc.	Unit	Mark	Name
1	1.478	385248	4169	1.202			
2	8.655	8423	536	0.026		V	
3	26.294	11795277	90793	36.811			
4	29.665	19854158	294541	61.961		V	
Total		32043107	390039				

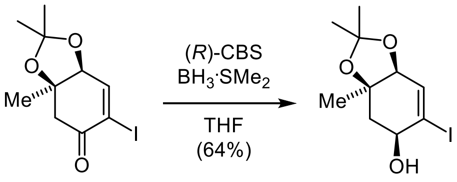

16 17

Allylic alcohol 17: $BH_3{\cdot}SMe_2$ (0.87 mL, 9.18 mmol) was added dropwise to an ice-cooled solution of (*R*)-CBS catalyst (1.27 g, 4.58 mmol) in THF (126 mL) and the solution was stirred for 15 min at that temperature before a solution of vinyl iodide **16** (2.83 g, 9.18 mmol) in THF (35 mL) was slowly added. The reaction was stirred at 0 °C for 2 h before it was quenched with MeOH and poured into brine and extracted with EtOAc. The combined organic layers were dried over Na_2SO_4 and the solvent was removed under reduced pressure. Purification of the resultant residue by flash column chromatography gave allylic alcohol **17** (1.81 g, 5.84 mmol, 64%, 95% ee) as a colorless oil.

Data for 17: ^{1}H-NMR (400 MHz, $CDCl_3$): δ (ppm) = 6.43 (d, J = 3.6 Hz, 1H), 4.17 (dd, J = 3.6, 0.8 Hz, 1H), 4.12 (dt, J = 9.2, 4.3 Hz, 1H), 3.32 (d, J = 10.0 Hz, 1H), 2.36 (dd, J = 14.6, 3.8 Hz, 1H), 2.10 (dd, J = 14.6, 4.7 Hz, 1H), 1.45 (s, 6H), 1.37 (s, 3H). **^{13}C-NMR (100 MHz, $CDCl_3$):** δ (ppm) = 137.1, 110.7, 107.4, 79.4, 79.1, 72.7, 39.7, 29.2, 28.9, 26.3. **IR (ATR):**

ν_{max} (cm^{-1}) = **HRMS (EI):** calc. for $C_{10}H_{15}IO_3I$ [*M*]$^+$: 310.0066, found: 310.0056. $[\alpha]_D^{20}$ = –105.9 (c = 0.03, $CHCl_3$)

The enantiomeric excess was determined using chiral HPLC (chiralpak Od-h) *i*-PrOH:*n*-heptane 0.5:99.5-2:98 (over 30 min, 1 mL/min).

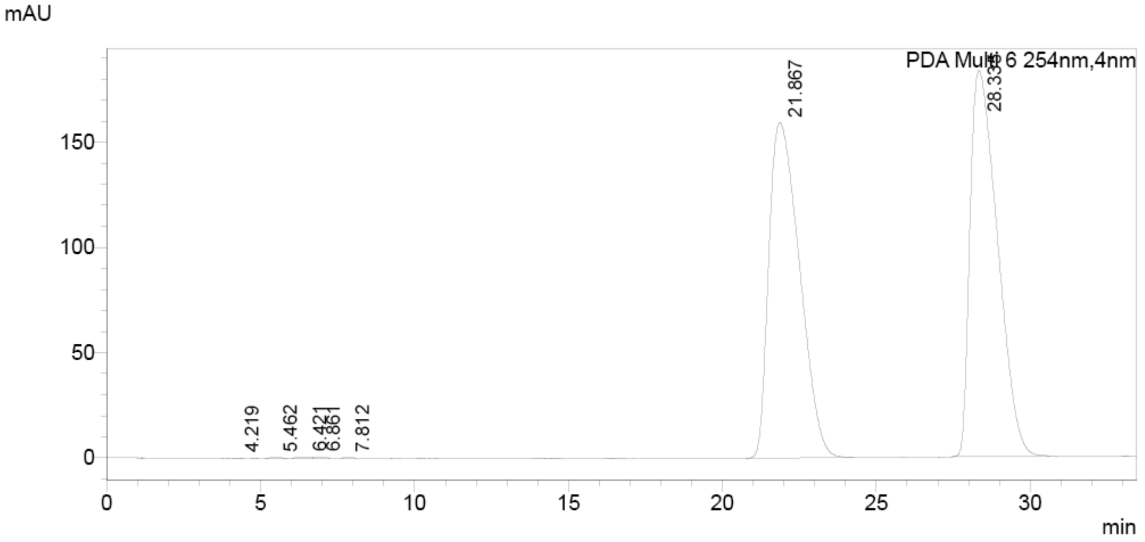

PDA Ch6 254nm

Peak#	Ret. Time	Area	Height	Conc.	Unit	Mark	Name
1	4.219	1224	124	0.000			
2	5.462	9684	475	0.000			
3	6.421	15975	607	0.000			
4	6.861	12144	628	0.000		V	
5	7.812	4480	302	0.000			
6	21.867	11051529	159434	0.000			
7	28.335	11362332	183375	0.000			
Total		22457368	344946				

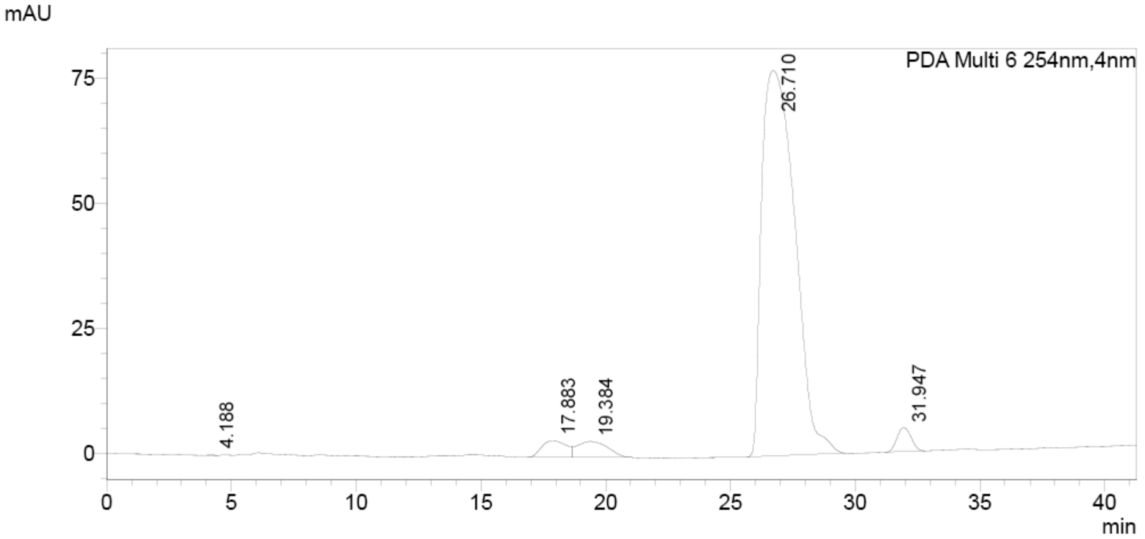

PDA Ch6 254nm

Peak#	Ret. Time	Area	Height	Conc.	Unit	Mark	Name
1	4.188	2991	254	0.000			
2	17.883	236756	3385	0.000			
3	19.384	278676	3243	0.000		V	
4	26.710	7118383	77141	0.000			
5	31.947	187958	4785	0.000			
Total		7824765	88808				

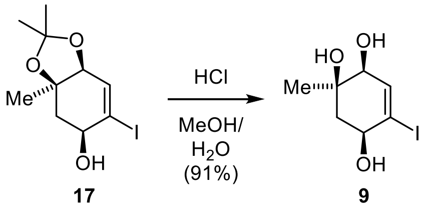

Triol 9: Aqueous 1 M HCl (3.7 mL) was added to a stirred solution of vinyl iodide **17** (575 mg, 1.85 mmol) in MeOH (7.4 mL) and the solution was stirred overnight at room temperature in the dark. The reaction mixture was directly submitted to flash column chromatography (EtOAc) to give triol **9** (455 mg, 1.68 mmol, 91%) as a colorless oil.

Data for 9: ^{1}H-NMR (400 MHz, d$_4$-MeOH): δ (ppm) = 6.40 (dd, J = 3.7, 0.8 Hz, 1H), 4.04 (tt, J = 5.4, 1.0 Hz, 1H), 3.73 (dt, J = 3.7, 0.9 Hz, 1H), 2.05 (dd, J = 13.6, 5.3 Hz, 1H), 1.97 (ddd, J = 13.6, 5.3, 0.7 Hz, 1H), 1.20 (s, 3H). **^{13}C-NMR (100 MHz, d$_4$-MeOH):** δ (ppm) = 141.5, 107.2, 74.8, 73.5, 70.9, 41.8, 25.5. **IR (ATR):** ν_{max} (cm^{-1}) = 3270 (s), 2971 (w), 2922 (w), 2863 (w), 1706 (w), 1625 (w), 12449 (w), 1431 (w), 1696 (w), 1374 (w), 1329 (w), 1284 (w), 1253 (m), 1142 (s), 1108 (m), 1066 (s), 1053 (vs), 1036 (s), 1000 (s), 934 (s), 908 (m), 869 (m), 833 (m), 742 (w). **HRMS (ESI):** calc. for $C_7H_{10}IO_3$ [M–H]$^-$: 268.9680, found: 298.9684. $[\alpha]_D^{20}$ = +14.0 ° (c = 0.0043, MeOH).

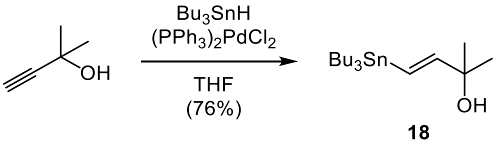

Vinylstannane 18: Bu_3SnH (1.58 mL, 5.96 mmol) was added dropwise to a suspension of 2-methyl-3-butyn-2-ol (0.58 mL, 5.94 mmol) and $(PPh_3)_2PdCl_2$ (83 mg, 0.119 mmol) in THF (17 mL) and the resultant solution was stirred for 1 h at room temperature before it was concentrated under reduced pressure. The resultant residue was purified by flash column chromatography (EtOAc:hexanes 8:92, R_f = 0.3) to yield vinyl stannane **18** (1.70 g, 4.53 mmol, 76%) as a colorless oil.

Data for 18: ^{1}H-NMR (400 MHz, CDCl$_3$): δ (ppm) = 6.19 – 5.97 (m, 2H), 1.71 (s, 1H), 1.54 – 1.41 (m, 6H), 1.33 – 1.23 (m, 5H), 0.86 (td, J = 7.7, 1.6 Hz, 15H). **^{13}C-NMR (100 MHz, CDCl$_3$):** δ (ppm) = 155.7, 122.4, 72.5, 29.5, 29.2, 27.3, 13.8, 9.5. **IR (ATR):** ν_{max} (cm^{-1}) = 3357 (w, br), 2956 (s), 2923 (s), 2871 (w), 2853 (w), 1600 (w), 1521 (w), 1463 (w), 1417 (w), 1374 (m), 1292 (w), 1201 (w), 1147 (w), 1071 (w), 989 (m), 960 (w), 904 (w), 874 (w), 781 (w), 667 (m). **HRMS (EI):** calc. for $C_{17}H_{36}OSn$ [M]$^+$: 376.1788, found: 376.1789

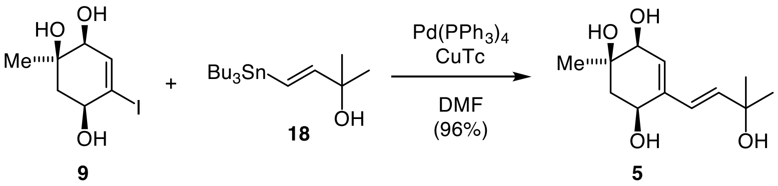

Acremine F (5): A solution of triol **9** (430 mg, 1.59 mmol), vinylstannane **18** (1.20 g, 3.19 mmol), Pd(PPh$_3$)$_4$ (177 mg, 0.153 mmol) and CuTc (381 mg, 2.00 mmol) in DMF (37 mL) was stirred at 65 °C overnight in the dark. The reaction was cooled to room temperature, diluted with MeOH and filtered through a pad of celite. Evaporation of the solvent under reduced pressure and purification of the resulting residue by flash column chromatography (EtOAc, R_f = 0.25) gave acremine F (**5**) (350 mg, 1.53 mmol, 96%) as a colorless oil.

Data for 5: ^{1}H-NMR (400 MHz, d$_4$-MeOH): δ (ppm) = 6.19 (d, J = 16.1 Hz, 1H), 6.08 (d, J = 16.1 Hz, 1H), 5.63 (d, J = 2.8 Hz, 1H), 4.35 (t, J = 3.9 Hz, 1H), 3.95 (d, J = 2.8 Hz, 1H), 2.09 (dd, J = 14.3, 3.3 Hz, 1H), 1.85 (dd, J = 14.3, 4.5 Hz, 1H), 1.33 (s, 3H), 1.32 (s, 3H), 1.27 (s, 3H). **^{13}C-NMR (100 MHz, d$_4$-MeOH):** δ (ppm) = 139.4, 139.2, 130.8, 127.1, 73.8, 72.0, 71.4, 65.6, 42.0, 30.0, 29.9, 26.5. **IR (ATR):** ν_{max} (cm^{-1}) = 3372 (br), 2971 (s), 2927 (m), 2360 (m), 2168 (w), 2012 (w), 1622 (w), 1373 (m), 1264 (w), 1150 (s), 1072 (m), 1038 (s), 999 (m), 971 (m), 883 (w), 832 (w), 658 (w). **HRMS (ESI):** calc for $C_{12}H_{19}O_4$ [M–H]$^-$: 227.1289, found: 227.1292 $[\alpha]_D^{20}$ = –46.6 ° (c = 0.009, CHCl$_3$).

^{1}H-NMR (400 MHz, d$_6$-Acetone): δ (ppm) = 6.17 (d, J = 16.1 Hz, 1H), 6.09 (d, J = 16.0 Hz, 1H), 5.51 (d, J = 2.3 Hz, 1H), 4.32 – 4.25 (m, 1H), 4.19 (d, J = 8.6 Hz, 1H), 4.06 (t, J = 1.3 Hz, 1H), 3.99 – 3.94 (m, 1H), 3.92 (d, J = 7.9 Hz, 1H), 3.60 (s, 1H), 2.12 – 2.04 (m, 2H), 1.81 (ddd, J = 14.4, 4.3, 1.1 Hz, 1H), 1.27 (s, 6H), 1.24 (s, 3H). **^{13}C-NMR (100 MHz, d$_6$-Acetone):** δ (ppm) = 139.6, 139.0, 130.4, 126.6, 73.2, 71.6, 70.4, 64.8, 41.8, 30.48, 30.45, 27.1.

Table 1: ^{1}H- and ^{13}C-NMR chemical shifts in d_6-acetone of natural[2] *vs*. synthetic acremine F (**5**).

No.	**^{1}H-NMR Isolated 500 MHz, d6-acetone [ppm]**	**^{1}H-NMR Synthetic 400 MHz, d6-acetone [ppm]**	**^{13}C-NMR Isolated 125 MHz, d6-acetone [ppm]**	**^{13}C-NMR Synthetic 100 MHz, d6-acetone [ppm]**
1	3.95 (d, J = 2.57 Hz, 1H)	3.99 – 3.94 (m, 1H)		73.2
2	5.52 (d, J = 2.57 Hz, 1H)	5.51 (d, J = 2.3 Hz, 1H)		130.4
3				139.0
4	4.24–4.31 (m, 1H)	4.32 – 4.25 (m, 1H)		64.8
5a	2.02–2.08 (m, 1H)	2.12 – 2.04 (m, 1H)		40.6
5b	1.81 (dd, J = 14.34, 4.41 Hz, 1H)	1.81 (ddd, J = 14.4, 4.3, 1.1 Hz, 1H)		
6				71.6
7a	6.09 (d, J = 16.18 Hz, 1H)	6.09 (d, J = 16.0 Hz, 1H)		139.6
8	6.17 (d, J = 16.18 Hz, 1H)	6.17 (d, J = 16.1 Hz, 1H)		126.6
9				70.4
10	1.28 (s, 3H)	1.27 (s, 3H)		30.45
11a	1.28 (s, 3H)	1.27 (s, 3H)		30.48
12	1.24 (s, 3H)	1.24 (s, 3H)		27.1
1-OH		4.19 (d, J = 8.6 Hz, 1H)		
4-OH		4.06 (t, J = 1.3 Hz, 1H)		
6-OH		3.92 (d, J = 7.9 Hz, 1H)		
9-OH		3.60 (s, 1H)		

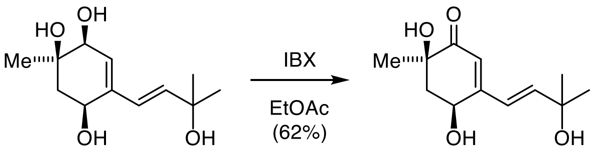

5 **1**

Acremine A (1): A suspension of acremine F (**5**) (50 mg, 0.186 mmol) and IBX (104 mg, 0.372 mmol) was vigorously stirred at 80 °C for 3 h. After cooling to room temperature, the solids were removed by filtration and the solvent was evaporated under reduced pressure. Purification by flash column chromatography (EtOAc, R_f = 0.25) gave acremine A (**1**) (20 mg, 0.115 mmol, 62%) as a colourless solid.

Data for 1: **^{1}H-NMR (400 MHz, $CDCl_3$):** δ (ppm) = 6.59 (d, J = 16.0 Hz, 1H), 6.38 (d, J = 16.1 Hz, 1H), 6.00 (s, 1H), 4.61 (t, J = 5.7 Hz, 1H), 3.70 (s, 1H), 3.44 (s, 1H), 2.36 (dd, J = 13.7, 4.8 Hz, 1H), 2.22 (dd, J = 13.7, 6.6 Hz, 1H), 1.38 (s, 6H), 1.34 (s, 3H). **^{13}C-NMR (100 MHz, $CDCl_3$):** δ (ppm) = 200.3, 158.1, 147.1, 124.2, 123.0, 73.1, 71.4, 65.5, 43.8, 29.8, 29.6, 24.8. **IR (ATR):** ν_{max} (cm^{-1}) = 3396 (br), 2971 (s), 1666 (vs), 1634 (s), 1592 (m), 1374 (m), 1253 (m), 1205 (m), 1129 (s), 1063 (m), 1036 (w), 980 (m), 902 (w), 850 (w). **HRMS (EI):** calc for $C_{12}H_{18}O_4$ $[M{-}CH_3]^+$: 226.1200, found: 226.1191. $[\alpha]_D^{20}$ = +6.0 ° (c = 0.017, $CHCl_3$).

Table 2: ^{1}H- and ^{13}C-NMR chemical shifts in $CDCl_3$ of natural[3] vs. synthetic Acremine A (**1**).

**No.**	**^{1}H-NMR Isolated 500 MHz, $CDCl_3$ [ppm]**	**^{1}H-NMR Synthetic 400 MHz, $CDCl_3$ [ppm]**	**^{13}C-NMR Isolated 125 MHz, $CDCl_3$ [ppm]**	**^{13}C-NMR Synthetic 100 MHz, $CDCl_3$ [ppm]**
1			200.1	200.3
2	6.00 (brs, 1H)	6.00 (s, 1H)	123.1	123.0
3			157.9	158.1
4	4.63 (m, 1H)	4.61 (t, J = 5.7 Hz, 1H)	65.6	65.5
5a	2.36 (dd, J = 13.0, 5.1 Hz, 1H)	2.36 (dd, J = 13.7, 4.8 Hz, 1H)	43.9	43.8
5b	2.10 (dd, J = 13.0, 8.8 Hz, 1H)	2.22 (dd, J = 13.7, 6.6 Hz, 1H)		
6			73.1	73.1
7	6.44 (d, J = 16.0 Hz, 1H)	6.38 (d, J = 16.1 Hz, 1H)	124.3	124.2

8	6.67 (d, J = 16.0 Hz, 1H)	6.59 (d, J = 16.0 Hz, 1H)	147.1	147.1
9			71.4	71.4
10	1.35 (s, 3H)	1.38 (s, 3H)	29.8	29.8
11	1.35 (s, 3H)	1.38 (s, 3H)	29.8	29.7
12	1.30 (s, 3H)	1.34 (s, 3H)	24.1	24.8
1-OH				
4-OH	2.96 (d, J = 6.5 Hz, 1H)	3.70 (s, 1H)		
6-OH	3.41 (s, 1H)	3.44 (s, 1H)		

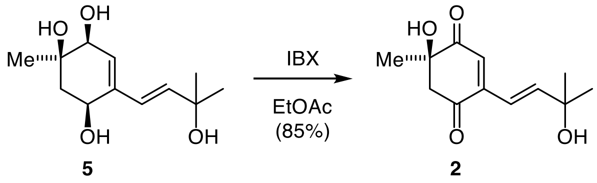

Acremine B (2): A suspension of acremine F (**5**) (30 mg, 0.131 mmol) and IBX (149 mg, 0.526 mmol) was vigorously stirred at 80 °C for 9 h. After cooling to room temperature, the solids were removed by filtration and the solvent was evaporated under reduced pressure. Purification by flash column chromatography (EtOAc:hexanes 1:1, R_f = 0.25) gave acremine B (**2**) (25 mg, 0.111 mmol, 85%) as a colourless solid.

Data for 2: ^{1}H-NMR (400 MHz, $CDCl_3$): δ (ppm) = 6.80 (d, J = 16.1 Hz, 1H), 6.75 (s, 1H), 6.53 (dd, J = 16.0, 0.7 Hz, 1H), 3.67 (s, 1H), 3.19 – 2.99 (m, 2H), 1.66 (s, 1H), 1.43 (d, J = 0.9 Hz, 3H), 1.40 (s, 3H), 1.39 (s, 3H). **^{13}C-NMR (100 MHz, $CDCl_3$):** δ (ppm) = 201.8, 195.6, 149.5, 147.9, 130.0, 118.5, 75.3, 71.6, 53.2, 29.7, 27.8. **IR (ATR):** ν_{max} (cm^{-1}) = 3380 (br), 2975 (m), 2931 (w), 1681 (vs), 1634 (m), 159 (m), 1459 (w), 1373 (m), 1254 (m), 1129 (s), 1053 (w), 974 (m), 906 (w), 851 (w), 824 (w), 780 (w), 754 (w), 668 (w). **HRMS (EI):** calc for $C_{11}H_{13}O_4$ [$M–CH_3$]$^+$: 209.0808, found: 209.0824. $[\alpha]_D^{20}$ = –64.9 ° (c = 0.021, $CHCl_3$).

Table 3: ^{1}H- and ^{13}C-NMR chemical shifts in $CDCl_3$ of natural[2] vs. synthetic Acremine B (**2**).

**No.**	**^{1}H-NMR Isolated 500 MHz, $CDCl_3$ [ppm]**	**^{1}H-NMR Synthetic 400 MHz, $CDCl_3$ [ppm]**	**^{13}C-NMR Isolated 125 MHz, $CDCl_3$ [ppm]**	**^{13}C-NMR Synthetic 100 MHz, $CDCl_3$ [ppm]**
1			202	201.8
2	6.74 (s, 1H)	6.75 (s, 1H)	129.8	130.0
3			148	147.9
4			195.4	195.6
5a	3.03 (d, J = 15.16 Hz, 1H)	3.19 – 2.99 (m, 2H)	53.1	53.2
5b	3.11 (d, J = 15.16 Hz, 1H)	3.19 – 2.99 (m, 2H)		
6			75.1	75.3
7	6.50 (d, J = 15.96 Hz, 1H)	6.53 (dd, *J* = 16.0, 0.7 Hz, 1H)	118.3	118.5
8	6.80 (d, J = 15.96 Hz, 1H)	6.80 (d, *J* = 16.1 Hz, 1H)	149.4	149.5
9			71.4	71.6
10	1.37 (s, 3H)	1.40 (s, 3H)	29.5	29.7
11a	1.37 (s, 3H)	1.39 (s, 3H)	29.5	29.7
12	1.38 (s, 3H)	1.43 (d, *J* = 0.9 Hz, 3H)	27.6	27.8
4-OH		3.67 (s, 1H)		
9-OH		1.66 (s, 1H)		

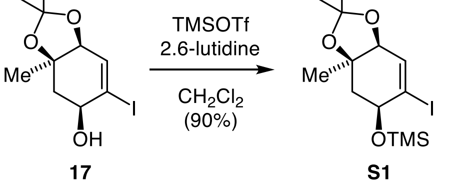

17 **S1**

Silyl ether S1: 2,6-lutidine (0.20 mL, 1.72 mmol) and TMSOTf (0.16 mL, 0.861 mmol) were sequentially added to an ice-cooled solution of vinyl iodide **17** (107 mg, 0.345 mmol) and the reaction was stirred for 90 min at that temperature before it was quenched with saturated

aqueous NH_4Cl and extracted with CH_2Cl_2. The combined organic layers were dried over Na_2SO_4 and concentrated under reduced pressure. Purification of the resulting residue by flash column chromatography (EtOAc:hexanes 4:96) gave silyl ether **S1** (118 mg, 0.309 mmol, 90%) as a colourless oil.

Data for S1: ^{1}H-NMR (400 MHz, $CDCl_3$): δ (ppm) = 6.57 (dd, J = 4.9, 1.8 Hz, 1H), 4.04 – 3.97 (m, 1H), 3.90 (dt, J = 4.9, 1.3 Hz, 1H), 2.07 – 1.89 (m, 2H), 1.46 (d, J = 0.7 Hz, 3H), 1.37 (d, J = 0.7 Hz, 3H), 1.32 (d, J = 0.8 Hz, 3H), 0.21 (s, 9H). **^{13}C-NMR (100 MHz, $CDCl_3$):** δ (ppm) = 133.7, 115.6, 110.0, 78.5, 78.4, 71.2, 42.7, 28.7, 27.3, 23.8, 0.6. IR (ATR): 2963 (w), 2858 (w), 1627 (w), 1458 (w), 1378 (m), 1368 (m), 1251 (s), 1217 (s), 1183 (m), 1123 (m), 1094 (s), 1073 (s), 1032 (m), 992 (w), 949 (w), 916 (w), 887 (vs), 841 (vs), 803 (w), 749 (w). **IR (ATR)** ν_{max} (cm^{-1}) = **HRMS (EI):** calc. for $C_{12}H_{20}O_3ISi$ [*M–CH3*]$^+$: 367.0221, found: 367.0225. $[\alpha]_D^{20}$ = +109 ° (c = 0.017, $CHCl_3$).

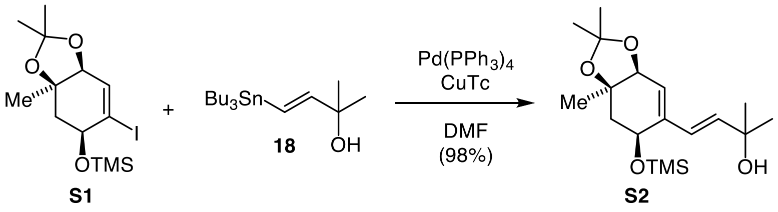

Diene S2: A mixture of silyl ether **S1** (178 mg, 0.466 mmol), vinylstanine **18** (349 mg, 0.931 mmol), $Pd(PPh_3)_4$ (51 mg, 0.044 mmol), and CuTc (108 mg, 0.566 mmol) in DMF (11 mL) was stirred at 65 °C overnight in the dark. The reaction was diluted with brine and extracted with EtOAc. The combined organic phases were dried over Na_2SO_4 and concentrated under reduced pressure. Purification of the resulting residue by flash column chromatography (EtOAc:hexanes 20:80, R_f = 0.2) gave diene **S2** (156 mg, 0.458 mmol, 98%) as a colourless oil.

Data for S2: ^{1}H-NMR (400 MHz, $CDCl_3$): δ (ppm) = 6.29 – 6.09 (m, 2H), 5.86 (ddd, J = 4.7, 1.6, 0.8 Hz, 1H), 4.27 (ddt, J = 10.7, 5.8, 1.5 Hz, 1H), 4.12 (dd, J = 4.8, 1.2 Hz, 1H), 1.94 (dd, J = 5.9, 1.0 Hz, 1H), 1.65 – 1.61 (m, 1H), 1.48 – 1.44 (m, 3H), 1.39 – 1.38 (m, 3H), 1.35 (d, J = 1.4 Hz, 6H), 1.29 – 1.28 (m, 3H), 0.17 (s, 9H). **^{13}C-NMR (100 MHz, $CDCl_3$):** δ (ppm) = 143.94, 139.8, 124.4, 119.9, 109.2, 78.3, 71.1, 68.6, 42.6, 29.9, 29.9, 28.8, 28.0, 27.3, 27.0, 23.7, 17.7, 13.8, 0.6. **IR (ATR):** ν_{max} (cm^{-1}) = 3433 (w), 2961 (m), 2932 (w), 2858 (w), 1458 (w), 1369 (m), 1249 (m), 1212 (m), 1179 (w), 1165 (w), 1119 (w), 1088 (m), 1073 (s), 1027

(s), 991 (w), 971 (m), 886 (s), 860 (s), 838 (vs), 749 (m), 725 (w), 683 (w). **HRMS (EI):** calc for $C_{18}H_{32}O_4Si$ [*M*]+: 340.2064; found: 340.2081. $[\alpha]_D^{20}$ = –10.3 ° (c = 0.012, $CHCl_3$).

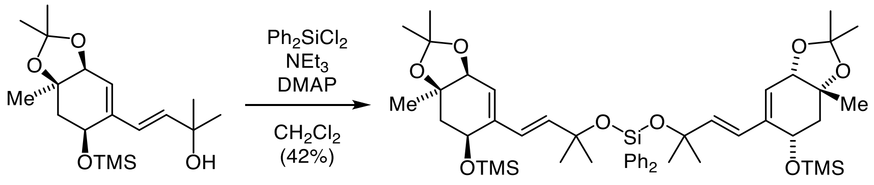

S2 **23**

Siloxane 23: Ph_2SiCl_2 (25 µL, 0.118 mmol) was added to an ice-cooled solution of diene **S2** (80 mg, 0.235 mmol), DMAP (5 mg, 0.041 mmol) and NEt_3 (32 µL, 0.238 mmol) in CH_2Cl_2 (1 mL) and the reaction was stirred 10 min at that temperature and 1 h at room temperature before it was quenched with H_2O and extracted with CH_2Cl_2. The combined organic layers were dried over Na_2SO_4 and concentrated under reduced pressure. Purification of the resulting residue by flash column chromatography (EtOAc:hexanes 10:90) gave siloxane **23** (42 mg, 0.049 mmol, 42%) as a colourless oil.

Data for 23: **^{1}H-NMR (400 MHz, $CDCl_3$):** δ (ppm) = 7.73 – 7.59 (m, 4H), 7.35 (dddd, *J* = 15.2, 8.3, 5.9, 1.2 Hz, 6H), 6.28 – 6.06 (m, 2H), 5.73 (ddd, *J* = 4.8, 1.7, 0.8 Hz, 1H), 4.19 (dd, *J* = 10.2, 5.9 Hz, 1H), 4.08 (d, *J* = 4.7 Hz, 1H), 2.00 – 1.86 (m, 2H), 1.47 (s, 3H), 1.44 (s, 3H), 1.41 (s, 3H), 1.39 (s, 3H), 1.28 (s, 3H), 0.13 (s, 9H). **^{13}C-NMR (100 MHz, $CDCl_3$):** δ (ppm) = 144.0, 140.2, 137.0, 135.4, 129.6, 127.5, 123.8, 119.2, 109.1, 78.3, 76.8, 75.3, 68.5, 42.6, 30.7, 30.5, 28.8, 27.3, 23.7, 0.6. **IR (ATR):** ν_{max} (cm^{-1}) = 2976 (w), 2932 (w), 2858 (w), 1594 (w), 1458 (w), 1430 (w), 1378 (m), 1368 (m), 1342 (w), 1304 (w), 1250 (m), 1240 (m), 1211 (m), 1179 (m), 1164 (m), 1114 (s), 1087 (m), 1072 (s), 1026 (vs), 997(m), 969 (m), 894 (m), 839 (s), 804 (w), 743 (m), 719 (m), 701 (s). **HRMS (EI):** calc. for $C_{48}H_{72}O_8Si_3$ [*M*]+: 860.4535, found: 860.4517. $[\alpha]_D^{20}$ = –16.8 ° (c = 0.008, $CHCl_3$).

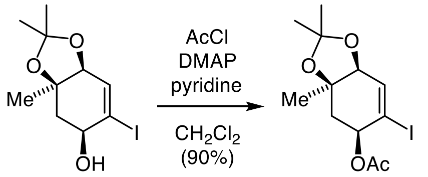

17 **S3**

Acetate S3: Pyridine (1.40 mL, 16.12 mmol) and AcCl (0.64 mL, 8.06 mmol) were sequentially added to an ice-cooled solution of vinyl iodide **17** (500 mg, 1.61 mmol) and DMAP (20 mg, 0.161 mmol) in CH_2Cl_2 (13 mL) and the reaction was stirred for 10 min at that temperature and for 3 h at room temperature before it was hydrolysed with H_2O. The solution was diluted with CH_2Cl_2 and washed with aqueous HCl (1 M), aqueous saturated $NaHCO_3$ and brine. The organic phase was dried over Na_2SO_4 and the solvent was removed under reduced pressure. Purification of the resulting residue by flash column chromatography (EtOAc:hexanes 10:90, R_f = 0.25) gave acetate **S3** as a colorless oil.

Data for S3: ^{1}H-NMR (400 MHz, CDCl$_3$): δ (ppm) = 6.66 (dd, J = 4.8, 1.4 Hz, 1H), 5.37 – 5.29 (m, 1H), 3.99 (d, J = 4.7 Hz, 1H), 2.14 (s, 3H), 2.06 (dd, J = 7.4, 3.8 Hz, 2H), 1.46 (s, 3H), 1.39 (s, 3H), 1.36 (s, 3H). **^{13}C-NMR (100 MHz, CDCl$_3$):** δ (ppm) = 170.1, 137.2, 110.3, 105.3, 78.5, 77.8, 71.7, 38.5, 28.6, 27.66, 24.3, 21.4. **IR (ATR):** ν_{max} (cm^{-1}) = 2983 (w), 2934 (w), 2860 (w), 1741 (s), 1629 (w), 1456 (w), 1377 (m), 1367 (m), 1211 (vs), 1181 (s), 1116 (m), 1080 (m), 1065 (m), 1028 (s), 990 (m), 955 8m), 914 (m), 877 (m), 842 (w), 819 (w), 800 (w), 738 (w), 655 (w) **HRMS (EI):** calc. for $C_{11}H_{14}O_4I$ [$M–CH_3$]$^+$: 336.9931, found: 336.9937. $[\alpha]_D^{20}$ = –7.2 ° (c = 0.009, CHCl$_3$).

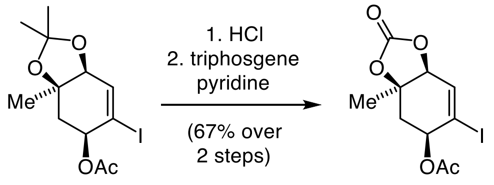

S3 **S4**

Carbonate S4: A solution of acetate **S3** (270 mg, 1.767 mmol) in MeOH (1 mL), THF (1 mL) and aqueous HCl (1 M, 2 mL) was stirred at room temperature overnight. The reaction mixture was directly submitted to flash column chromatography (hexanes:EtOAC 1:1) giving diol **S4.1** (229 mg, 0.734 mmol) as a colorless oil.

A solution of triphosgene (110 mg, 0.379 mmol) in CH_2Cl_2 (3 mL) was added dropwise to stirred solution of triol **S4.1** (229 mg, 0.734 mmol) and pyridine (0.36 mL, 4.45 mmol) in CH_2Cl_2 (7.7 mL) at –78 °C. The reaction was stirred for 30 min at –78 °C and overnight at room temperature before it was quenched by the addition of aqueous saturated NH_4Cl (7 mL). The organic phase was separated and washed with aqueous HCl (1 M), aqueous saturated NH_4Cl and brine and dried over $MgSO_4$. Evaporation of the solvent under reduced pressure and purification of the resulting residue by flash column chromatography

(EtOAc:hexanes 3:7, R_f = 0.3) gave carbonate **S4** (174 mg, 0.515 mmol, 67% over 2 steps) as a colorless oil.

Data for S4: ^{1}H-NMR (400 MHz, CDCl$_3$): δ (ppm) = 6.59 (d, J = 2.9 Hz, 1H), 5.44 (t, J = 4.6 Hz, 1H), 3.84 – 3.77 (m, 1H), 2.14 (d, J = 3.6 Hz, 4H), 2.08 (dd, J = 14.8, 5.3 Hz, 1H), 1.27 (s, 3H). **^{13}C-NMR (100 MHz, CDCl$_3$):** δ (ppm) = 169.6, 144.1, 97.0, 73.8, 72.6, 68.8, 39.0, 25.1, 21.1. **IR (ATR):** ν_{max} (cm^{-1}) = 1789 (s), 1772 (s), 1752 (s), 1730 (s), 1623 (s), 1556 (s), 1461 (s), 1432 (s), 1340 (s), 1385 (s), 1372 (s), 1346 (s), 1306 (s), 1290 (m), 1242 (s), 1206 (vs), 1153 (w), 1110 (m), 1077 (w), 1057 (m), 1018 (vs), 996 (s), 970 8m), 959 (s), 940 (m), 899 (m), 860 (m), 814 (w)=, 778 (m), 760 (m), 715 (w). **HRMS (EI):** calc. $C_{10}H_{12}IO_5$ for $[M+H]^+$: 338.9724, found: 338.9722. $[\alpha]_D^{20}$ = –72 ° (c = 0.0125, CHCl$_3$).

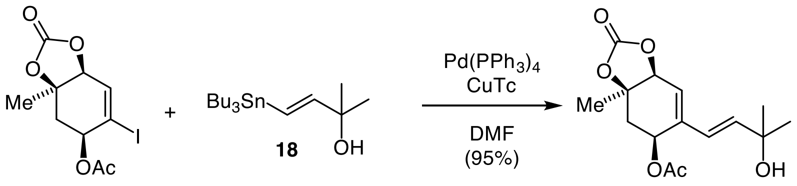

S4 S5

Allylic alcohol S5: A solution of carbonate **S4** (50 mg, 0.148 mmol), vinylstannane **18** (111 mg, 0.296 mmol), Pd(PPh$_3$)$_4$ (15 mg, 0.013 mmol) and CuTc (35 mg, 0.184 mmol) in DMF (4.5 mL) was stirred at 65 °C overnight in the dark. The reaction was cooled to room temperature, diluted with EtOAc and filtered through a pad of celite. Evaporation of the solvent under reduced pressure and purification of the resulting residue by flash column chromatography (EtOAc:hexanes 2:1, R_f = 0.25) gave allylic alcohol **S5** (41 mg, 0.140 mmol, 95%) as a colorless oil.

Data for S5: ^{1}H-NMR (400 MHz, CDCl$_3$): δ (ppm) = 6.25 (d, J = 16.1 Hz, 1H), 5.99 – 5.92 (m, 2H), 5.65 (s, 1H), 4.85 (d, J = 4.5 Hz, 1H), 2.58 – 2.51 (m, 1H), 2.07 (s, 3H), 1.89 (dd, J = 15.9, 4.1 Hz, 1H), 1.55 (s, 3H), 1.34 (s, 6H). **^{13}C-NMR (100 MHz, CDCl$_3$):** δ (ppm) = 171.0, 153.8, 141.4, 140.0, 124.4, 123.6, 79.5, 77.4, 71.1, 62.9, 36.0, 29.9, 29.9, 27.0, 21.4. **IR (ATR):** ν_{max} (cm^{-1}) = 3482 (w), 2973 (w), 2932 (w), 1782 (s), 1732 (s), 1457 (w), 1426 (w), 1373 (m), 1338 (m), 1304 (w), 1224 (s), 1202 (s), 1187 (s), 1133 (m), 1075 (m), 1017 (vs), 996 (m), 971 (m), 912 (w), 847 (w), 816 (w), 752 (s). **HRMS (EI):** calc. for $C_{15}H_{20}O_6$ $[M]^+$: 296.1254, found: 296.1258. $[\alpha]_D^{20}$ = –65.9 ° (c = 0.06, CHCl$_3$).

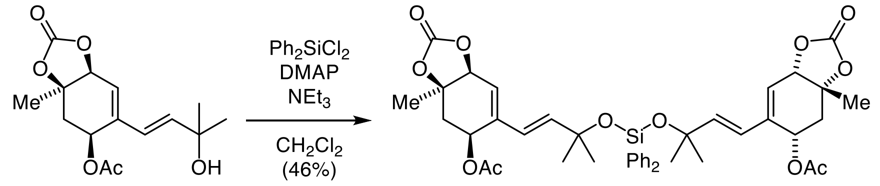

S5 **25**

Siloxane 25: NEt_3 (18 μL, 0.129 mmol) and Ph_2SiCl_2 (12 μL, 0.057 mmol) were sequentially added to an ice-cooled solution of allylic alcohol **S5** (40 mg, 0.135 mmol) in CH_2Cl_2 (1 mL) and the reaction was stirred for 10 min at that temperature and for 3 h at room temperature. The mixture was diluted with H_2O and extracted with CH_2Cl_2. The organic phase was washed with brine and dried over $MgSO_4$. Evaporation of the solvent and purification of the resulting residue by flash column chromatography (EtOAc:hexanes 2:2-1:1) gave siloxane **25** (24 mg, 0.031 mmol, 46%) as a colourless oil.

Data for 25: ^{1}H-NMR (400 MHz, $CDCl_3$): δ (ppm) = 7.59 (dt, J = 6.6, 1.5 Hz, 2H), 7.41 – 7.35 (m, 1H), 7.31 (dd, J = 7.8, 6.5 Hz, 2H), 6.14 (dd, J = 16.0, 13.2 Hz, 1H), 5.92 – 5.76 (m, 2H), 5.51 (t, J = 3.6 Hz, 1H), 4.83 (d, J = 4.3 Hz, 1H), 2.52 (dd, J = 15.9, 3.1 Hz, 1H), 1.98 (d, J = 3.0 Hz, 3H), 1.85 (dt, J = 15.9, 4.5 Hz, 1H), 1.54 (s, 3H), 1.34 (d, J = 2.4 Hz, 3H), 1.31 (s, 3H). **^{13}C-NMR (100 MHz, $CDCl_3$):** δ (ppm) = 170.8, 153.9, 141.6, 140.0, 136.2, 135.3, 130.0, 127.7, 124.3, 123.2, 79.6, 77.4, 77.2, 75.1, 62.9, 35.8, 30.4, 30.3, 26.9, 21.3. **IR (ATR):** ν_{max} (cm^{-1}) = 2974 (m), 2156 (w), 1802 (vs), 1737 (s), 1429 (m), 1382 (m), 1234 (s), 114 (m), 1076 (m), 1024 (s), 752 (m), 704 (m). **HRMS (EI):** calc. for $C_{42}H_{48}O_{12}Si$ $[M]^+$: 772.2910, found: 772.2913. $[\alpha]_D^{20}$ = –86.4 ° (c = 0.004, $CHCl_3$).

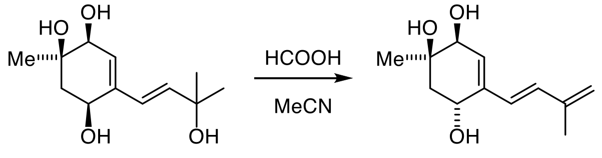

5 **6**

Acremine T 6: HCOOH (5 drops) were added to a solution of acremine F (**5**) (20 mg, 0.075 mmol) in MeCN (5 mL) and the resulting solution was stirred at room temperature for 36 h. The reaction mixture was diluted with EtOAc, washed with aqueous saturated $NaHCO_3$ and brine, dried over $MgSO_4$ and concentrated under reduced pressure. The resulting residue was purified by flash column chromatography (EtOAc:hexanes 2:1) to give crude acremine T **6** (4.6 mg).

Data for 6: ^{1}H-NMR (400 MHz, d_4-MeOH): δ (ppm) = 6.60 (d, J = 16.2 Hz, 1H), 6.19 (d, J = 16.2 Hz, 1H), 5.68 (d, J = 3.0 Hz, 1H), 5.02 (br s, 1H), 5.01 (br s, 1H), 4.40 (t, J = 4.0 Hz, 1H), 3.96 (d, J = 2.9 Hz, 1H), 2.08 (dd, J = 14.4, 3.3 Hz, 1H), 1.88 (s, 3H), 1.85 (dd, J = 14.4, 4.7 Hz, 1H), 1.26 (s, 3H). **^{13}C-NMR (100 MHz, d_4-MeOH):** δ (ppm) = 143.5, 139.8, 133.3, 131.7, 129.8, 117.4, 73.9, 72.0, 65.4, 42.0, 26.5, 18.6.

Table 4: 1H- and 13C-NMR chemical shifts in **d_4-MeOH** of natural[4] vs. synthetic Acremine T (6).

No.	**^{1}H-NMR Isolated 600 MHz, $CDCl_3$ [ppm]**	**1H-NMR Synthetic 400 MHz, $CDCl_3$ [ppm]**	**^{13}C-NMR Isolated 125 MHz, $CDCl_3$ [ppm]**	**^{13}C-NMR Synthetic 100 MHz, $CDCl_3$ [ppm]**
1			139.8	139.8
2	5.68 (d, J = 3.0 Hz, 1H)	5.68 (d, J = 3.0 Hz, 1H)	131.7	131.7
3	3.96 (d, J = 3.0 Hz, 1H)	3.96 (d, J = 2.9 Hz, 1H)	73.9	73.9
4			72.0	72.0
5a	1.85 (dd, J = 14.3, 4.6 Hz, 1H)	1.85 (dd, J = 14.4, 4.7 Hz, 1H)	42.0	42.0
5b	2.09 (dd, J = 14.3, 3.3 Hz, 1H)	2.08 (dd, J = 14.4, 3.3 Hz, 1H)		
6	4.40 (dd, J = 4.6 Hz, 3,3 Hz 1H)	4.40 (t, J = 4.0 Hz, 1H)	65.4	65.4
7	6.19 (br d, J = 16.0 Hz, 1H)	6.19 (d, J = 16.2 Hz, 1H)	129.8	129.8
8	6.60 (br d, J = 16.0Hz, 1H)	6.60 (d, J = 16.2 Hz, 1H)	133.3	133.3
9			143.5	143.5
10a	5.02 (br s, 1H)	5.02 (br s, 1H)	117.4	117.4
10b	5.01 (br s, 1H)	5.01 (br s, 1H)		
11	1.88 (s, 3H)	1.88 (s, 3H)	18.6	18.6
12	1.26 (s, 3H)	1.26 (s, 3H)	26.5	26.5

NMR Spectra

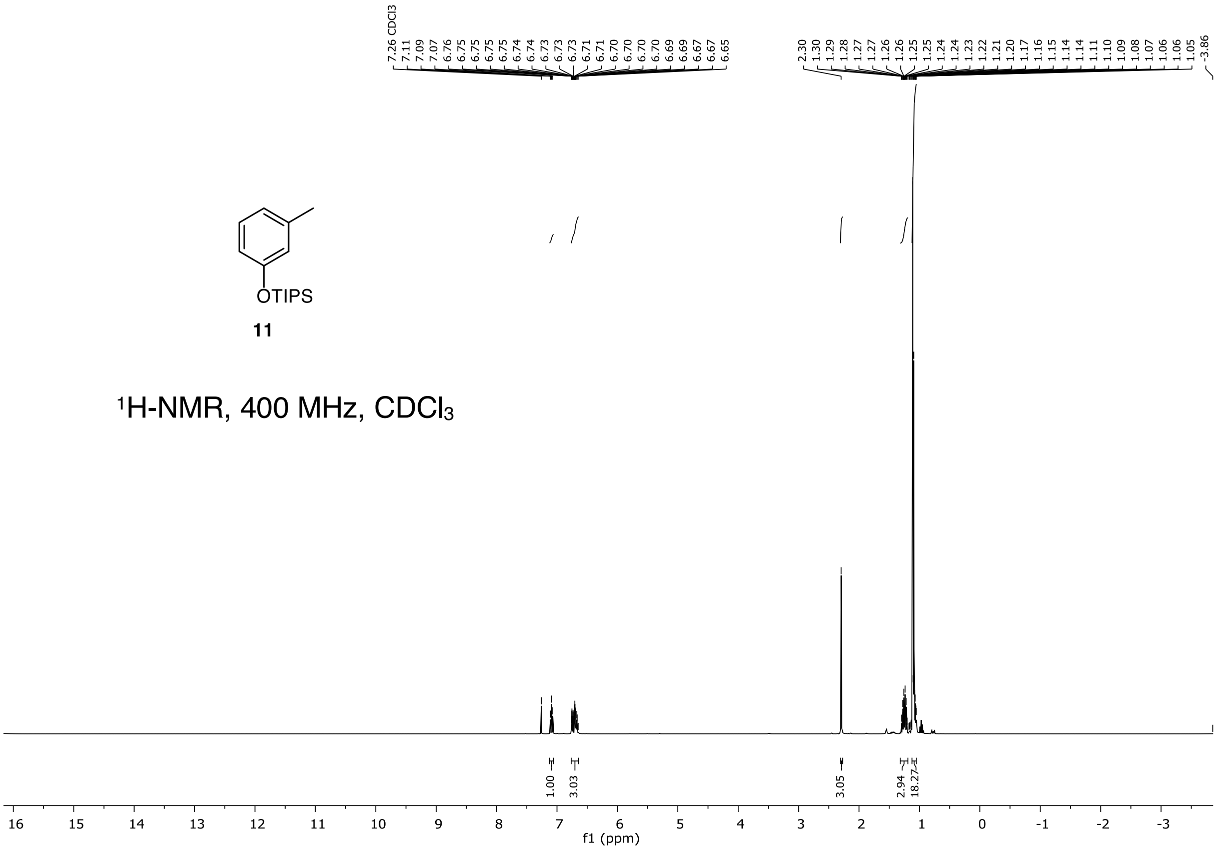

7.26 CDCl3
7.11
7.09
7.07
6.76
6.75
6.75
6.75
6.75
6.74
6.73
6.73
6.73
6.73
6.71
6.71
6.70
6.70
6.70
6.69
6.69
6.67
6.67
6.65
2.30
1.30
1.29
1.28
1.27
1.27
1.26
1.26
1.25
1.25
1.24
1.24
1.23
1.22
1.21
1.20
1.17
1.16
1.15
1.14
1.14
1.11
1.10
1.09
1.08
1.07
1.06
1.06
1.05
-3.86
OTIPS
11
^{1}H-NMR, 400 MHz, CDCl$_3$
1.00
3.03
3.05
2.94
18.27
f1 (ppm)

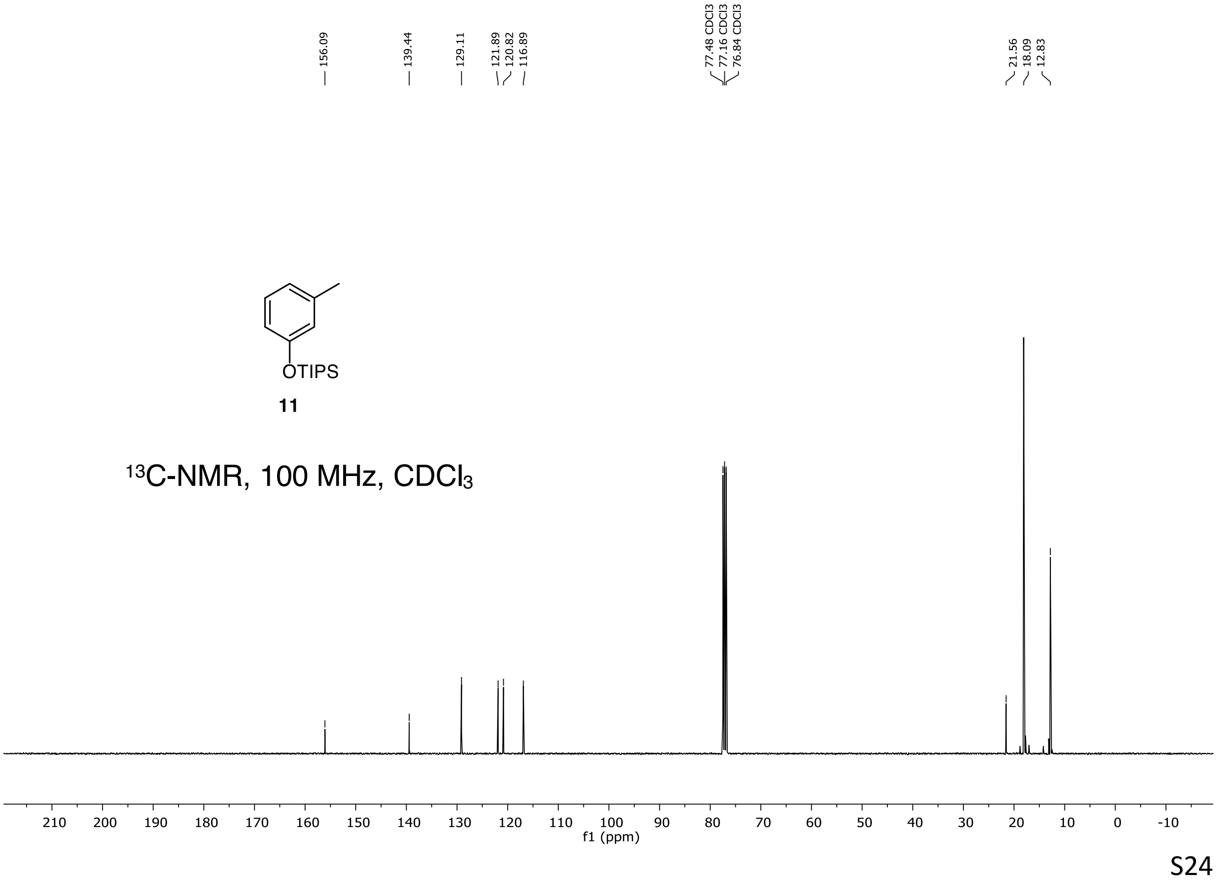

156.09
139.44
129.11
121.89
120.82
116.89
77.48 CDCl3
77.16 CDCl3
76.84 CDCl3
21.56
18.09
12.83
OTIPS
11
^{13}C-NMR, 100 MHz, CDCl$_3$
f1 (ppm)

13

^{1}H-NMR, 400 MHz, $CDCl_3$

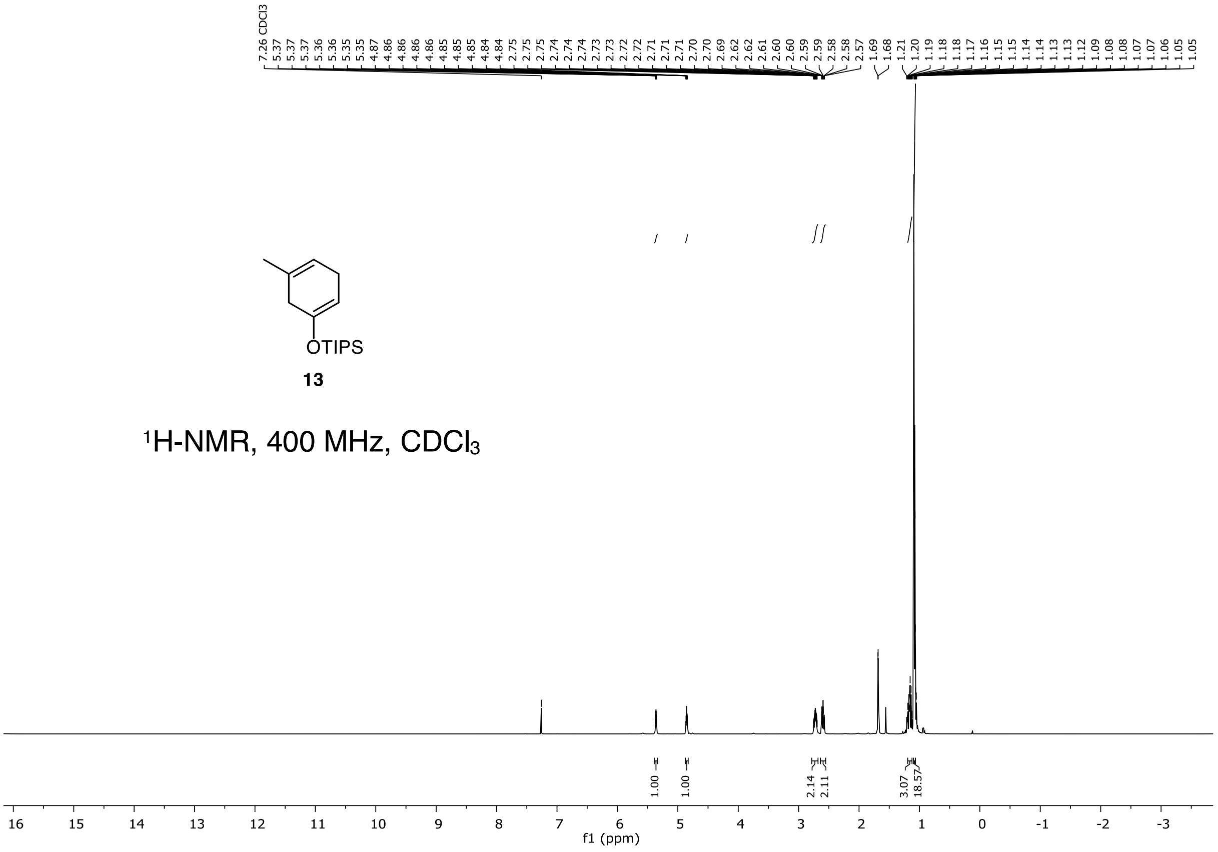

13

^{13}C-NMR, 100 MHz, $CDCl_3$

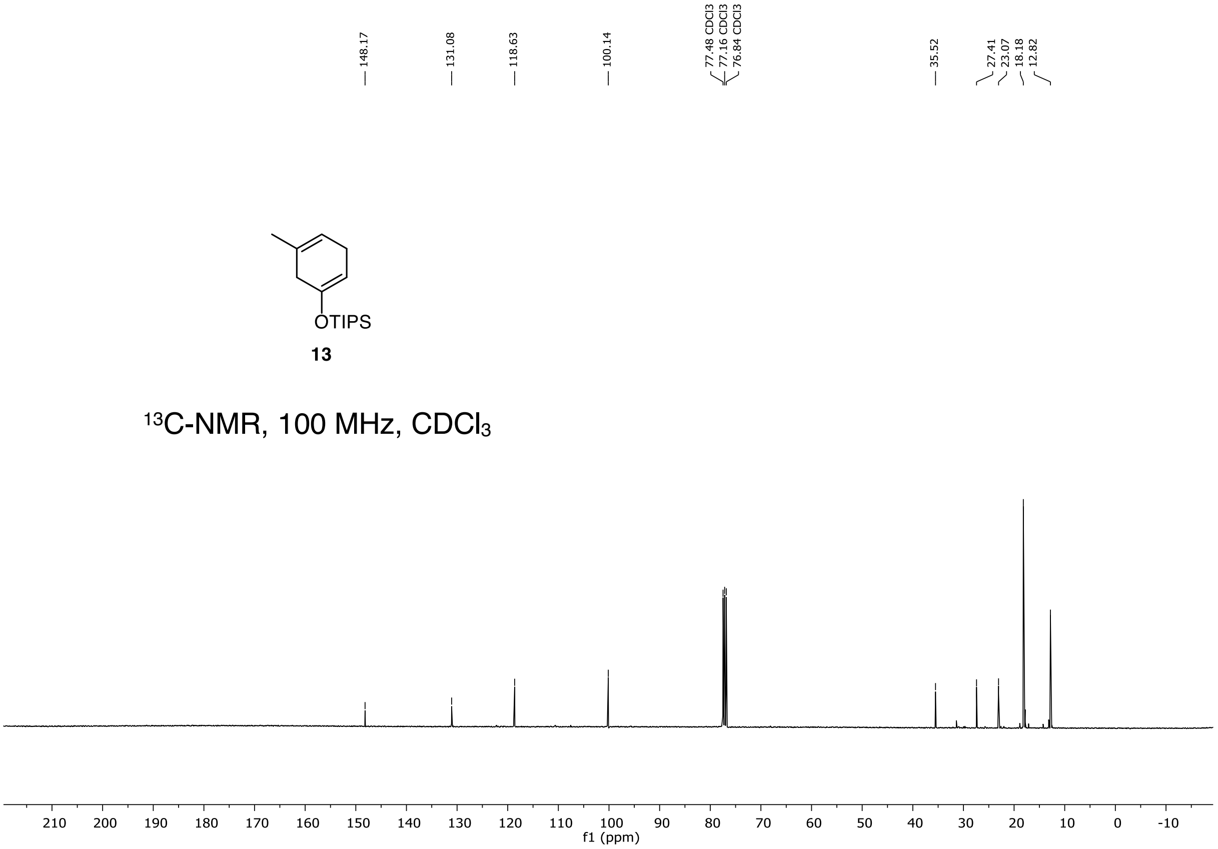

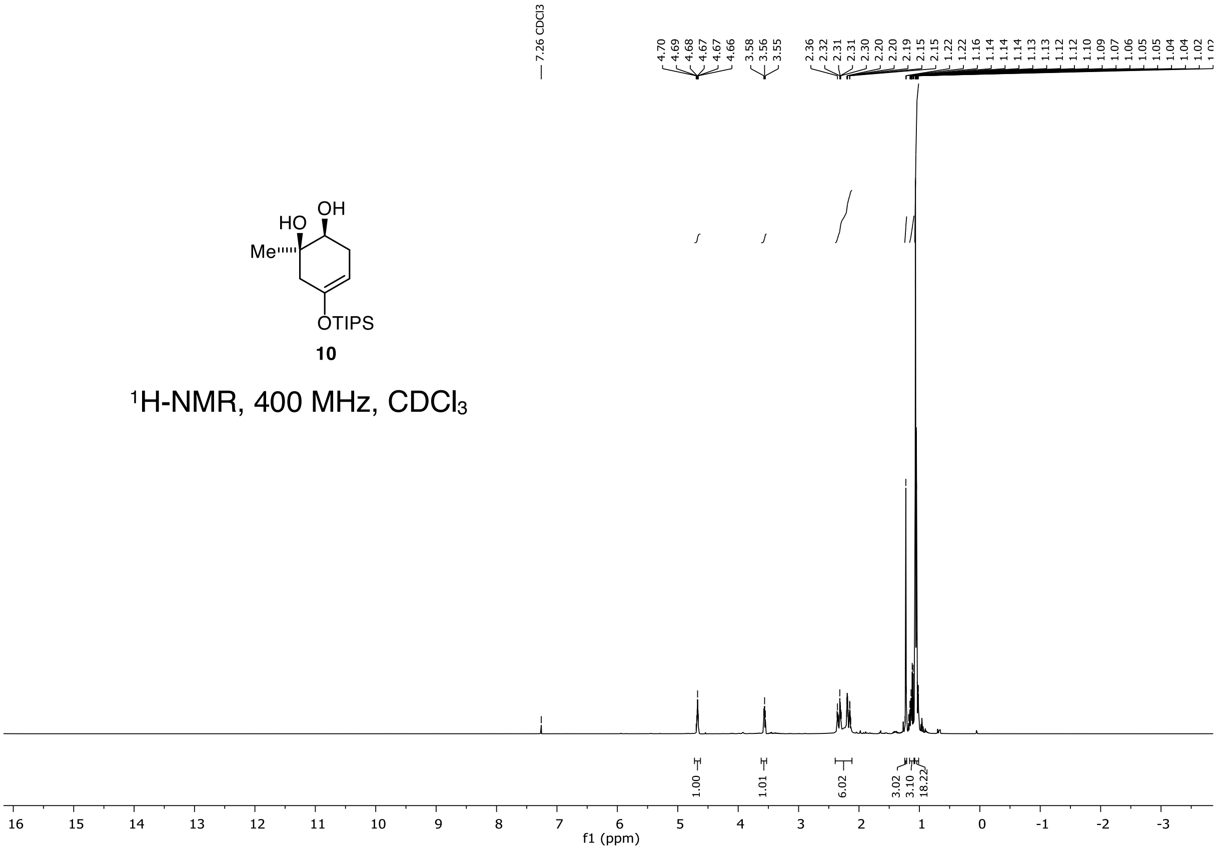
7.26 CDCl3
4.70 4.69 4.68 4.67 4.67 4.66 3.58 3.56 3.55
2.36 2.32 2.31 2.31 2.30 2.20 2.20 2.19 2.15 2.15 1.22 1.16 1.14 1.14 1.14 1.13 1.13 1.12 1.12 1.10 1.09 1.07 1.06 1.05 1.05 1.04 1.04 1.02
HO OH
Me
OTIPS
10
1H-NMR, 400 MHz, CDCl3
1.00 1.01 6.02 3.02 3.10 18.22
16 15 14 13 12 11 10 9 8 7 6 5 4 3 2 1 0 -1 -2 -3
f1 (ppm)

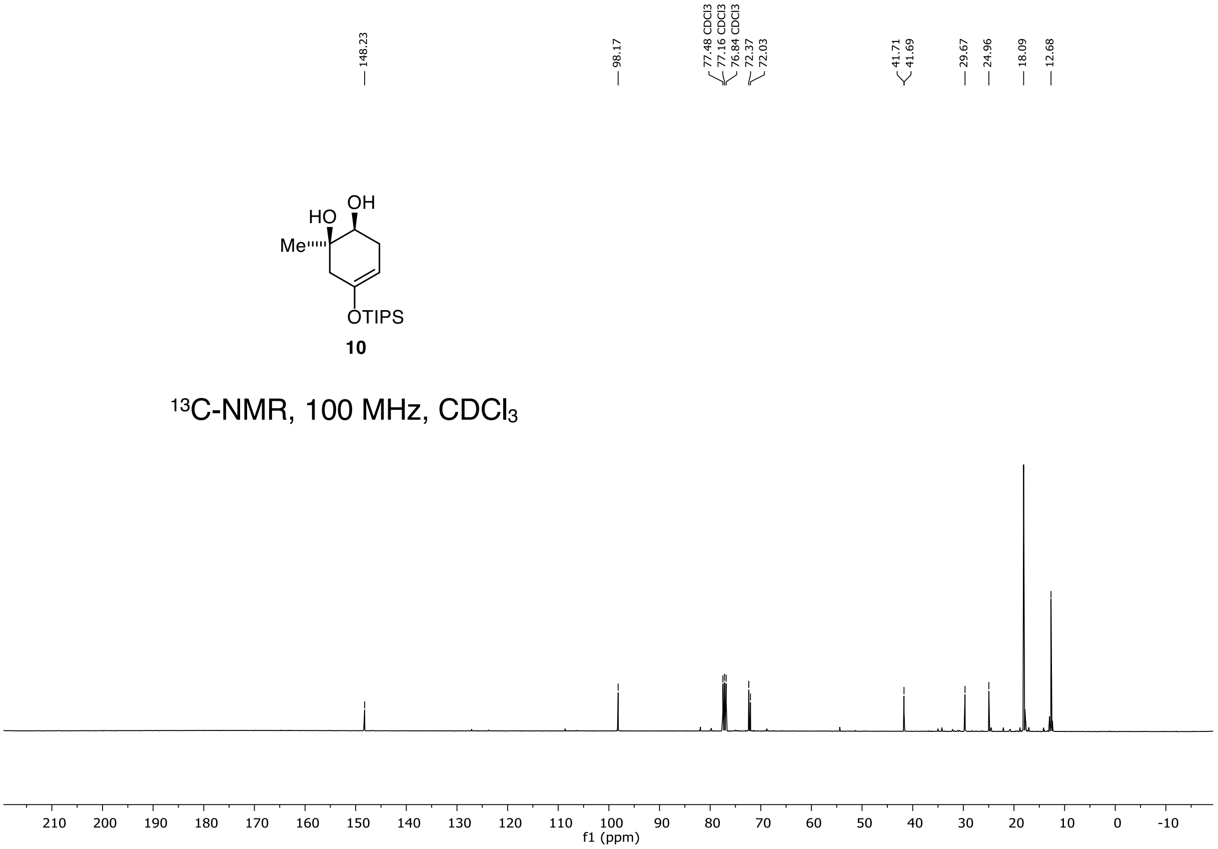
148.23
98.17
77.48 CDCl3 77.16 CDCl3 76.84 CDCl3 72.37 72.03
41.71 41.69
29.67 24.96 18.09 12.68
HO OH
Me
OTIPS
10
13C-NMR, 100 MHz, CDCl3
210 200 190 180 170 160 150 140 130 120 110 100 90 80 70 60 50 40 30 20 10 0 -10
f1 (ppm)

14

^{1}H-NMR, 400 MHz, $CDCl_3$

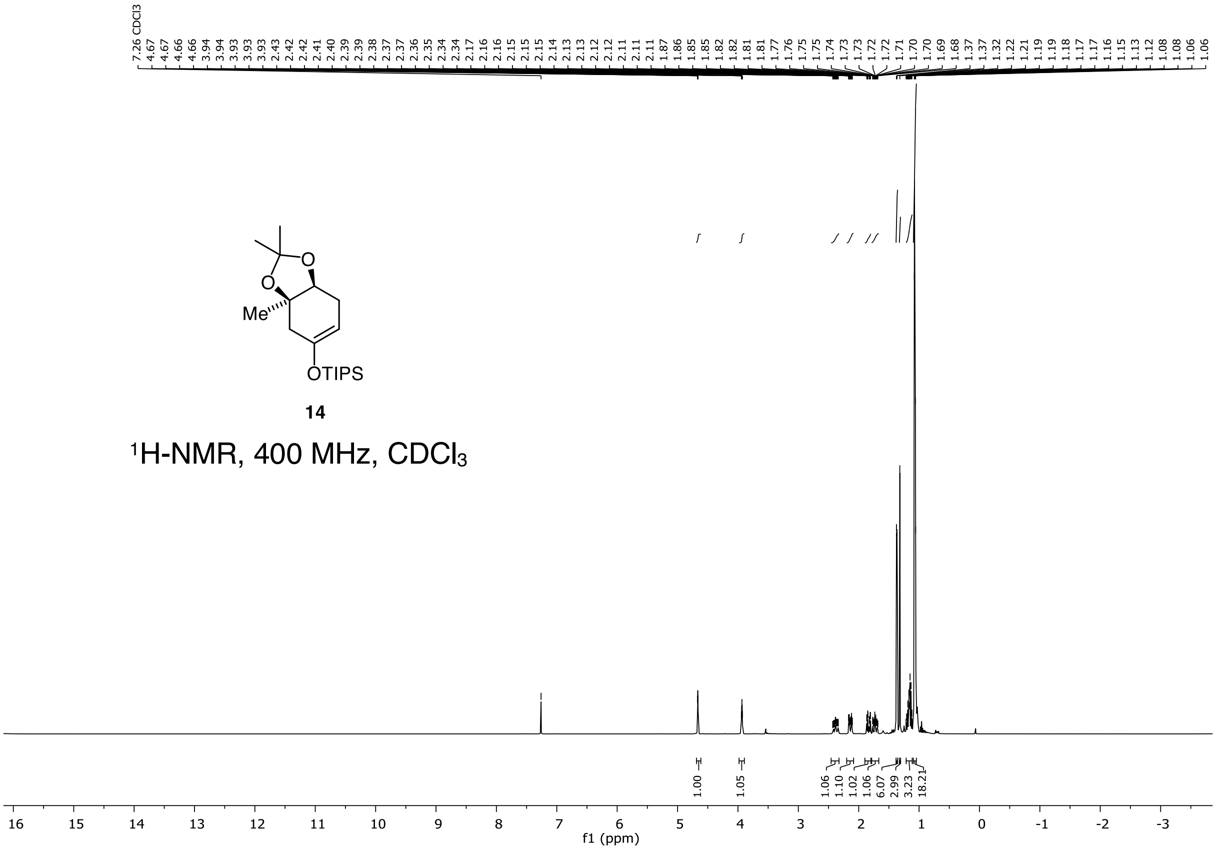

14

^{13}C-NMR, 100 MHz, $CDCl_3$

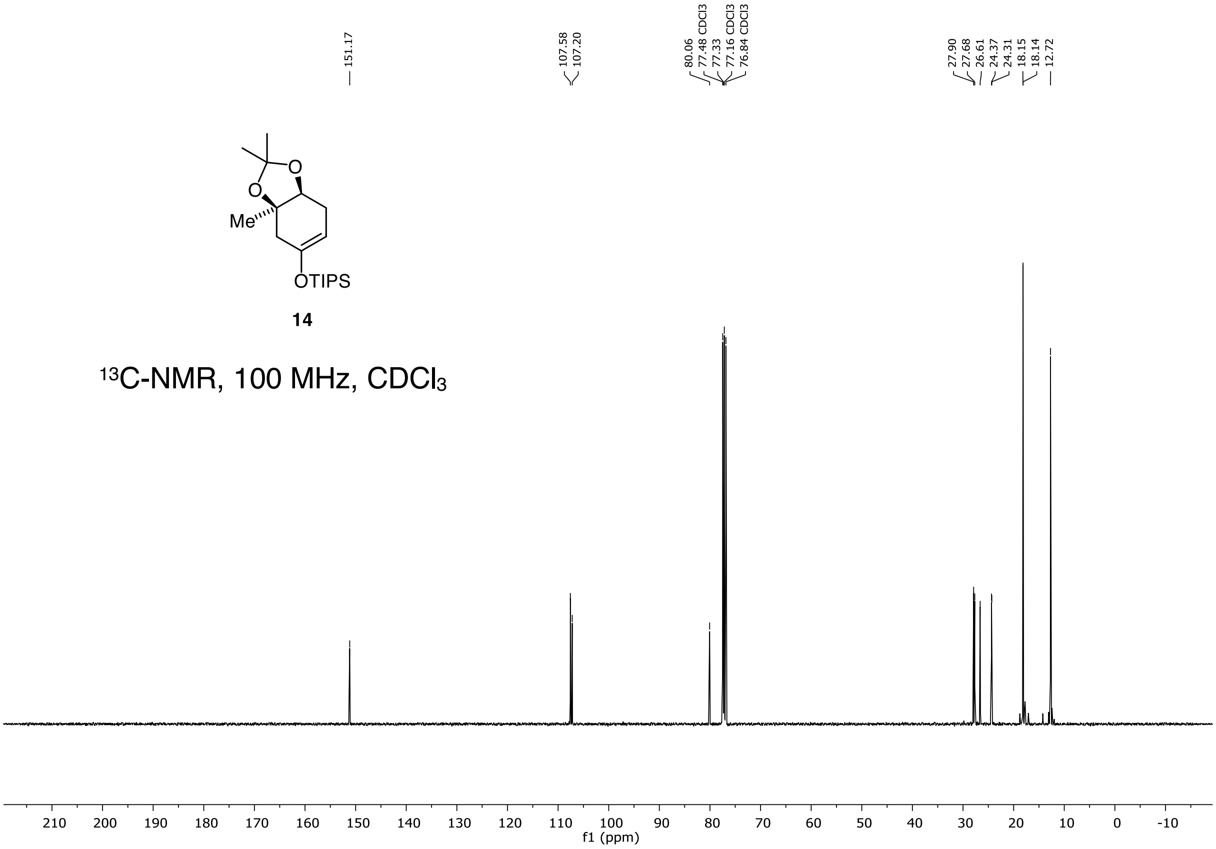

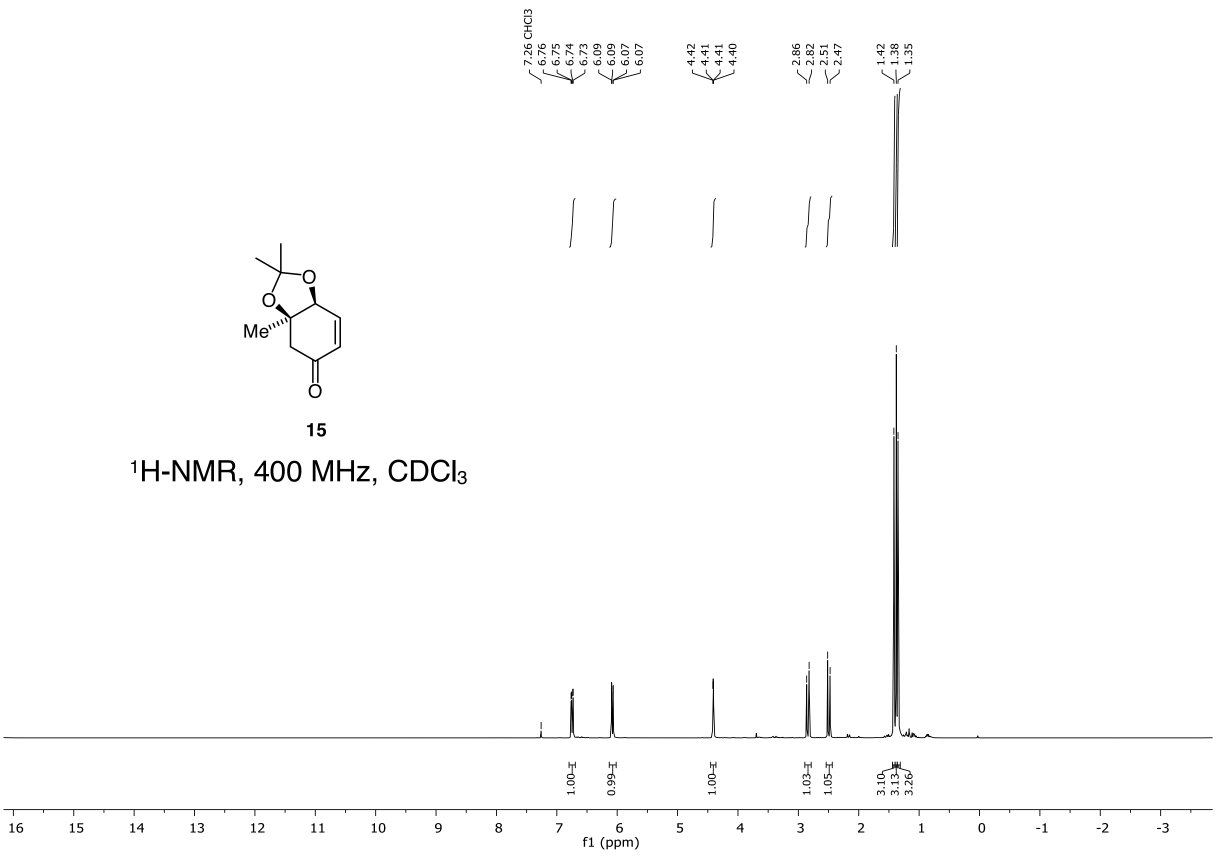

15

^{1}H-NMR, 400 MHz, $CDCl_3$

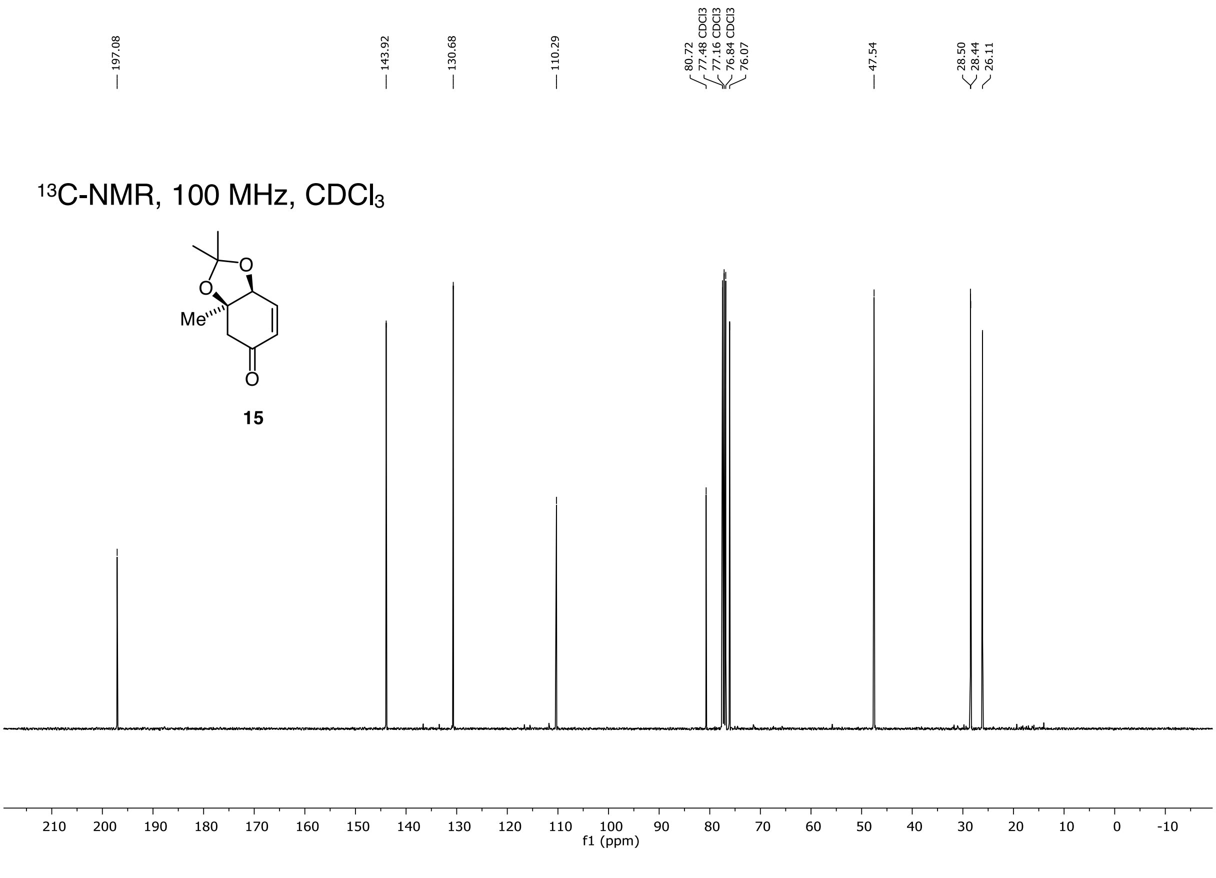

15

^{13}C-NMR, 100 MHz, $CDCl_3$

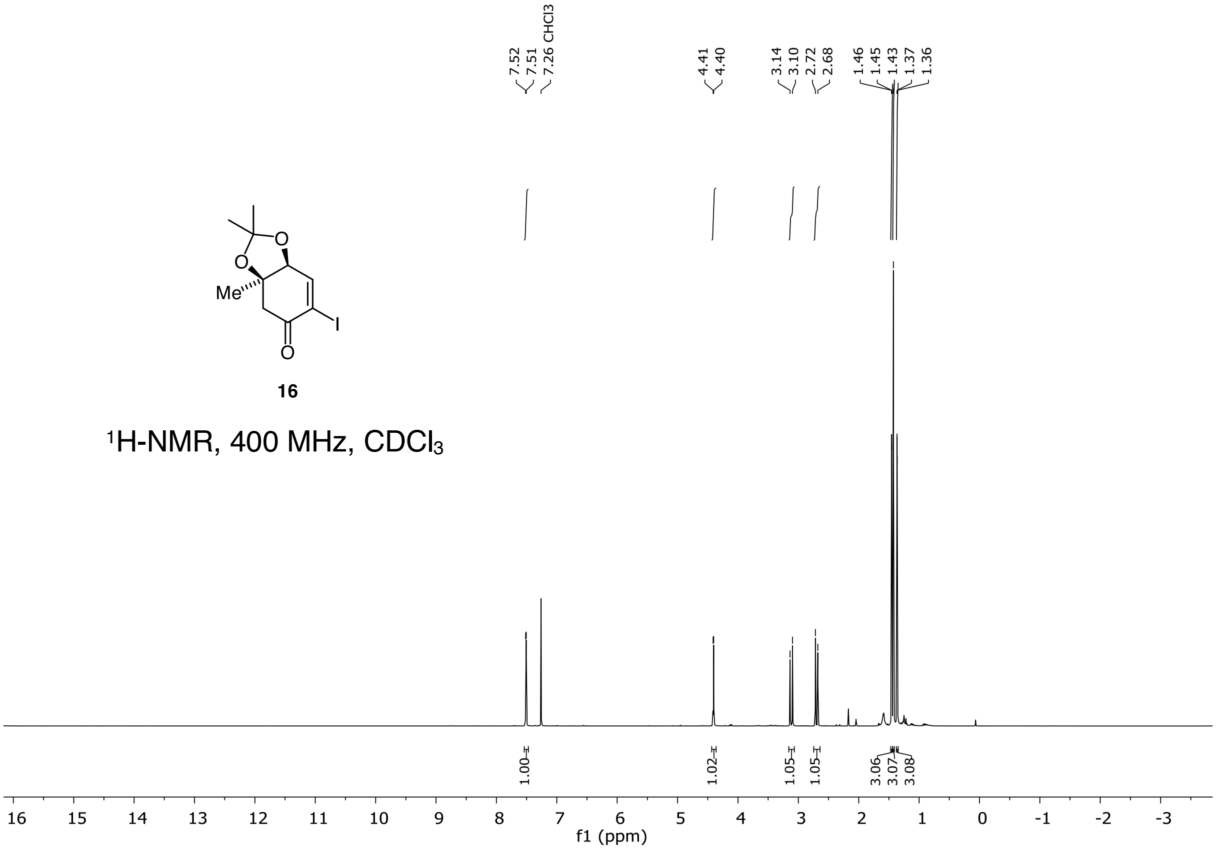

16

^{1}H-NMR, 400 MHz, $CDCl_3$

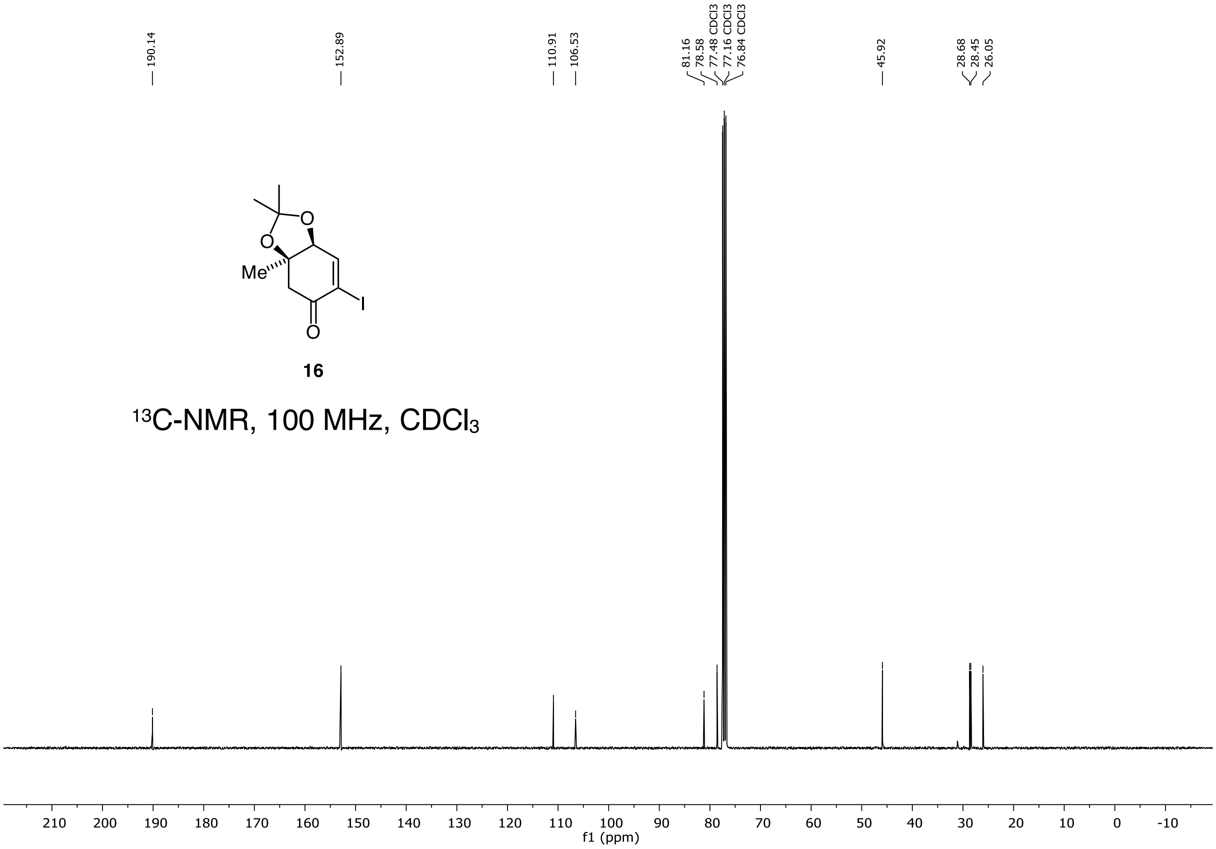

16

^{13}C-NMR, 100 MHz, $CDCl_3$

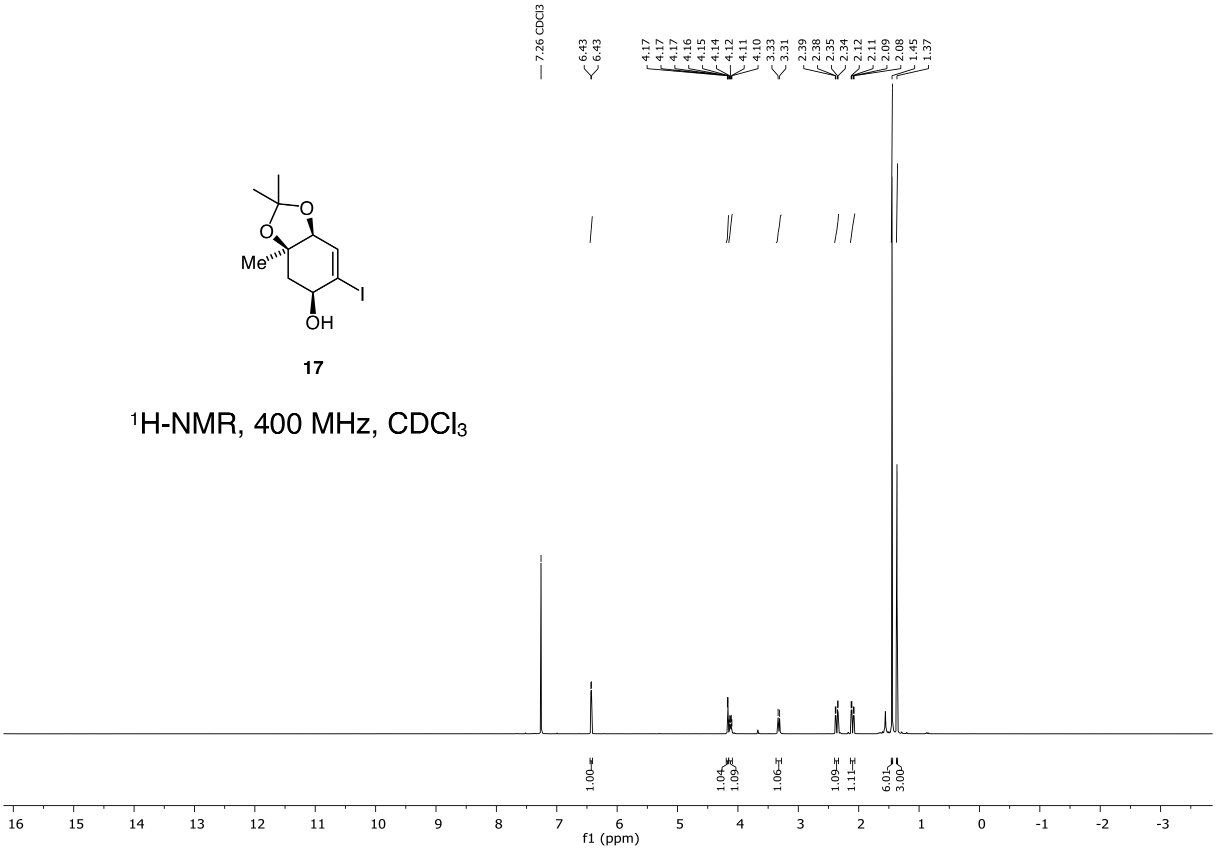

17

^{1}H-NMR, 400 MHz, $CDCl_3$

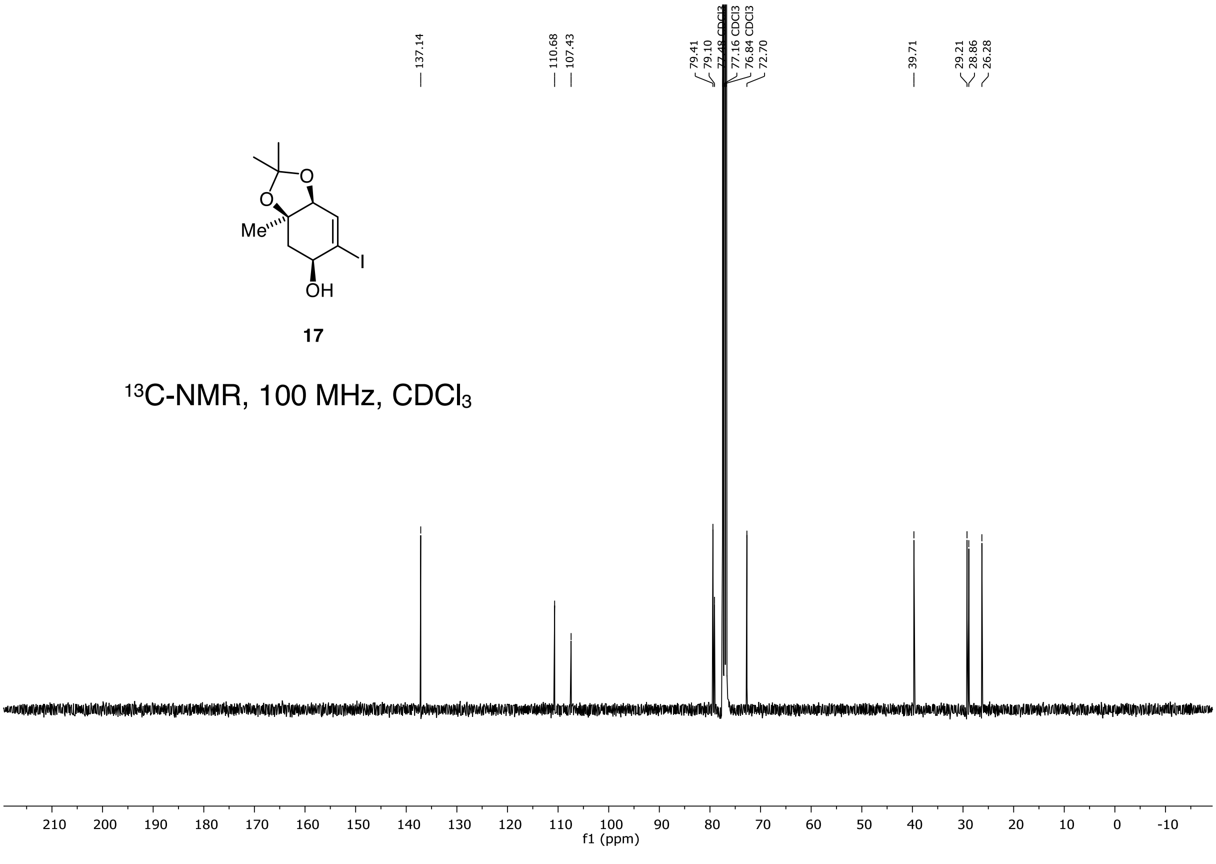

17

^{13}C-NMR, 100 MHz, $CDCl_3$

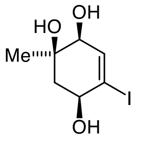

9

^{1}H-NMR, 400 MHz, d_4-MeOH

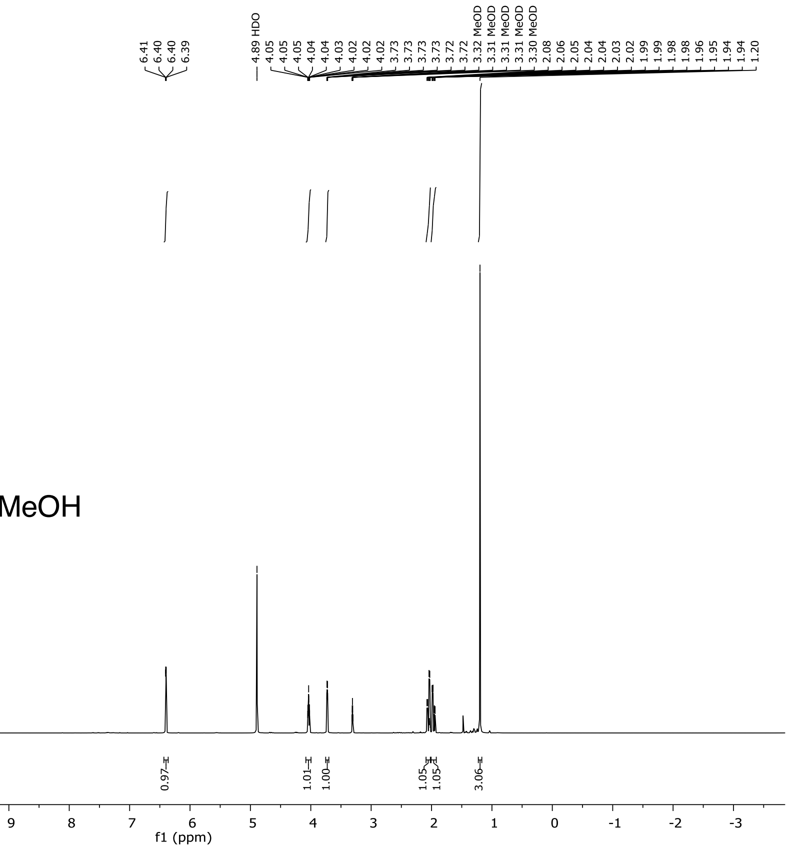

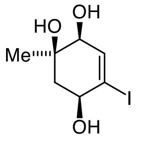

9

^{13}C-NMR, 100 MHz, d_4-MeOH

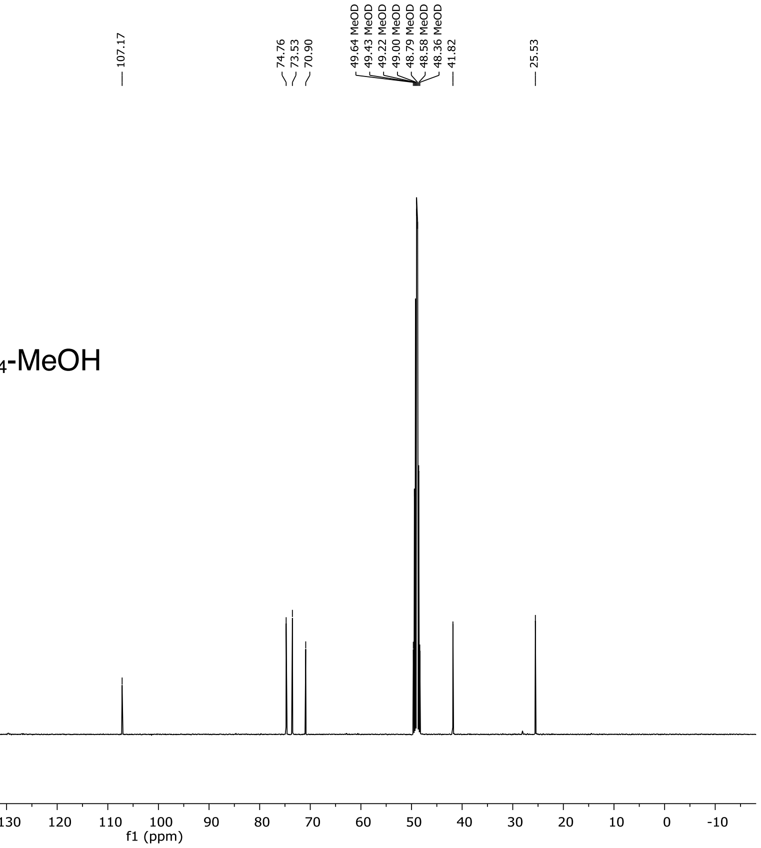

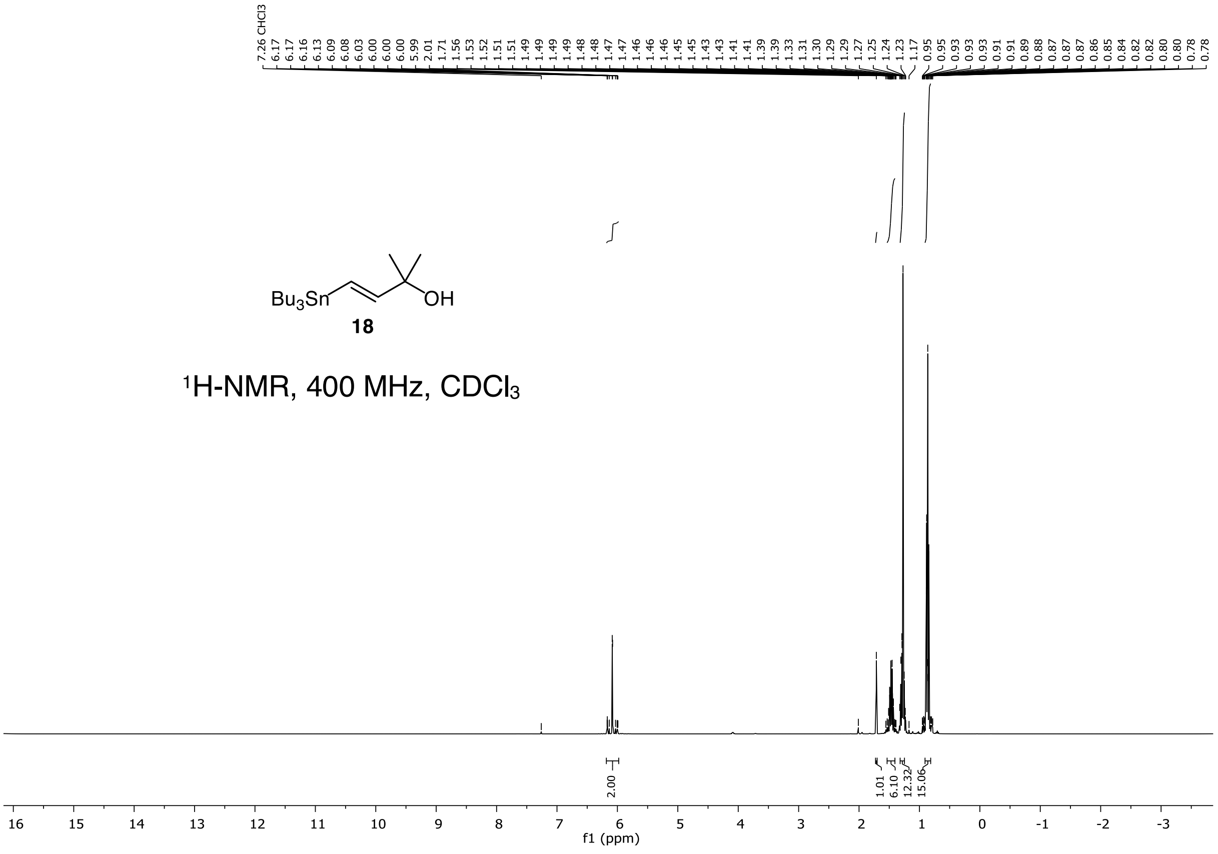
Bu3Sn
OH
18
1H-NMR, 400 MHz, CDCl3
2.00
1.01
6.10
12.32
15.06
f1 (ppm)

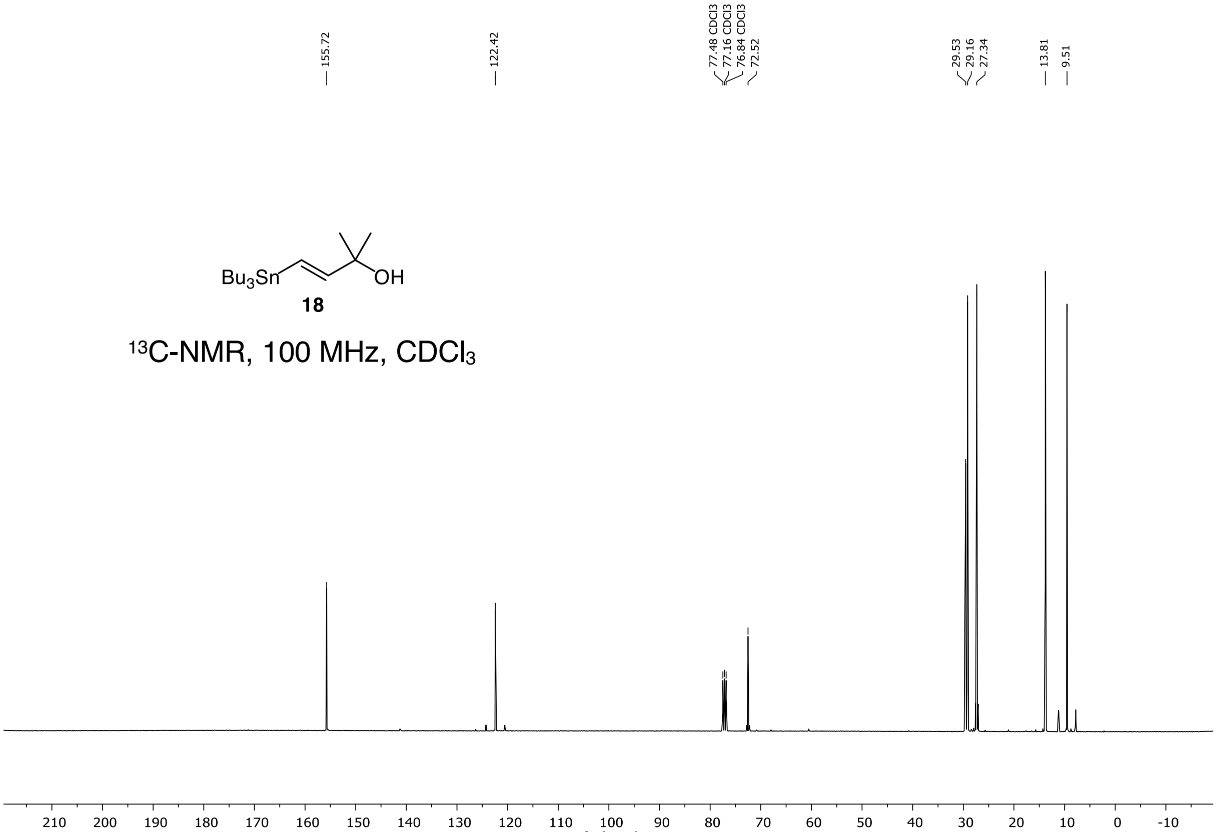
155.72
122.42
77.48 CDCl3
77.16 CDCl3
76.84 CDCl3
72.52
29.53
29.16
27.34
13.81
9.51
Bu3Sn
OH
18
13C-NMR, 100 MHz, CDCl3
f1 (ppm)

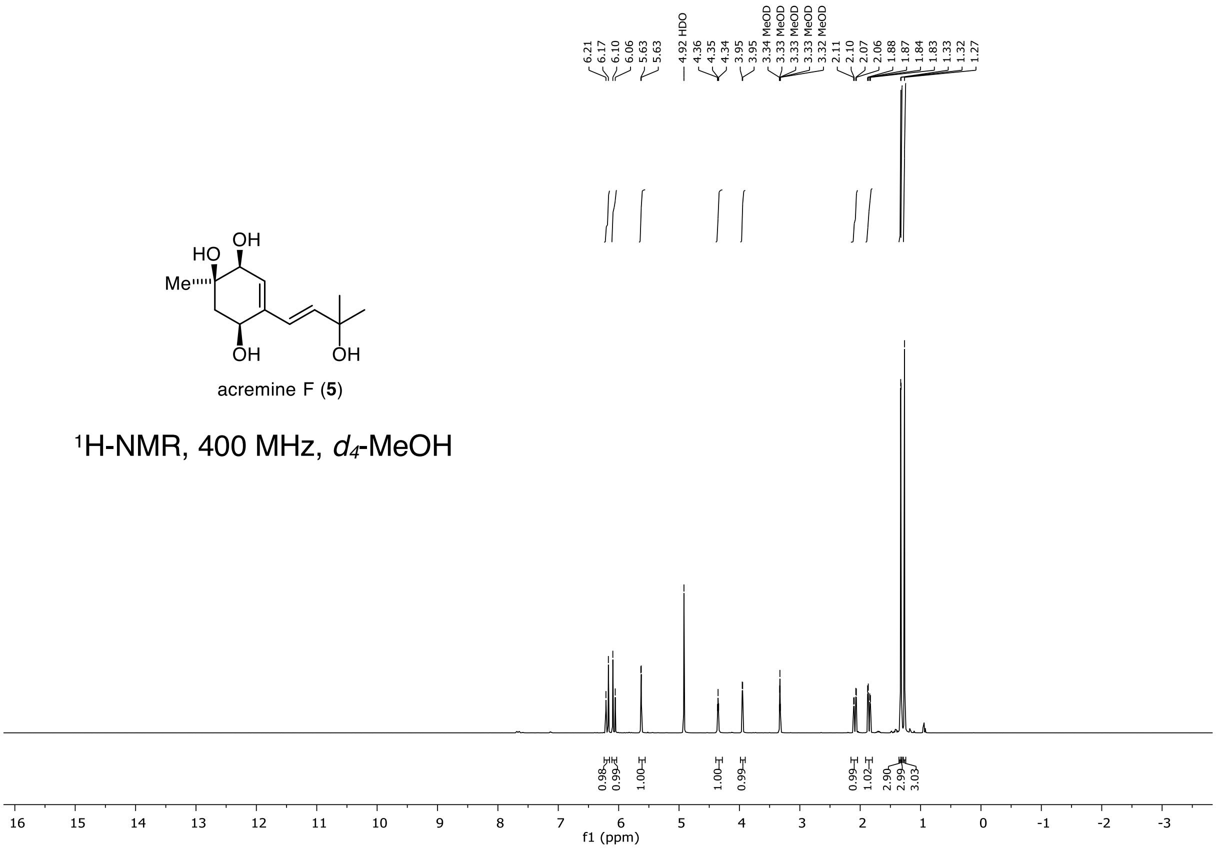
6.21
6.17
6.10
6.06
5.63
5.63
4.92 HDO
4.36
4.35
4.34
3.95
3.95
3.34 MeOD
3.33 MeOD
3.33 MeOD
3.33 MeOD
3.32 MeOD
2.11
2.10
2.07
2.06
1.88
1.87
1.84
1.83
1.33
1.32
1.27
HO
OH
Me
OH
OH
acremine F (5)
1H-NMR, 400 MHz, d4-MeOH
0.98
0.99
1.00
1.00
0.99
0.99
1.02
2.90
2.99
3.03
f1 (ppm)

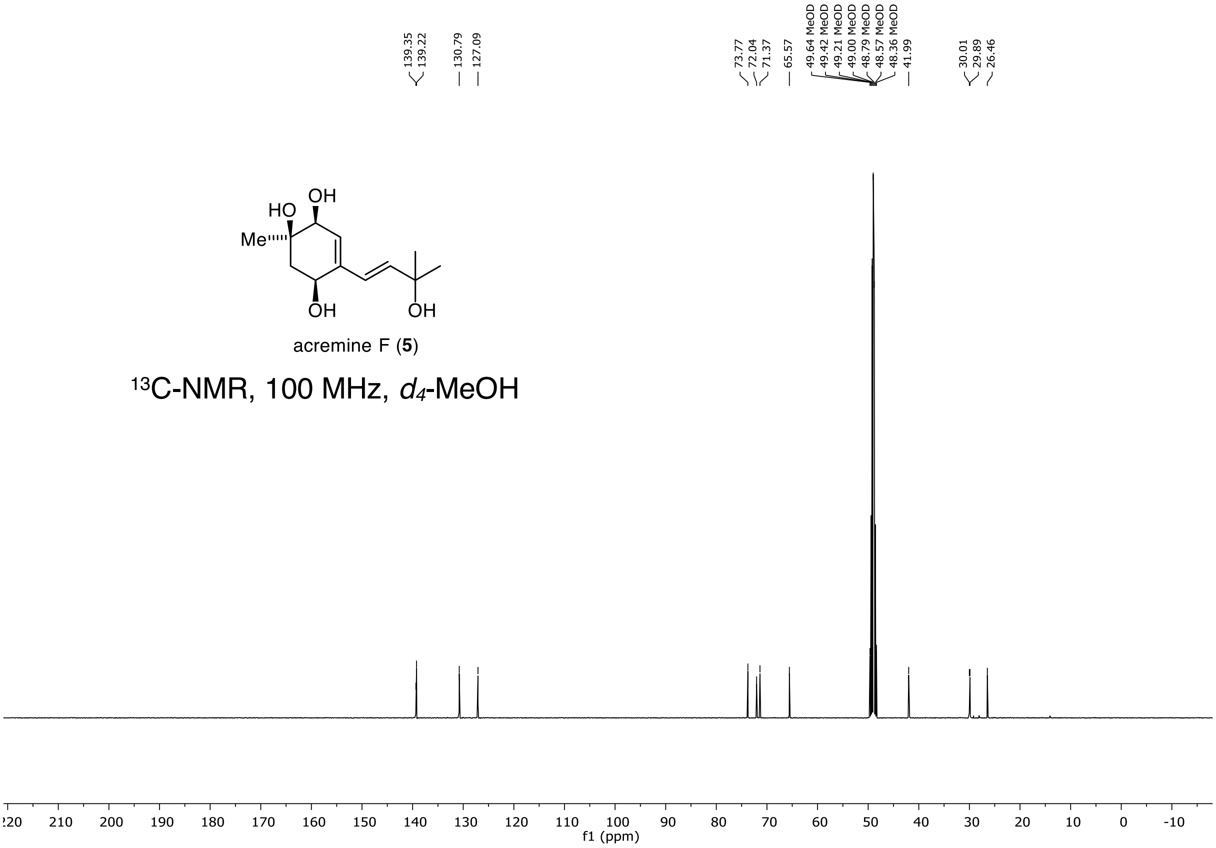
139.35
139.22
130.79
127.09
73.77
72.04
71.37
65.57
49.64 MeOD
49.42 MeOD
49.21 MeOD
49.00 MeOD
48.79 MeOD
48.57 MeOD
48.36 MeOD
41.99
30.01
29.89
26.46
HO
OH
Me
OH
OH
acremine F (5)
13C-NMR, 100 MHz, d4-MeOH
f1 (ppm)

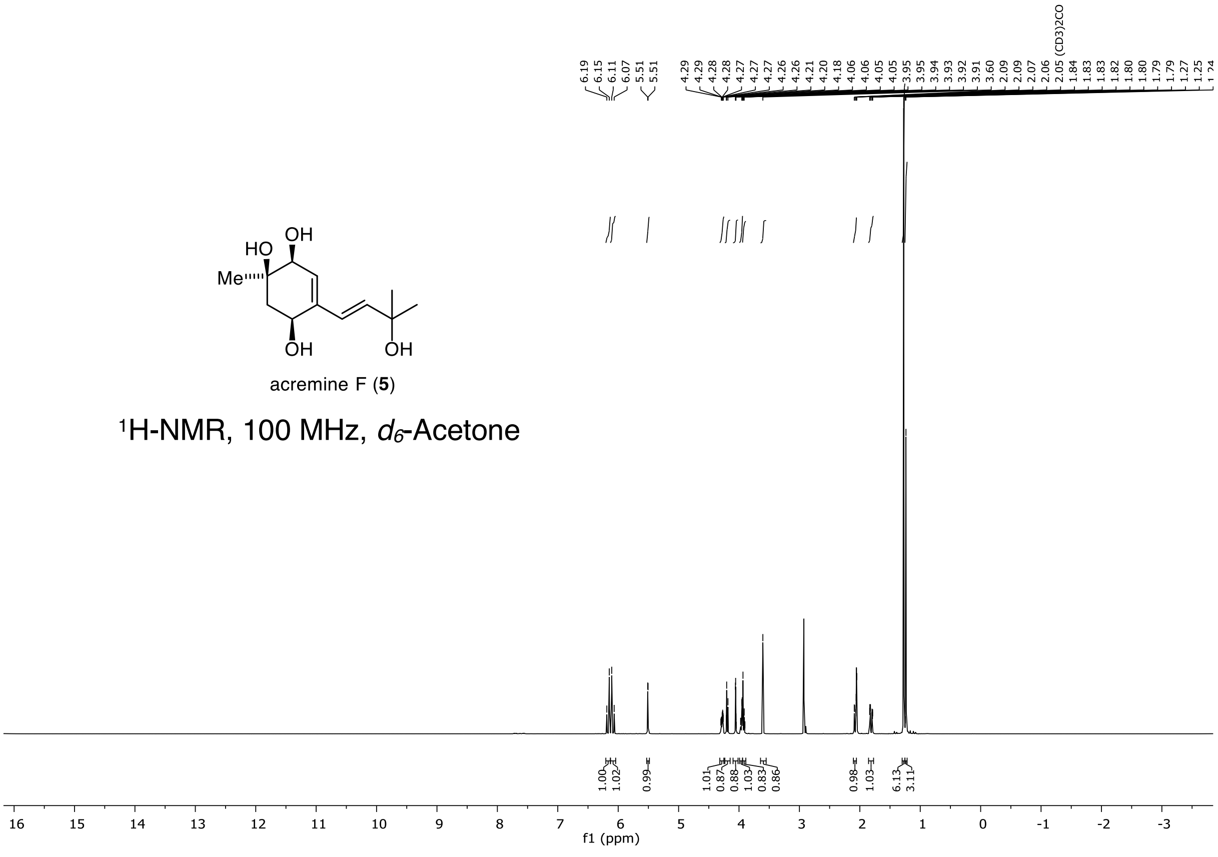

acremine F (**5**)

^{1}H-NMR, 100 MHz, d_6-Acetone

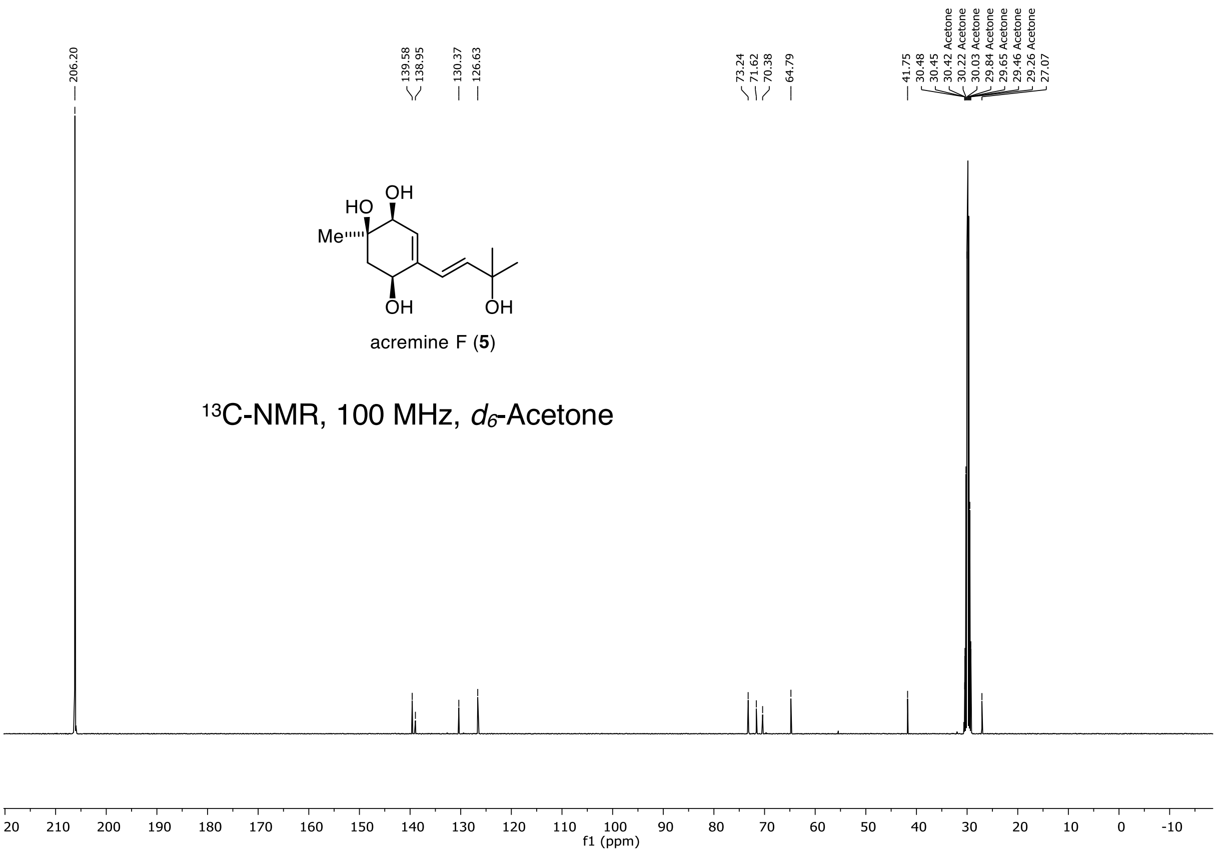

acremine F (**5**)

^{13}C-NMR, 100 MHz, d_6-Acetone

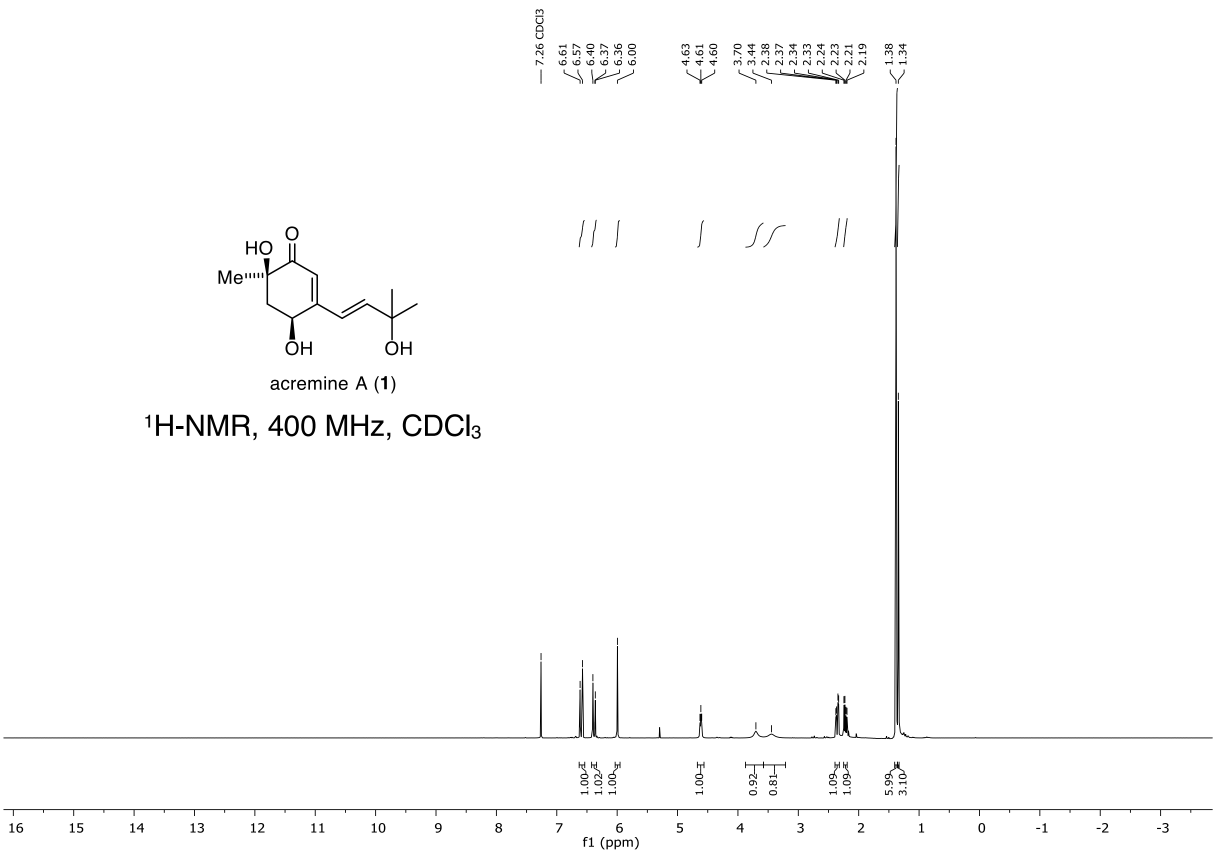

acremine A (**1**)

^{1}H-NMR, 400 MHz, $CDCl_3$

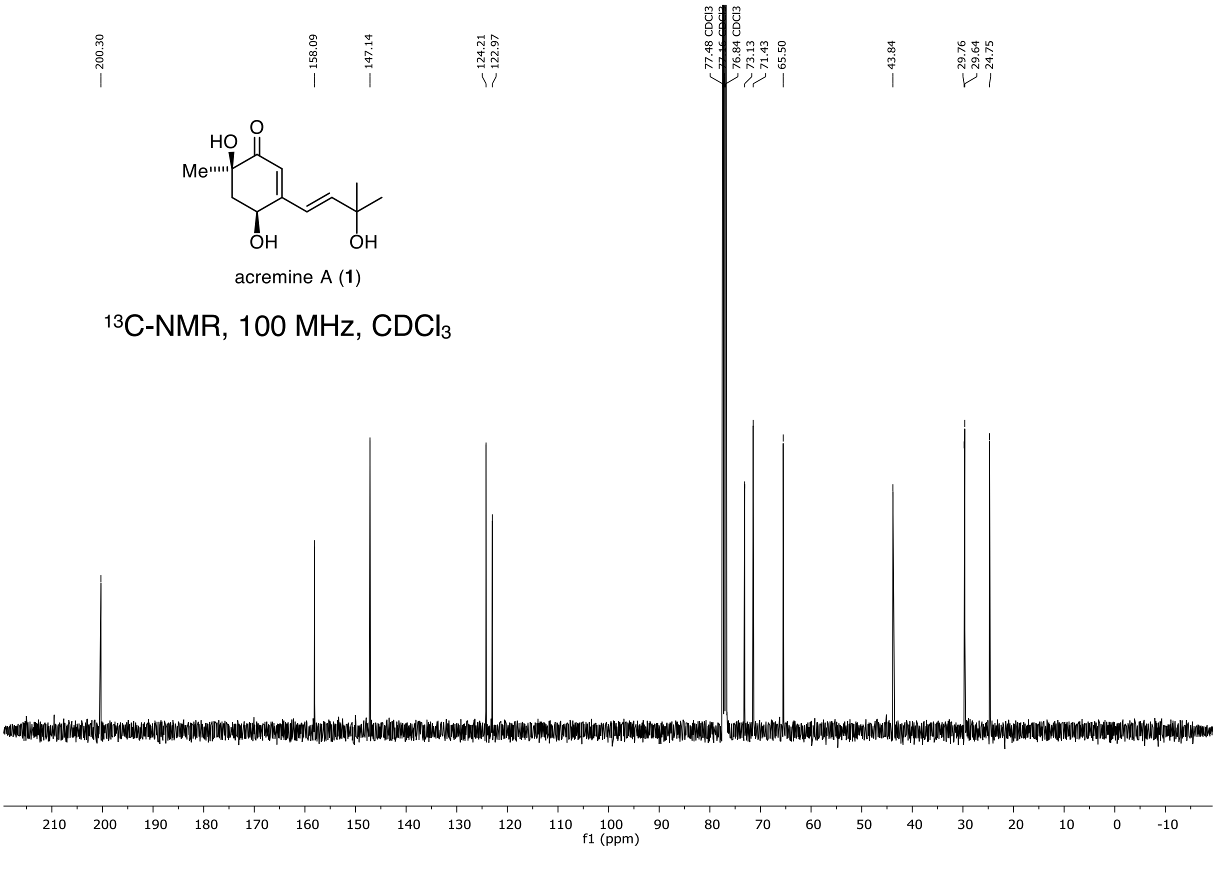

acremine A (**1**)

^{13}C-NMR, 100 MHz, $CDCl_3$

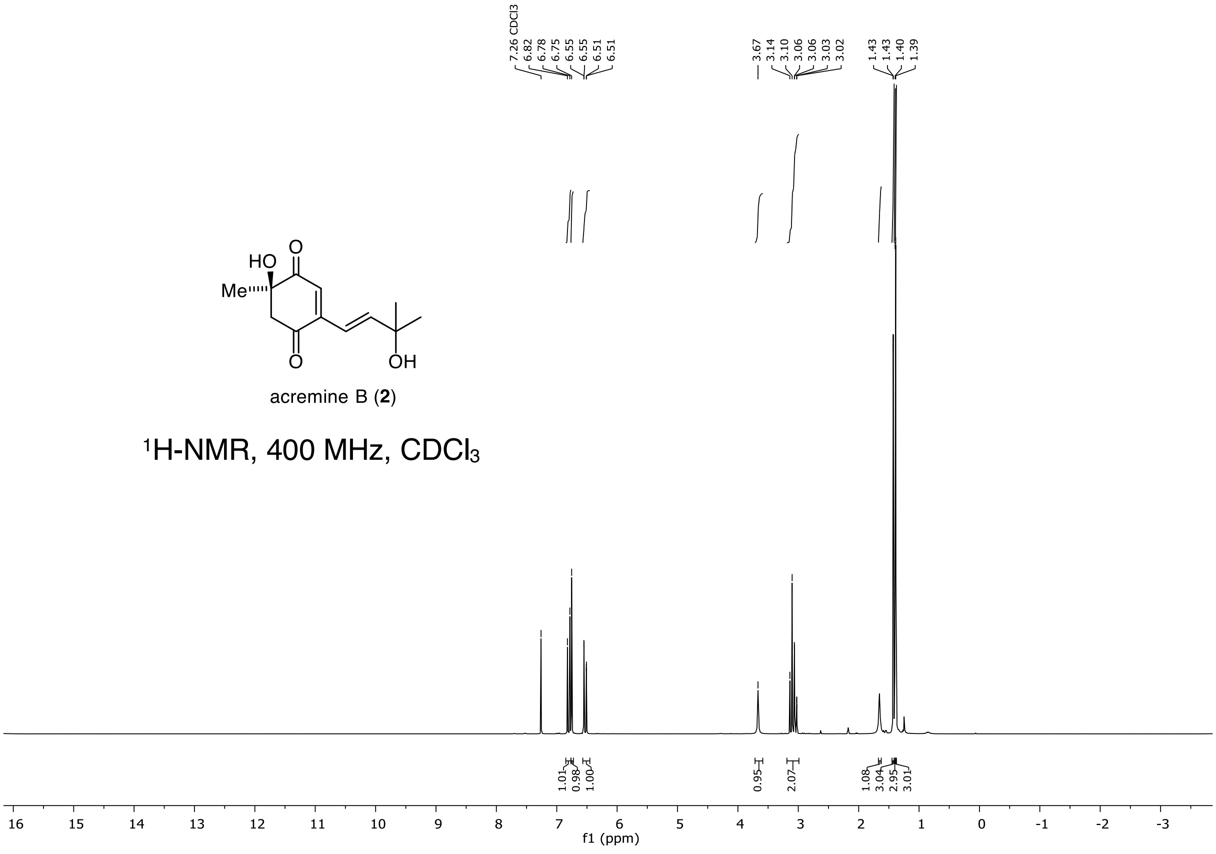

acremine B (**2**)

^{1}H-NMR, 400 MHz, $CDCl_3$

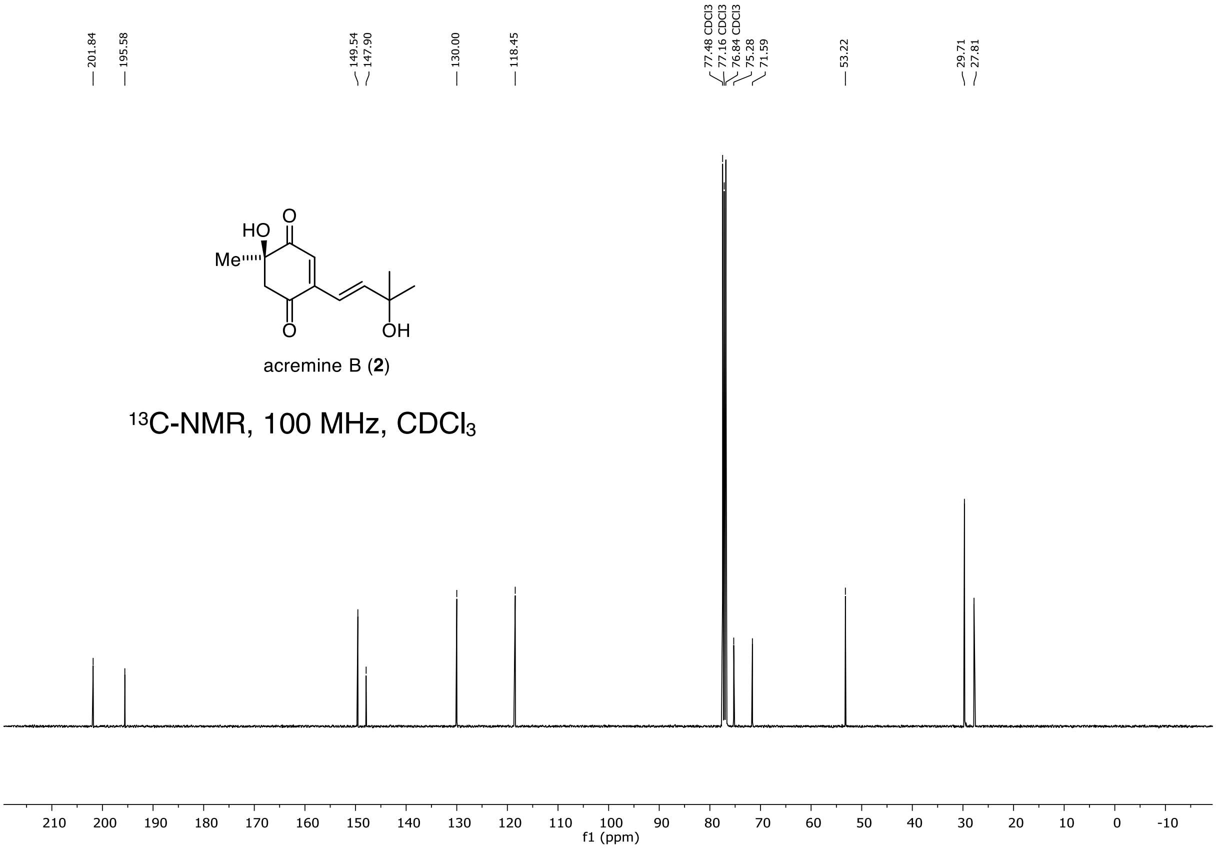

acremine B (**2**)

^{13}C-NMR, 100 MHz, $CDCl_3$

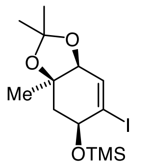

S1

^{1}H-NMR, 400 MHz, $CDCl_3$

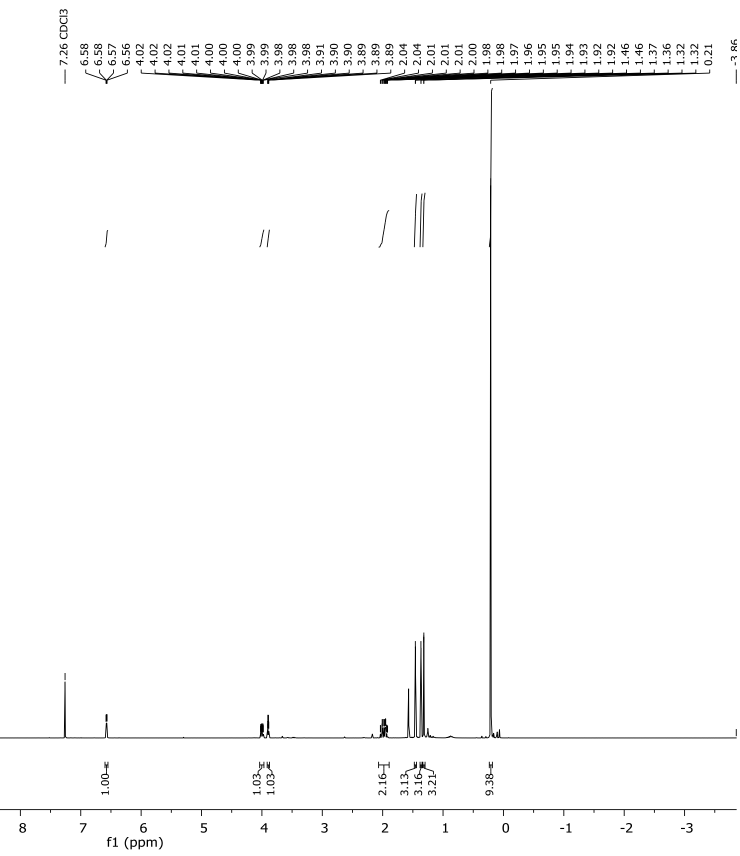

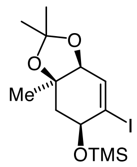

S1

^{13}C-NMR, 100 MHz, $CDCl_3$

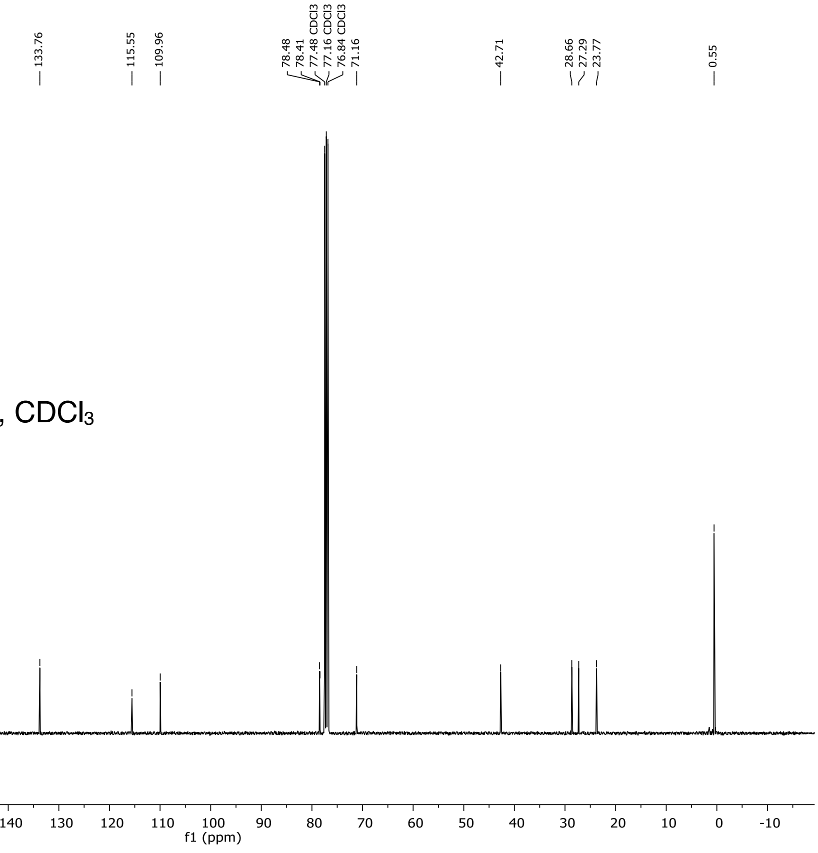

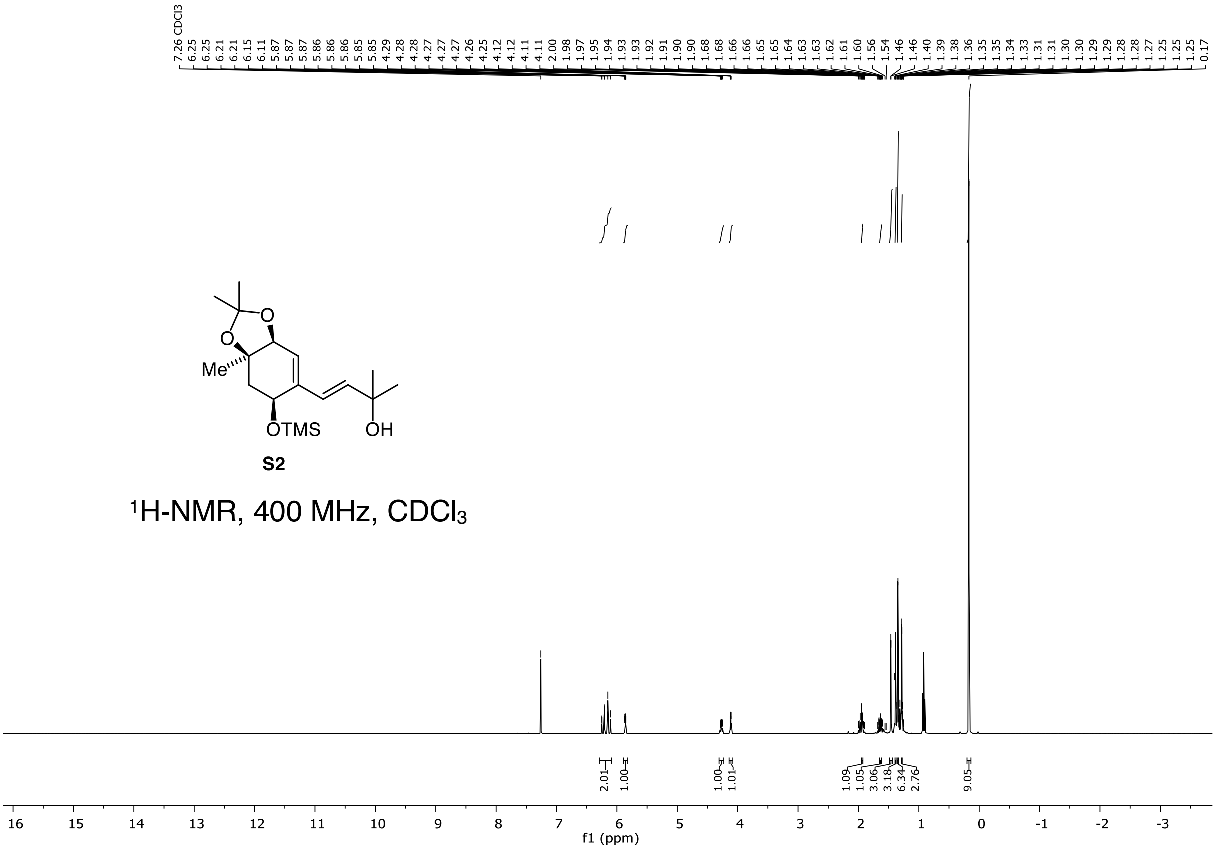

S2

^{1}H-NMR, 400 MHz, $CDCl_3$

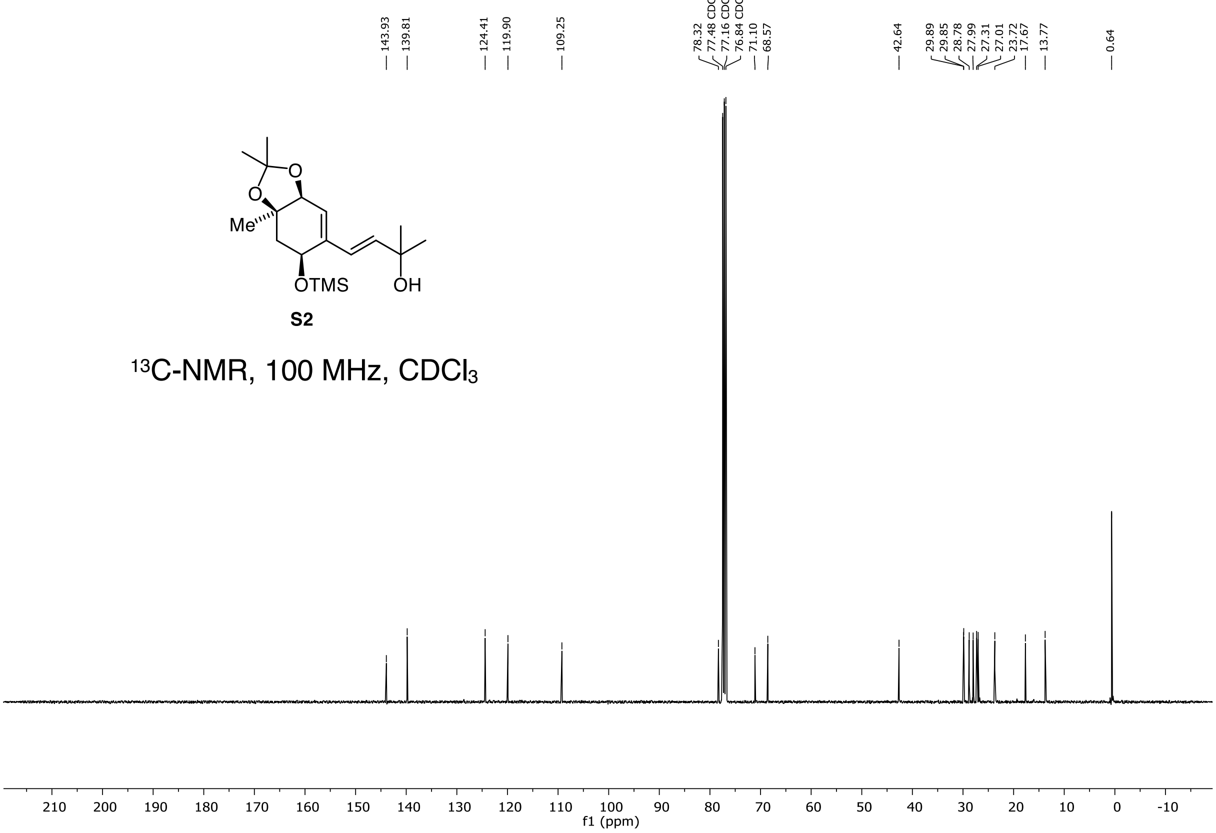

S2

^{13}C-NMR, 100 MHz, $CDCl_3$

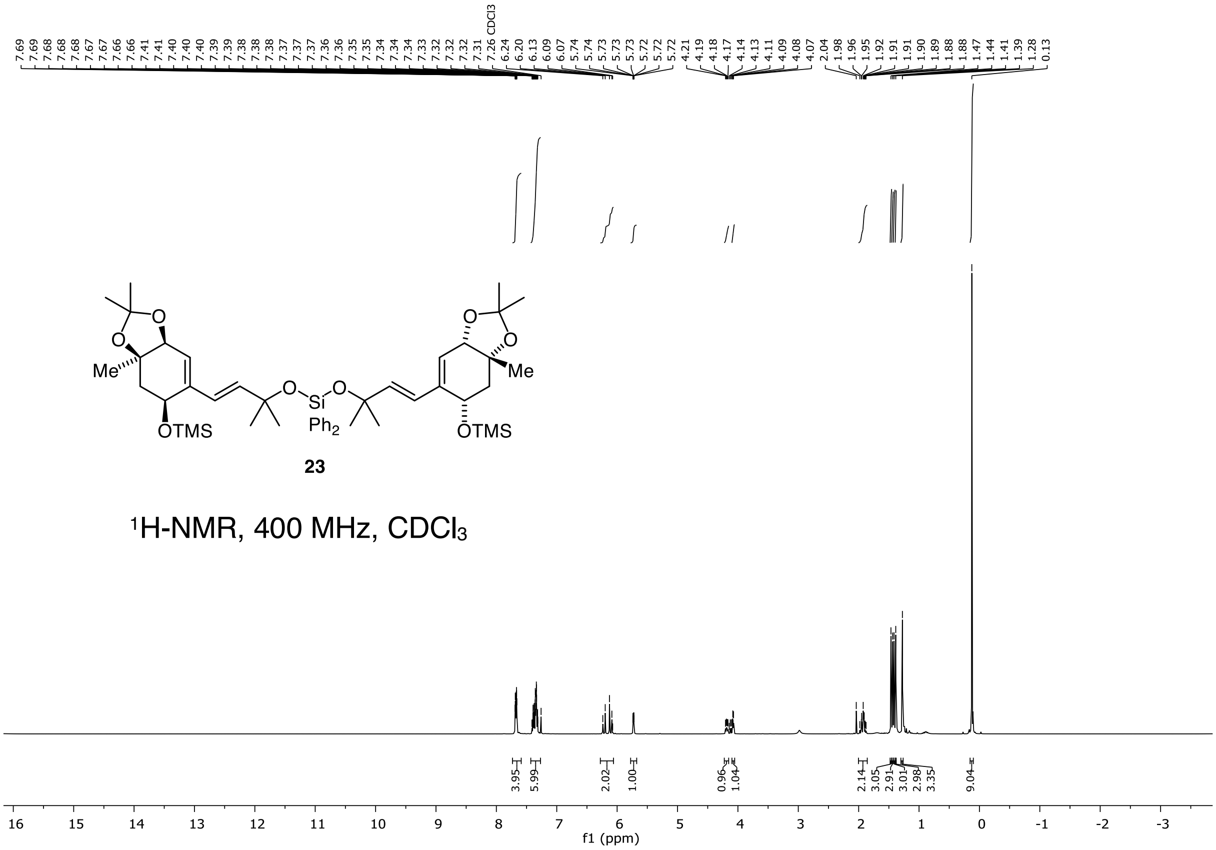
7.69 7.69 7.68 7.68 7.67 7.67 7.66 7.41 7.41 7.40 7.40 7.39 7.39 7.38 7.38 7.37 7.37 7.37 7.36 7.36 7.35 7.35 7.34 7.34 7.34 7.33 7.32 7.32 7.32 7.31 7.26 CDCl3 6.24 6.20 6.13 6.09 6.07 5.74 5.74 5.73 5.73 5.72 5.72 5.72 4.21 4.19 4.18 4.17 4.14 4.13 4.11 4.09 4.08 4.07 2.04 1.98 1.96 1.95 1.92 1.91 1.91 1.90 1.89 1.88 1.47 1.44 1.41 1.39 1.28 0.13
O
O
Me
OTMS
O
Si
O
Ph2
O
O
Me
OTMS
23
1H-NMR, 400 MHz, CDCl3
3.95 5.99 2.02 1.00 0.96 1.04 2.14 3.05 2.91 3.01 2.98 3.35 9.04
16 15 14 13 12 11 10 9 8 7 6 5 4 3 2 1 0 -1 -2 -3
f1 (ppm)

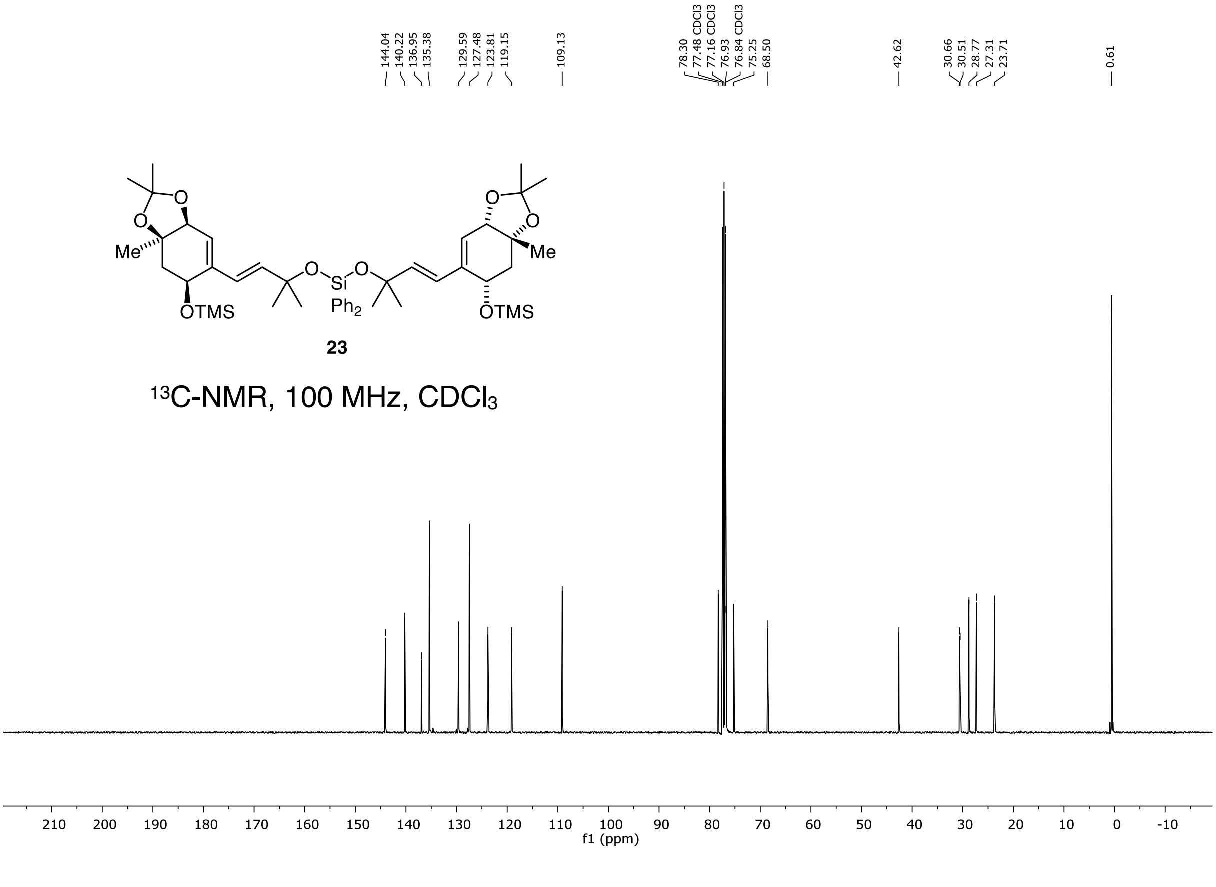
144.04 140.22 136.95 135.38 129.59 127.48 123.81 119.15 109.13 78.30 CDCl3 77.48 CDCl3 77.16 CDCl3 76.93 76.84 CDCl3 75.25 68.50 42.62 30.66 30.51 28.77 27.31 23.71 0.61
O
O
Me
OTMS
O
Si
O
Ph2
O
O
Me
OTMS
23
13C-NMR, 100 MHz, CDCl3
210 200 190 180 170 160 150 140 130 120 110 100 90 80 70 60 50 40 30 20 10 0 -10
f1 (ppm)

S3

^{1}H-NMR, 400 MHz, $CDCl_3$

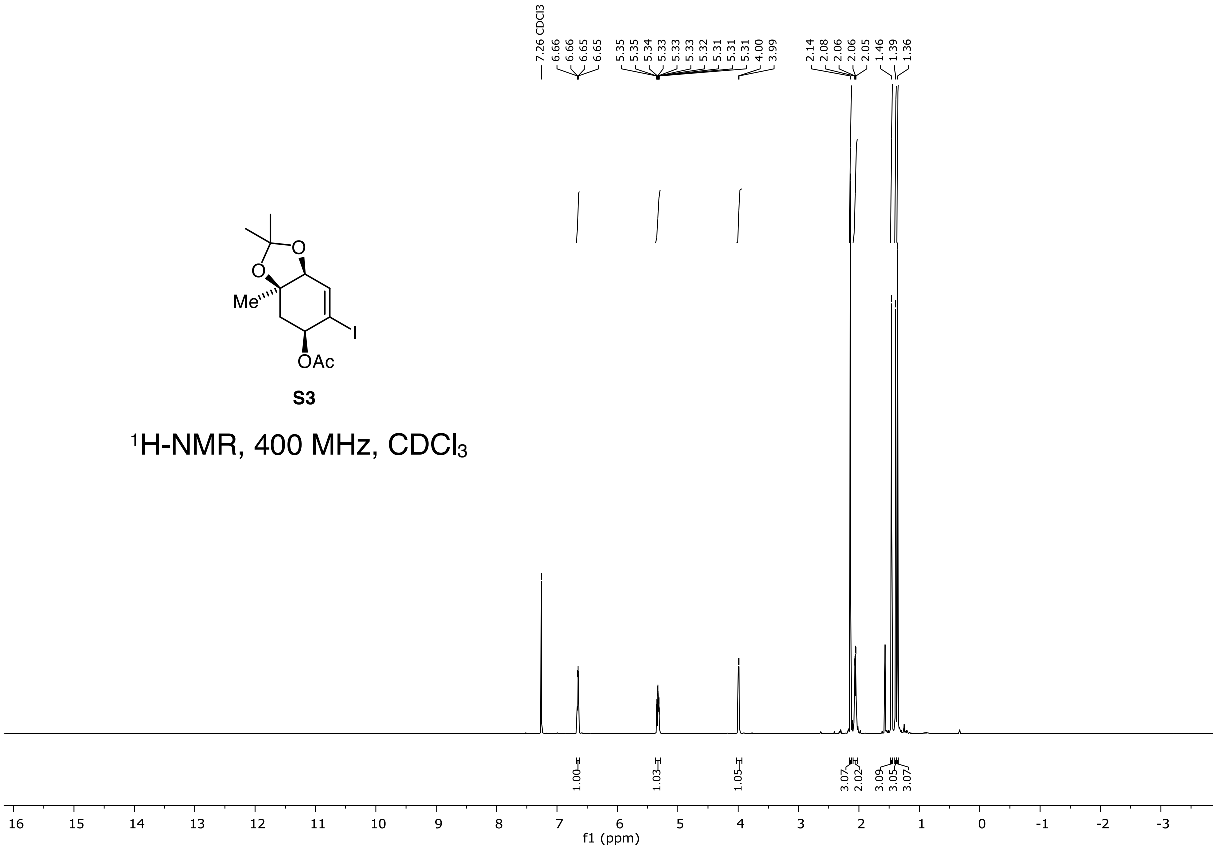

S3

^{13}C-NMR, 100 MHz, $CDCl_3$

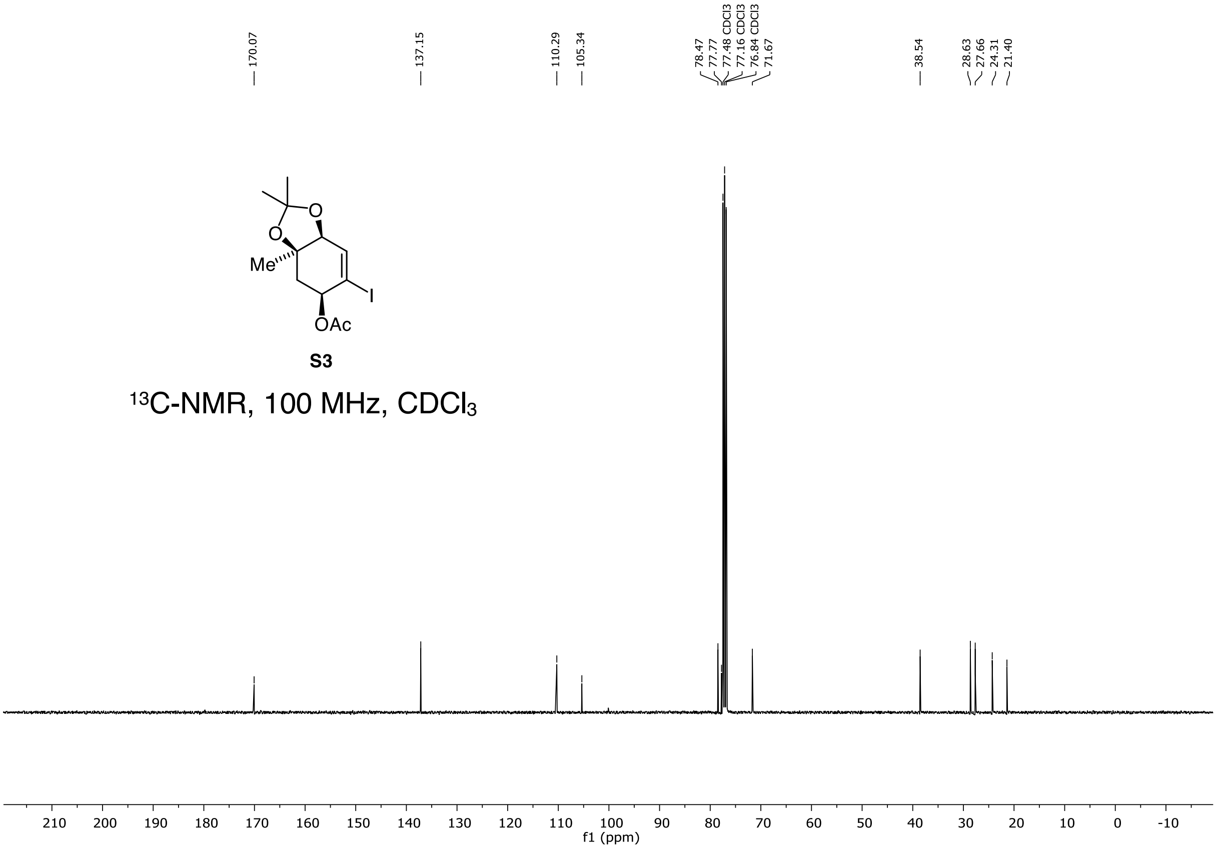

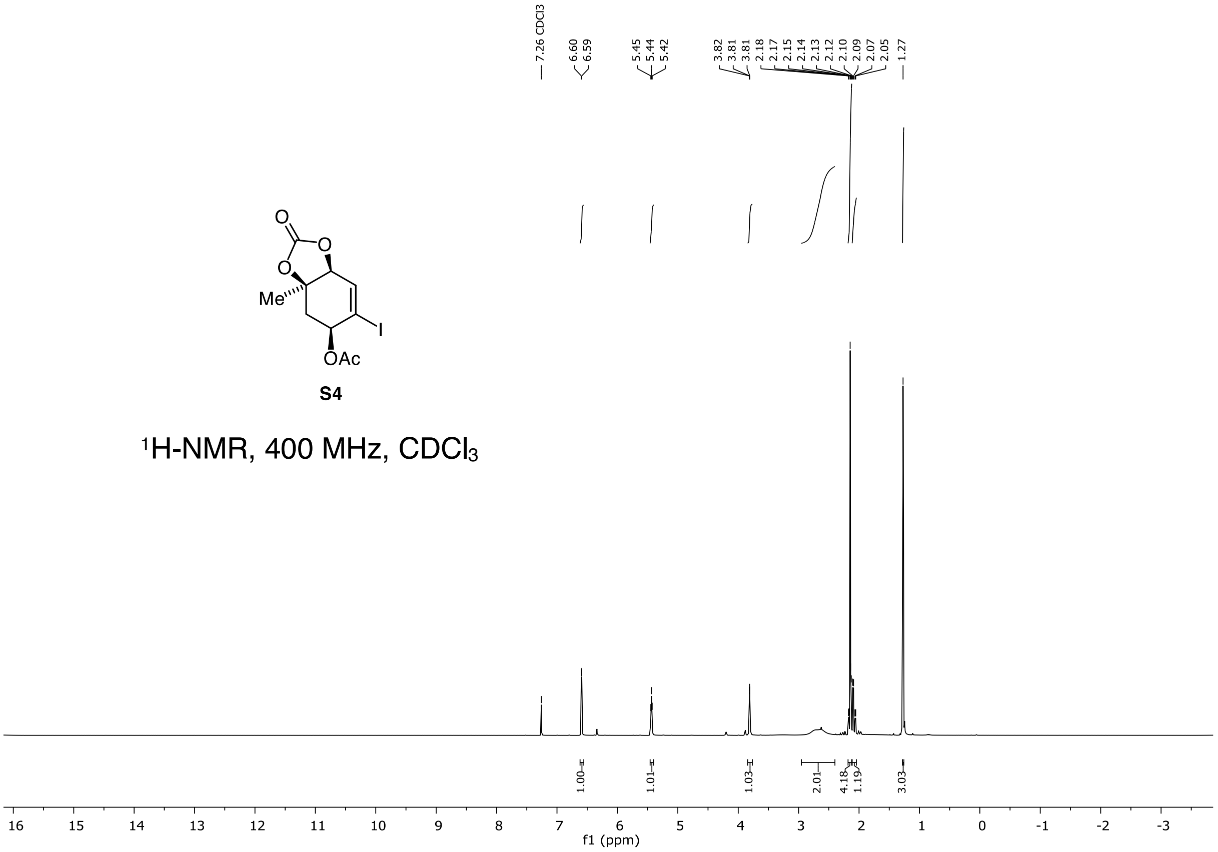
7.26 CDCl3
6.60
6.59
5.45
5.44
5.42
3.82
3.81
3.81
2.18
2.17
2.15
2.14
2.13
2.12
2.10
2.09
2.07
2.05
1.27
O
O
O
Me
I
OAc
S4
1H-NMR, 400 MHz, CDCl3
1.00
1.01
1.03
2.01
4.18
1.19
3.03
16
15
14
13
12
11
10
9
8
7
6
5
4
3
2
1
0
-1
-2
-3
f1 (ppm)

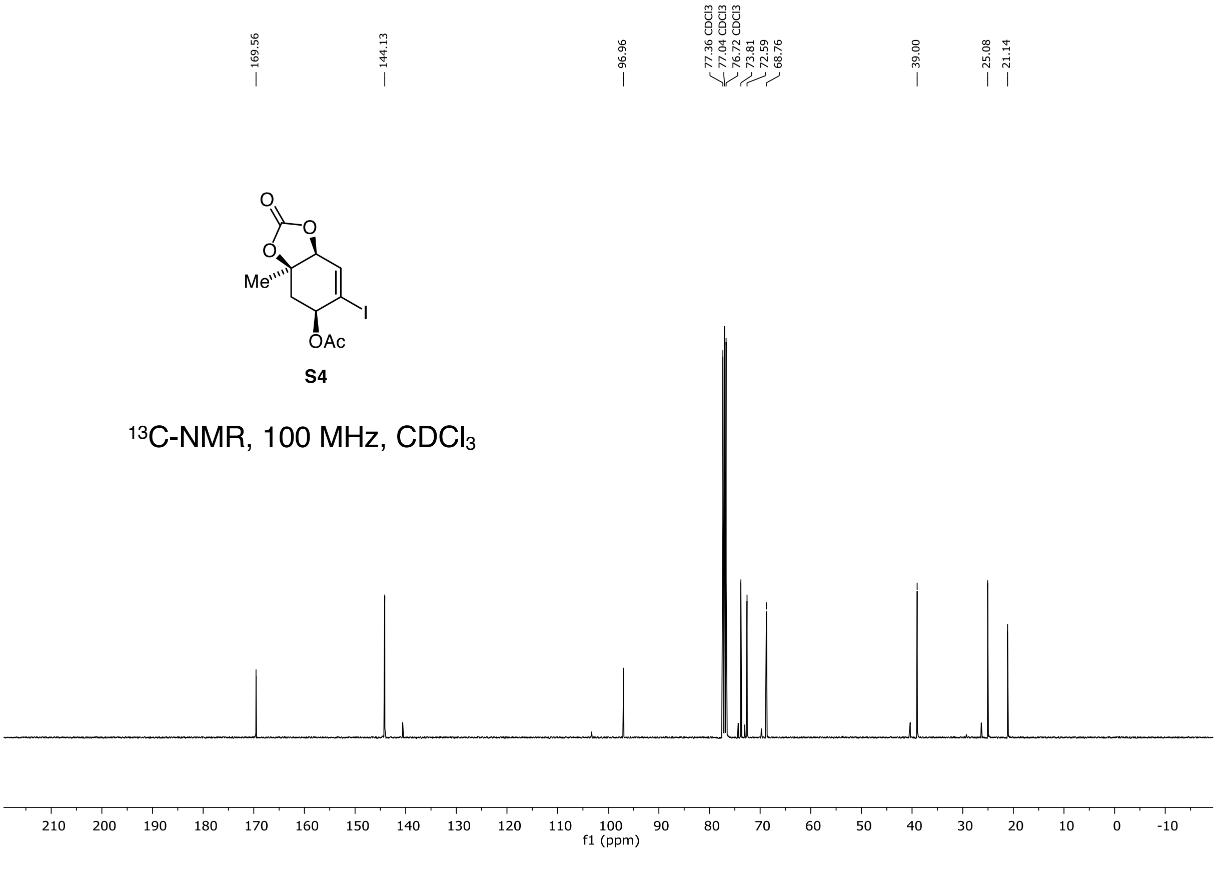
169.56
144.13
96.96
77.36 CDCl3
77.04 CDCl3
76.72 CDCl3
73.81
72.59
68.76
39.00
25.08
21.14
O
O
O
Me
I
OAc
S4
13C-NMR, 100 MHz, CDCl3
210
200
190
180
170
160
150
140
130
120
110
100
90
80
70
60
50
40
30
20
10
0
-10
f1 (ppm)

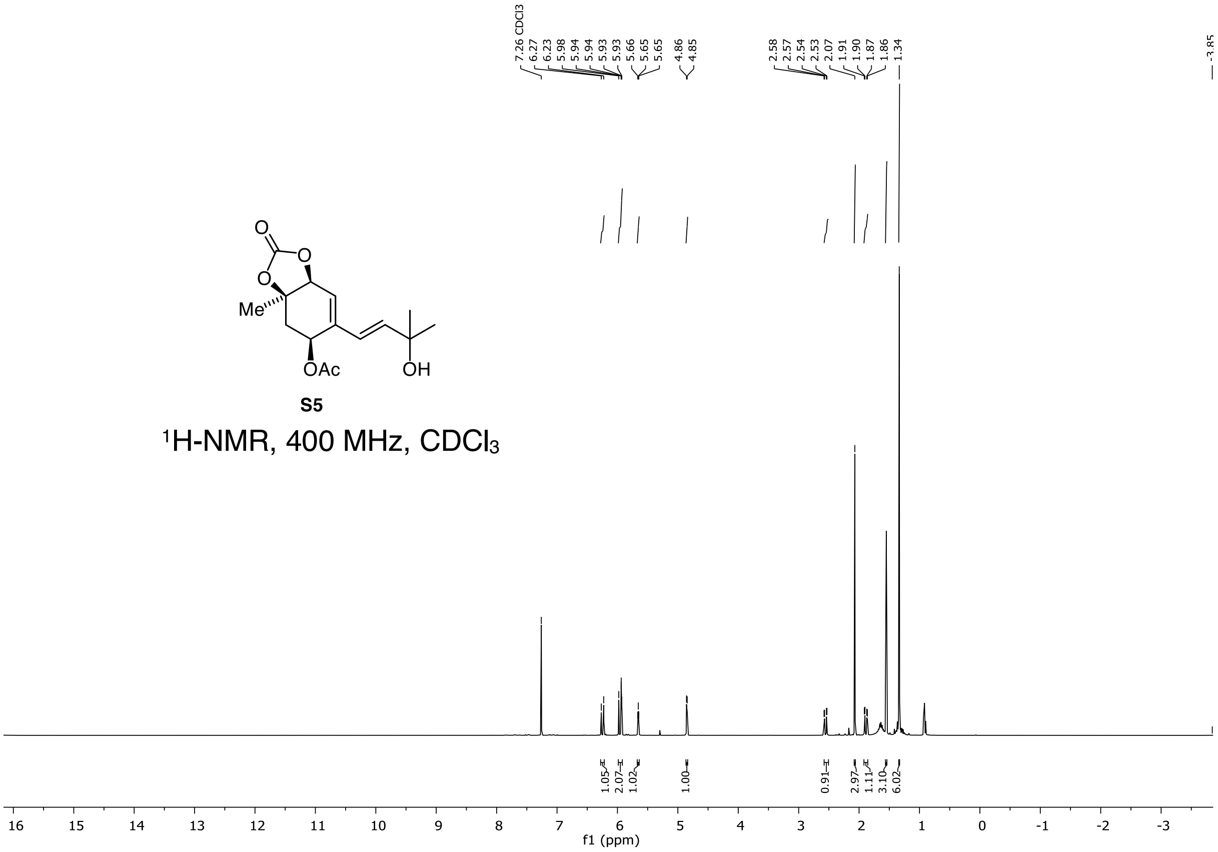

S5

^{1}H-NMR, 400 MHz, $CDCl_3$

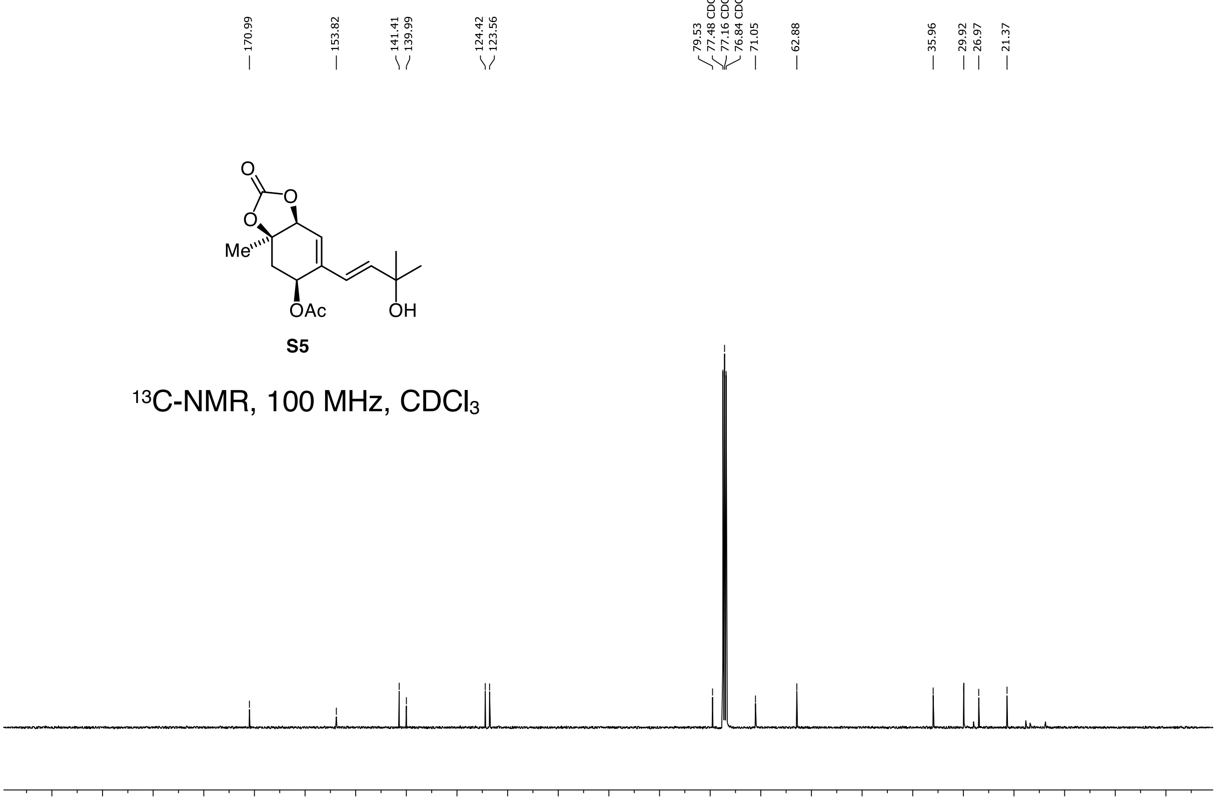

S5

^{13}C-NMR, 100 MHz, $CDCl_3$

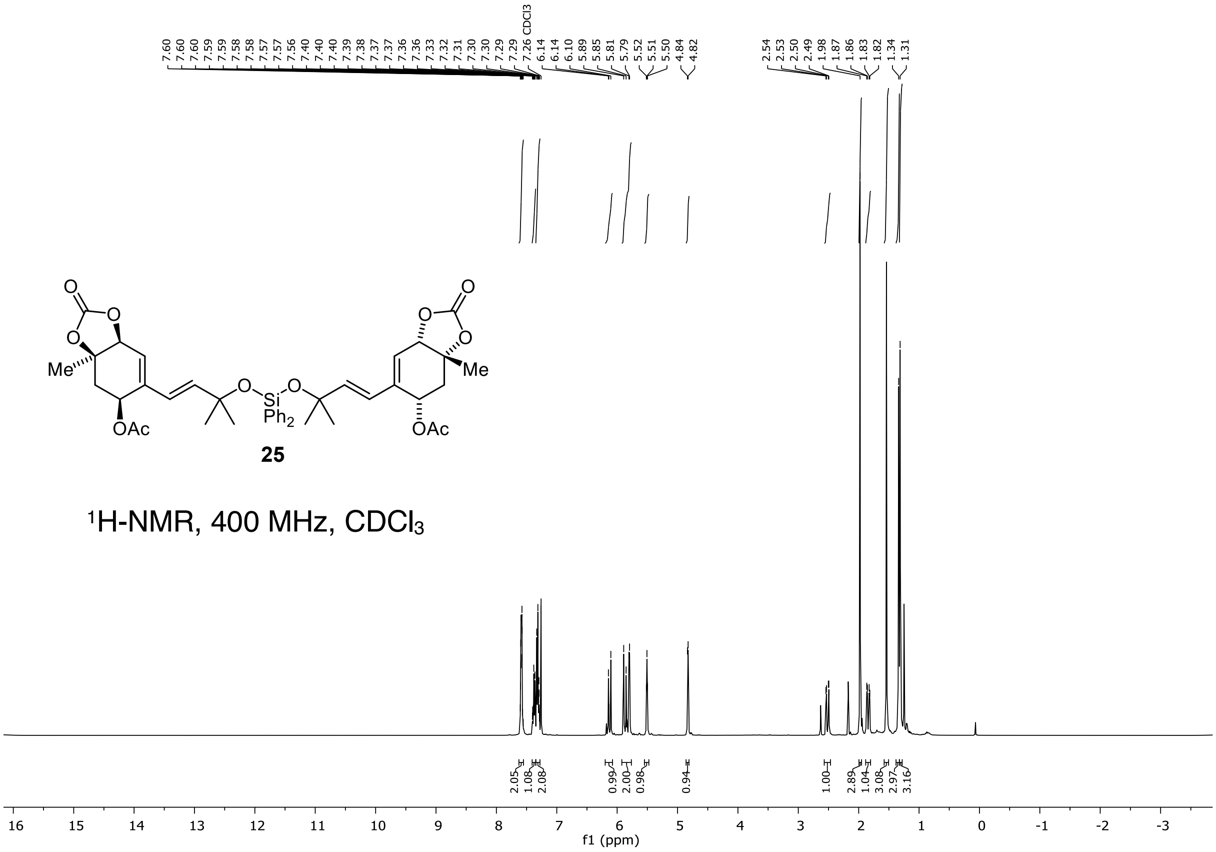
7.60 7.60 7.60 7.59 7.59 7.58 7.58 7.57 7.57 7.56 7.40 7.40 7.40 7.39 7.38 7.37 7.37 7.36 7.36 7.33 7.32 7.31 7.30 7.30 7.29 7.29 7.26 CDCl3 6.14 6.14 6.10 5.89 5.85 5.81 5.79 5.52 5.51 5.50 4.84 4.82
2.54 2.53 2.50 2.49 1.98 1.87 1.86 1.83 1.82 1.34 1.31
Me
OAc
Si
Ph2
25
1H-NMR, 400 MHz, CDCl3
2.05 1.08 2.08 0.99 2.00 0.98 0.94 1.00 2.89 1.04 3.08 2.97 3.16
f1 (ppm)

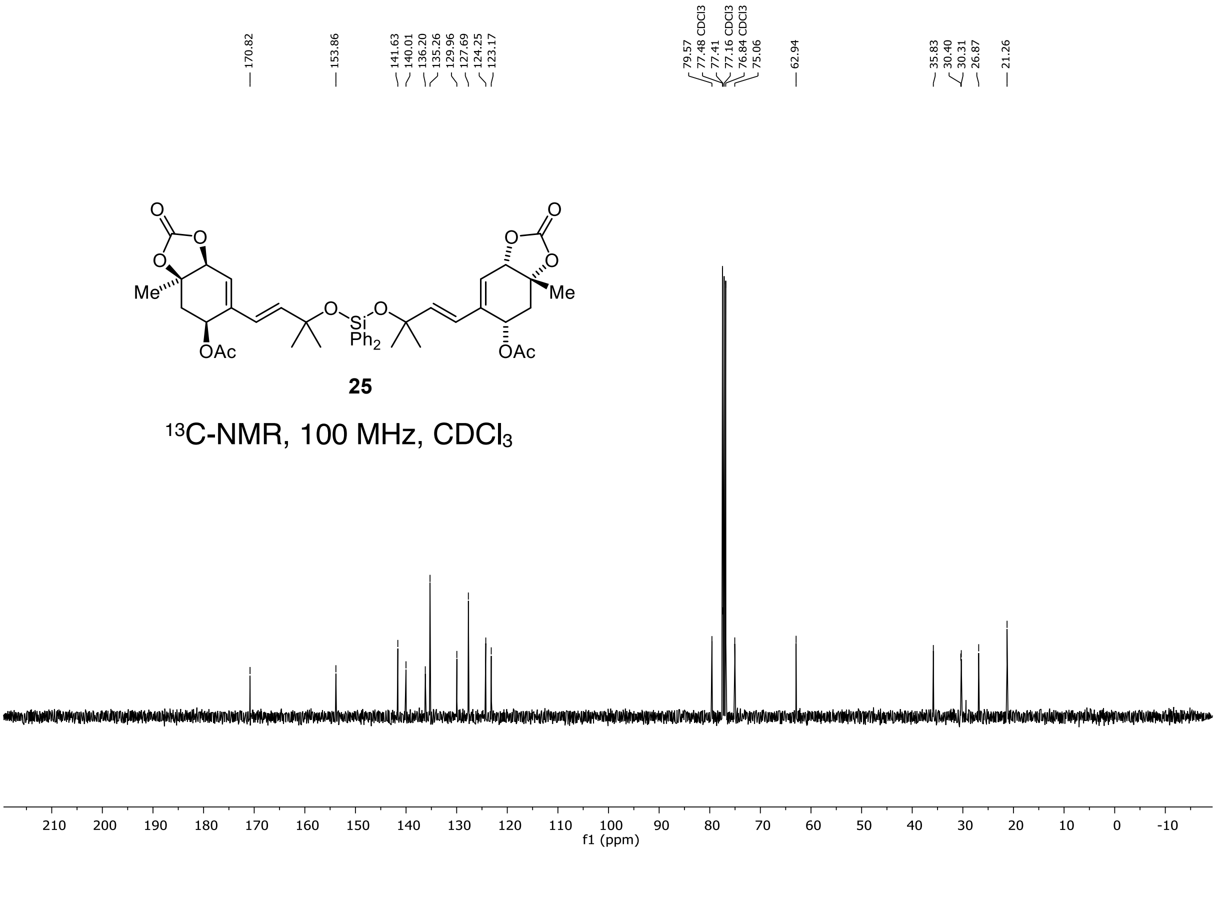
170.82
153.86
141.63 140.01 136.20 135.26 129.96 127.69 124.25 123.17
79.57 77.48 CDCl3 77.41 77.16 CDCl3 76.84 CDCl3 75.06
62.94
35.83 30.40 30.31 26.87
21.26
Me
OAc
Si
Ph2
25
13C-NMR, 100 MHz, CDCl3
f1 (ppm)

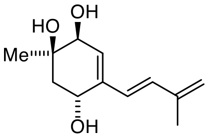

6

^{1}H-NMR, 400 MHz, d_4-MeOH

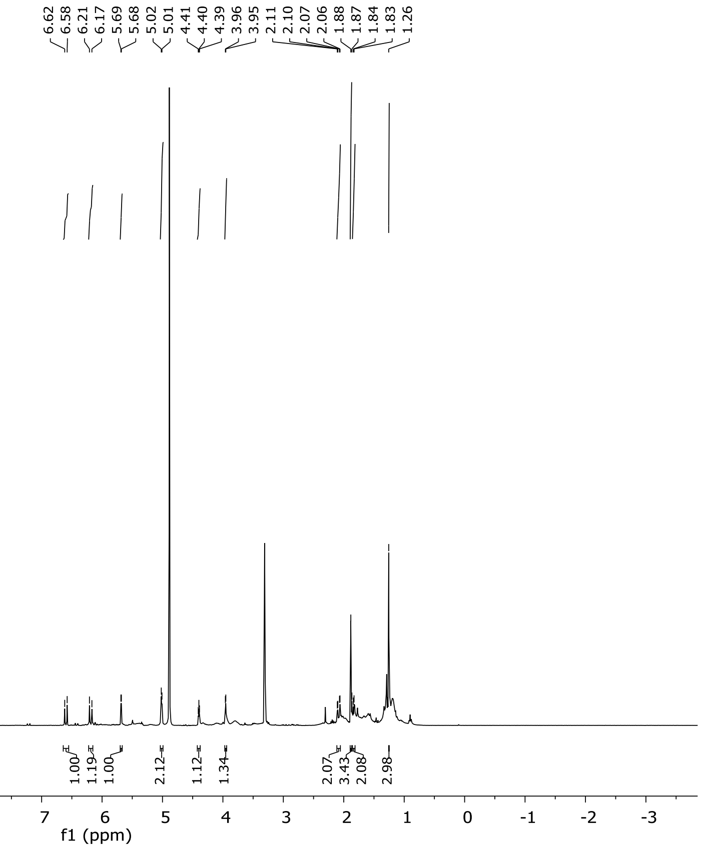

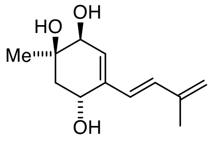

6

^{13}C-NMR, 100 MHz, d_4-MeOH

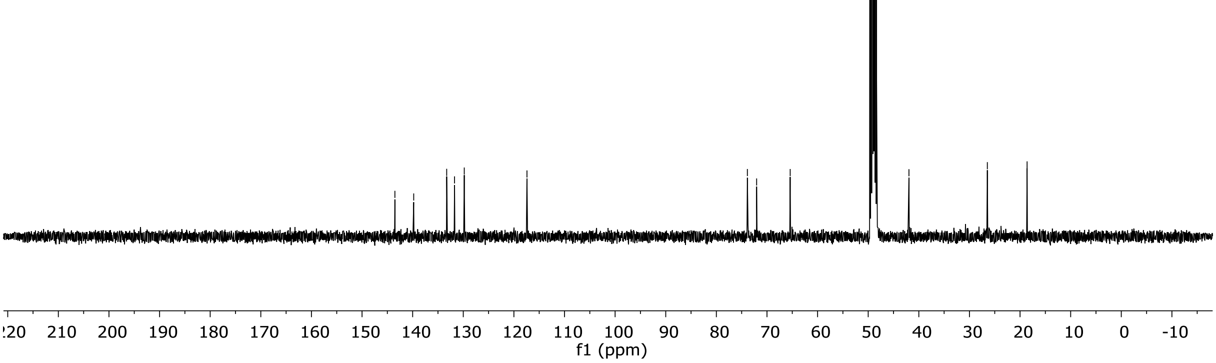

Crystallographic Data

- **Enone 16 (CCDC 1854563)**

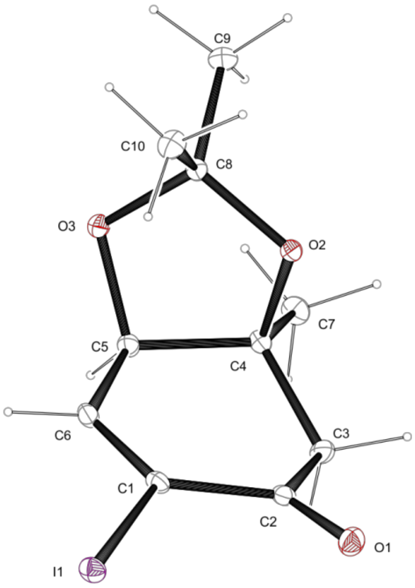

Figure 1. ORTEP of the molecular structure of enone **16**.

Table 4. Crystallographic data for enone 16.

net formula	$C_{10}H_{13}IO_3$
M_r/g mol^{-1}	308.10
crystal size/mm	0.100 × 0.080 × 0.050
T/K	100.(2)
radiation	MoKα
diffractometer	'Bruker D8 Venture TXS'
crystal system	monoclinic
space group	'P 1 21/c 1'
a/Å	5.5298(4)
b/Å	12.2048(10)
c/Å	16.1544(12)
α/°	90
β/°	94.678(2)
γ/°	90
V/Å^3	1086.63(14)
Z	4
calc. density/g cm^{-3}	1.883
μ/mm^{-1}	2.927
absorption correction	Multi-Scan
transmission factor range	0.5958–0.6367
refls. measured	20258
R_{int}	0.0530
mean σ(I)/I	0.0329
θ range	3.338–30.501
observed refls.	3010
x, y (weighting scheme)	0.0244, 0.5259

hydrogen refinement	constr
refls in refinement	3298
parameters	130
restraints	0
$R(F_{obs})$	0.0212
$R_w(F^2)$	0.0561
S	1.043
shift/error$_{max}$	0.001
max electron density/e Å^{-3}	0.902
min electron density/e Å^{-3}	–0.570

- **Allylic alcohol 17 (CCDC 1854564)**

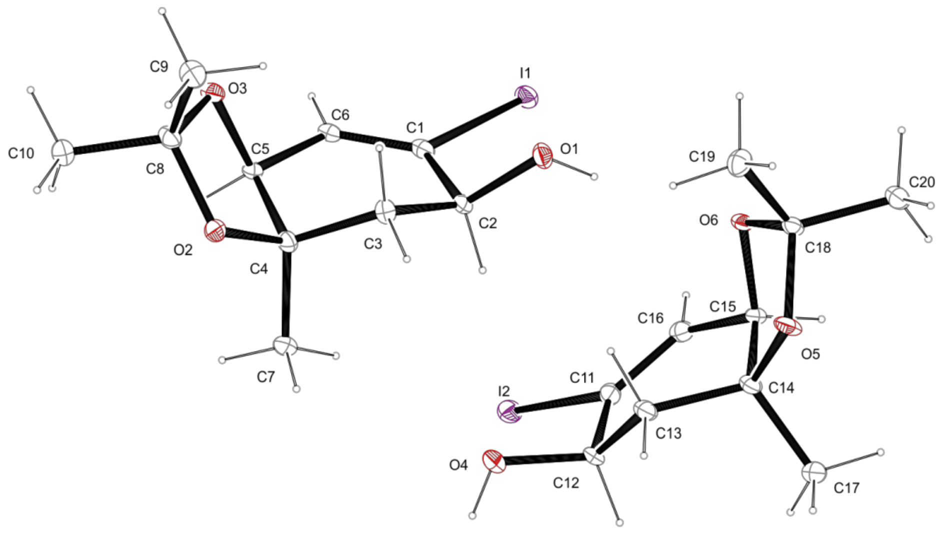

Figure 2. ORTEP of the molecular structure of allylic alcohol **17**.

Table 5. Crystallographic data for allylic alcohol 17.

net formula	$C_{10}H_{15}IO_3$
M_r/g mol^{-1}	310.12
crystal size/mm	0.100 × 0.080 × 0.020
T/K	100.(2)
radiation	MoKα
diffractometer	'Bruker D8 Venture TXS'
crystal system	triclinic
space group	'P -1'
a/Å	8.0873(5)
b/Å	12.0960(9)
c/Å	12.4087(9)
α/°	104.002(2)
β/°	103.200(2)
γ/°	92.314(2)
V/Å^3	1140.79(14)
Z	4
calc. density/g cm^{-3}	1.806

μ/mm^{-1}	2.788
absorption correction	Multi-Scan
transmission factor range	0.5733–0.7454
refls. measured	19827
R_{int}	0.0403
mean σ(*I*)/*I*	0.0341
θ range	3.158–26.372
observed refls.	4077
x, y (weighting scheme)	0.0417, 0.8914
hydrogen refinement	constr
refls in refinement	4630
parameters	261
restraints	0
$R(F_{obs})$	0.0286
$R_w(F^2)$	0.0756
S	1.063
shift/error$_{max}$	0.001
max electron density/e Å^{-3}	1.314
min electron density/e Å^{-3}	−1.036

References

1. Woo, C, M.; Lu, L.; Gholap, S. L.; Smith, D. R.; Herzon, S. B. *J. Am. Chem. Soc.,* **2010**, *132*, 2540–2541.
2. Assante, G.; Dallavalle, S.; Malpezzi, L.; Nasini, G.; Burruanod, S.; Tortad, L. *Tetrahedron*, **2005**, *61,* 7686–7692.
3. Suciati; Fraser, J. A.; Lambert, L. K.; Pierens, G. K.; Bernhardt, P. V.;. Garson, M. J. *J. Nat. Prod.*, **2013**, *76*, 1432–1440.
4. Wu, P.; Yao, L.; Xu, L.; Xue, L.; Wie, X. *J. Nat. Prod.* **2015**, 78, 2161–2166.
